# Lactobacilli-Derived Microbe-Associated Molecular Patterns (MAMPs) in Host Immune Modulation

**DOI:** 10.3390/biom15111609

**Published:** 2025-11-17

**Authors:** Salvatore Furnari, Ruben Ciantia, Adriana Garozzo, Pio Maria Furneri, Virginia Fuochi

**Affiliations:** Department of Biomedical and Biotechnological Sciences (BIOMETEC), University of Catania, 95123 Catania, Italy; salvatore.furnari@phd.unict.it (S.F.); agar@unict.it (A.G.); pio.furneri@unict.it (P.M.F.)

**Keywords:** host–microbe interactions, microorganism-associated molecular patterns (MAMPs), Toll-like receptors (TLRs), immunomodulation, immune response, inflammatory response, pattern recognition receptors (PRRs)

## Abstract

Although traditionally sidelined by live probiotic effects, Lactobacilli-derived Microbe-Associated Molecular Patterns (MAMPs) are emerging as potent modulators of innate and adaptive immune responses, capable of acting independently of bacterial viability. However, the underlying mechanisms remain incompletely understood. These MAMPs, such as peptidoglycan (PGN), lipoteichoic acid (LTA), and exopolysaccharides (EPSs), interact with pattern recognition receptors (PRRs) like Toll-like receptors (TLRs), initiating immune-signaling cascades that regulate cytokine production and inflammation. Lactobacilli-derived MAMPs exhibit dual immunomodulatory effects: they can enhance pro-inflammatory responses, e.g., interleukin-1β (IL-1β), IL-6, and tumor necrosis factor alpha (TNF-α) under inflammatory contexts, while enhancing regulatory pathways via IL-10 and regulatory T-cell (T_regs_) induction in anti-inflammatory settings. Importantly, these immunomodulatory properties persist in the absence of bacterial viability, making MAMPs promising candidates for postbiotic therapies. This opens new avenues for MAMP-based strategies to target inflammation, overcoming the risks associated with live bacterial administration. This review examines the therapeutic relevance of non-viable MAMPs, particularly in inflammatory diseases where they have demonstrated benefits in reducing tissue damage, enhancing gut barrier function, and alleviating disease symptoms. Additionally, we discuss regulatory and translational challenges hindering their clinical implementation, highlighting the need for standardized characterization, a clear safety framework, and strain-specific profiling. Given their ability to fine-tune immune responses, MAMPs represent an emerging strategy for innovative treatments aimed at restoring immune balance and reinforcing host–microbe interactions.

## 1. Introduction

The family *Lactobacillaceae* (for simplicity, the term “lactobacilli” will hereafter be used to collectively refer to all members of this family) comprises Gram-positive, non-spore-forming, facultatively anaerobic bacteria belonging to the phylum Firmicutes, currently encompassing over 260 species across 25 genera [1,2]. Lactobacilli constitute a taxonomically and phylogenetically diverse group of bacteria that colonizes multiple mucosal surfaces, including gastrointestinal, oral, and genitourinary tracts. At these interfaces, lactobacilli contribute to mucosal immune education and microbial homeostasis through mechanisms that go far beyond passive colonization, influencing both immune tolerance and pathogen resistance [3,4,5].

Beyond the direct suppression of pathogens, lactobacilli exert a broad spectrum of immunological effects that influence innate and adaptive responses, contributing to mucosal homeostasis and reducing the risk of chronic inflammatory diseases [3,6]. These effects are mediated, in part, by the production of antimicrobial and signaling molecules such as bacteriocins, reactive oxygen species (ROS), and organic acids that shape both microbial composition and host responses [7,8,9,10]. Furthermore, lactobacilli enhance intestinal epithelial barrier integrity, engage in the competitive exclusion of pathogens, and exert oncopreventive and antiproliferative effects, establishing them as valuable components of probiotic formulations [1,11,12]. These functional attributes justify their widespread use as probiotics, defined by the International Scientific Association for Probiotics and Prebiotics (ISAPP) as “live microorganisms that, when administered in adequate amounts, confer health benefits on the host” [13]. Supported by their Generally Recognized as Safe (GRAS) status [14] and decades of clinical applications, lactobacilli are today incorporated into a variety of probiotic formulations, including foods containing lactobacilli and their metabolites, such as yogurt. Notably, evidence suggests that their beneficial effects may extend beyond the gut, including effects on extra-intestinal carcinogenesis, underscoring their potential role in cancer prevention and overall health improvement [14,15,16,17].

While these effects have traditionally been attributed to the activity of live probiotic bacteria, several studies now demonstrate that inactivated cells, structural components, and secreted molecules can exert comparable functions [3,18,19]. This has led to the ISAPP definition of postbiotics as “preparations of inanimate microorganisms and/or their components that confer a health benefit on the host” [20]. Among these components, MAMPs have been identified as biologically active mediators that may account for many of the immune-regulatory properties previously ascribed to viable lactobacilli [21].

A key element of their immunoregulatory capacity is the production of MAMPs, detected by host PRRs, including TLRs and NOD-like receptors (NLRs). As summarized in Table S1, key lactobacilli-derived MAMPs include lipoteichoic acid (LTA), peptidoglycan (PGN), and exopolysaccharides (EPSs). These molecules vary structurally among strains, yet converge functionally in modulating immune signaling pathways [22,23]. The recognition of these components by TLR2 and NOD2 has been shown to activate downstream signaling cascades primarily through NF-κB and Mitogen-Activated Protein Kinase (MAPK) pathways, leading to an increased secretion of anti-inflammatory cytokines like IL-10, while reducing the levels of pro-inflammatory mediators including TNF-α and IL-6 [24,25]. Moreover, these signals modulate the activity of dendritic cells (DCs) and other antigen-presenting cells (APCs), resulting in an enhanced induction and differentiation of T_regs_, a mechanism essential for maintaining mucosal immune tolerance and preventing autoimmune responses [26].

This review examines how lactobacilli-derived MAMPs regulate host immunity by engaging PRRs and initiating signaling cascades that affect both innate and adaptive responses. The molecular interactions between these bacterial components and host pattern recognition receptors are described, with a particular focus on the downstream signaling pathways involved in cytokine modulation, T_reg_ induction, and T helper (T_h_) cells. Furthermore, the therapeutic potential of lactobacilli-derived MAMPs in clinical settings is discussed, highlighting their potential as non-viable immunomodulatory agents and addressing current limitations to clinical application. The aim is to clarify the mechanisms by which MAMPs contribute to host immune regulation and to provide a rationale for their integration into postbiotic-based strategies for immune-mediated diseases.

## 2. Materials and Methods

This narrative review was conducted through a comprehensive literature search, primarily in MEDLINE, guided by the review’s focus on immunomodulation. No publication date restrictions were applied, ensuring a broad selection of studies. However, references were included based on their relevance to lactobacilli-derived MAMPs and their interactions with the host immune system.

The main search terms included “*Lactobacillus* MAMPs,” “innate immunity,” “adaptive immunity,” “cytokine modulation,” “regulatory T cells,” “pattern recognition receptors,” “T_h_1/T_h_2 differentiation,” “probiotic properties,” “GRAS status,” “inflammatory bowel disease,” and “autoimmune diseases.” Studies were selected if they provided a detailed exploration of immune modulation mechanisms. Additionally, given the importance of lactobacilli probiotics in immunology, both clinical and preclinical studies on their immunological impact were included.

The focus on cytokines and immune pathways was restricted to those that act as key regulators of inflammation and immune tolerance, ensuring the review concentrated on anti-inflammatory mechanisms and immune regulation. Furthermore, clinically relevant studies were included to provide a comprehensive overview of the therapeutic potential of lactobacilli-derived MAMPs in immune-mediated diseases.

A total of 587 records were initially identified, of which 300 met the inclusion criteria and were included.

## 3. Lactobacilli and the Art of Immune Modulation

The hypothesis that microbial populations could influence host physiology and longevity was first formulated in the early 20th century. Élie Metchnikoff proposed that lactic acid bacteria derived from fermented dairy products contributed to health maintenance by suppressing the proteolytic bacteria responsible for intestinal putrefaction, thus prolonging life expectancy [27]. This early concept, rooted in the demographic observation of Eastern European rural populations, provided the foundational rationale for subsequent probiotic research.

**Figure 1:**
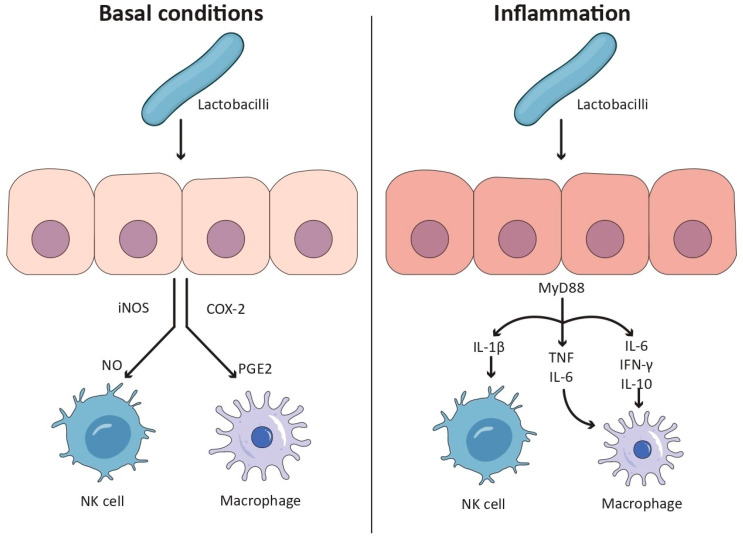
Immunomodulation by lactobacilli under basal conditions and during inflammation. **Left (basal conditions):** tonic epithelial sensing leads to modest iNOS and COX2 induction, generating NO and PGE_2_ that support controlled activity of NK cells and macrophages, thereby maintaining mucosal homeostasis. **Right (inflammation):** in an inflamed context, TLR signaling via MyD88 enhances epithelial release of IL-1β, TNF, and IL-6, reshaping innate responses; macrophages produce IL-6, IFN-γ, and IL-10, integrating pro-inflammatory and regulatory cues. Effects are strain-specific and condition-dependent, varying with the host’s physiological and immunological state.

In the decades that followed, the immunological relevance of commensal and probiotic microorganisms, particularly lactobacilli, was increasingly recognized. As shown in Figure 1, these bacteria were shown to interact with the host immune system, influencing both mucosal and systemic responses. Their effects were described as highly strain-dependent and conditional, varying according to the physiological and immunological state of the host [28]. As a result, lactobacilli became central subjects in the research on host–microbe interactions, particularly in the context of immune regulation and chronic inflammatory diseases.

### 3.1. Immune Effects of Live Probiotic Bacteria

#### 3.1.1. Pro-Inflammatory Effects Under Resting Conditions

Under non-inflammatory or resting conditions, several strains of lactobacilli were reported to stimulate the production of pro-inflammatory mediators. This activity was attributed to the induction of nitric oxide synthase (iNOS) and cyclooxygenase-2 (COX2), leading to elevated levels of nitric oxide (NO) and prostaglandin E2 (PGE_2_) [29,30]. Moreover, an increased secretion of pro-inflammatory cytokines, including IL-1α, IL-1β, IL-6, interferon-γ (IFN-γ), and TNF-α, was observed for both in vitro [29,30,31,32,33] and in vivo [34,35] models following lactobacilli administration.

In human intestinal epithelial Caco-2 cells under basal conditions, certain lactobacilli were shown to increase IL-8 expression, suggesting that, even in the absence of exogenous stimuli, lactobacilli can initiate a mild pro-inflammatory response [9]. These observations supported the notion that immunostimulatory effects were not exclusive to pathological contexts and might contribute to basal immune response and mucosal vigilance.

#### 3.1.2. Anti-Inflammatory Response in Inflamed States

When administrated under inflammatory conditions, lactobacilli demonstrated immunosuppressive and anti-inflammatory properties. In epithelial models pre-treated with pro-inflammatory stimuli such as IL-1β or lipopolysaccharide (LPS), the specific strain reduced the secretion of IL-8 and NO production, suggesting a shift towards immune suppression [9].

Similar effects were confirmed in vivo. In endotoxin-challenged murine models, the administration of *Limosilactobacillus reuteri* resulted in a significant decrease in IL-1β and IL-6, along with a concomitant increase in IL-10. Additionally, a reduction in lipid peroxidant activity was observed, suggesting antioxidant activity and the restoration of redox balance in the gut [36].

In this context, the dominance of anti-inflammatory mechanisms appeared to be dependent on the inflammatory status of the host. It was reported that the immunosuppressive capacity of lactobacilli became particularly pronounced after immune activation, leading to the downregulation of pro-inflammatory cytokines and the upregulation of regulatory mediators such as IL-10 and TGF-β [37,38,39,40].

#### 3.1.3. Administration of Live Lactobacilli

Beyond their effects on cytokine profiles, lactobacilli were also shown to exert a wide range of regulatory functions on both innate and adaptive immune cells. In models of enteric infections, such as *Salmonella*-infected mice, the administration of *Lacticaseibacillus rhamnosus* resulted in an enhanced neutrophil activity, increased secretion of inflammatory cytokines, and elevated levels of pathogen-specific antibodies, ultimately reducing the intestinal bacterial burden [34].

In rIFN-γ-primed macrophages, *Latilactobacillus sakei* stimulated NO production and inflammatory cytokines, while also increasing phagocytic activity, indicating that primed innate cells retained a responsiveness to lactobacilli signals [41]. On the adaptive side, lactobacilli promoted T-cell proliferation and differentiation [29,32,42], with various strains shown to modulate T_h_1/T_h_2 balance through a shift in the T-bet/GATA-3 transcription factor ratio [35,43]. Moreover, T_reg_ populations (CD25^+^ Foxp3^+^) were expanded following lactobacilli exposure [32], and NK cell cytotoxicity was enhanced in multiple experimental systems [31].

Additional studies revealed that lactobacilli-conditioned monocytes reduced IFN-γ and IL-17 production by *Staphylococcus aureus*-stimulated T-cells and NK cells via soluble immunoregulatory mediators [44].

In preclinical models of intestinal inflammation, lactobacilli exerted protective effects on epithelial integrity and tissue morphology. Specifically, treatment with selected strains resulted in an upregulation of tight junction proteins such as mucin and occludin, supporting epithelial barrier function in inflamed colonic tissue [38]. In murine models of colitis, various lactobacilli strains significantly attenuated inflammation-induced weight loss [35,38,39], reduced colon length shortening [38,39], and also reduced histopathological alterations, including crypt loss and villus atrophy [35,38]. These have improved the disease activity index (DAI) score [38,39], which assesses body weight, stool consistency, and rectal bleeding [45].

Beyond local effects, beneficial outcomes were also observed systemically. Lactobacilli modulated neuroinflammation in endotoxin-induced models via the gut–brain axis, suggesting a role in the bidirectional communication between intestinal immunity and central nervous system homeostasis [36]. Similarly, the application of *L. salivarius* and *L. rhamnosus* strains led to a reduction in skin inflammation in mice, pointing to the relevance of probiotic action beyond the gastrointestinal tract [46]. Of particular note, *L. rhamnosus* GG (LGG) was shown to activate cytotoxic T-cells, expand tumor-infiltrating dendritic cells (DCs), and promote IFN production, highlighting its immunotherapeutic potential in a cancer model [42]. Furthermore, in immunocompromised hosts such as cyclophosphamide-treated mice, the administration of *Lactiplantibacillus plantarum* restored lymphocyte and antibody levels (IgM, IgA, and IgG) and normalized the granulocyte-to-lymphocyte ratio, which was elevated in untreated animals, suggesting immune reconstitution effects [43]. These findings positioned lactobacilli not only as anti-inflammatory agents but also as modulators of systemic immune responses in diverse disease settings.

### 3.2. Immunomodulatory Effects by Non-Viable Forms

Several studies demonstrated that non-viable lactobacilli, including heat-killed bacteria [30,33,39], cell-free supernatant (CFS) [39,40], and bacterial lysate [47,48], retained significant immunomodulatory functions. These effects suggested that viability was not a prerequisite for immune activation and, instead, pointed towards specific bacterial components as the bioactive agents.

This understanding supported the evolving concept of postbiotics, defined as “preparations of inanimate microorganisms and/or their components that confer a health benefit to the host” [20]. Among the principal bioactive entities identified are MAMPs, such as lipoteichoic acid (LTA), peptidoglycan (PGN), exopolysaccharide (EPS), and surface proteins.

These molecules were recognized by PRRs expressed on epithelial immune cells, including Toll-like receptors (TLRs) and NOD-like receptors (e.g., NOD2). Signal transduction predominantly occurred via the MyD88-dependent pathway [15,49,50], activating NF-κB and MAPKs such as ERK, JNK, and p38, concurrently contributing in the regulation of cytokine production [51,52,53], in DC activation, and in the balance of T_h_1, T_h_2, T_h_17, and T_reg_ lymphocytes [15].

In particular, a treatment with micronized and heat-treated *L. plantarum* was shown to upregulate NO and cytokine production through the TLR2/MyD88/MAPKs/NF-κB axis [30]. Similarly, a strain of *L. sakei* activated NF-κB via phosphorylation, and the degradation of its inhibitor IκBα promoted Extracellular Signal-Regulated Kinases (ERKs), c-Jun N-terminal kinase-dependent (JNK), and MAPKp38 phosphorylation in macrophages [41].

Ultimately, lactobacilli-derived MAMPs play a dominant role in modulating the immune response, directing both pro-inflammatory and anti-inflammatory pathways depending on the host status and receptor engagement. These findings offer a mechanistic rationale for the use of non-viable lactobacilli in immune-related therapeutic applications.

### 3.3. Host Determinants of the Pro-/Anti-Inflammatory “Switch”

A single MAMP can yield opposite outcomes depending on the host context. Below, the host-side determinants gate this switch and thereby reconcile pro-inflammatory effects in resting tissues with pro-resolving effects under inflamed conditions.

#### 3.3.1. PRR Abundance and Subcellular Routing

Inflammation reshapes the PRR landscape—e.g., the epithelial and myeloid upregulation of TLR2 and altered NOD2 transcription—amplifying MAMP sensitivity but also changing which pathways are available. In parallel, receptor trafficking dictates signal bias: TLR4 initiates MyD88 signaling at the plasma membrane and TRIF signaling after endocytosis [54,55]. TLR2 can also signal from endosomes to IRF7/IFN-I when sorted via TRAM [56]. In the gut epithelium, low TLR4/MD-2 and basolateral restriction further shape outputs [57]. Together, expression and routing re-weight cytokine programs.

#### 3.3.2. Pre-Activation State: Tolerance vs. Trained Immunity

Prior stimulation imprints innate cells via chromatin and metabolic rewiring. Endotoxin tolerance represses selected NF-κB target loci and limits cytokines [58], whereas trained immunity installs activating marks and glycolytic programs that potentiate responses to subsequent triggers [59]. The prevailing imprint (tolerance or training) biases whether a given MAMP dampens or boosts inflammation.

#### 3.3.3. Adaptors and PRR Crosstalk; Inflammasome Priming

Signal integration at adaptor hubs (MyD88 vs. TRIF) and PRR cooperation (e.g., Dectin-1-TLR2 [60], NOD2-TLR2 [61]) qualitatively remodel transcriptional programs beyond simple additivity. In addition, NLRP3 requires “signal-1” priming (often via TLR/NOD) before “signal-2” activation; the presence or absence of priming strongly conditions IL-1β/IL-18 outputs for the same MAMP [62].

#### 3.3.4. Endogenous Negative Regulators

Cells in inflamed milieus frequently upregulate intracellular brakes, IRAK-M/IRAK3 [63], A20/TNFAIP3 [64], SIGIRR/IL-1R8 [65], and TOLLIP [66]. These molecules limit MyD88 complex assembly, edit ubiquitin chains in NF-κB cascades, or dampen IL-1R/TLR signaling, thereby curbing cytokine amplitude even when PRRs are abundant.

#### 3.3.5. Immunometabolic Set-Point

Resting/repairing programs (OXPHOS, fatty acid oxidation) favor regulatory outputs, whereas glycolysis supports rapid pro-inflammatory effector functions [67]. Metabolites such as lactate and ROS further re-tune MAPK/NF-κB—lactate (via GPR81/YAP) suppresses NF-κB-dependent cytokines [68,69]; ROS can either activate or restrain NF-κB depending on the concentration and compartment [70]. Thus, the metabolic state steers the same MAMP toward resolution or escalation.

#### 3.3.6. Cytokine Milieu

Pre-existing IL-10 and TGF-β skew responses toward regulatory phenotypes (e.g., promotion/maintenance of Foxp3^+^ T_reg_), reducing epithelial chemokines and restraining myeloid TNF/IL-12 despite ongoing PRR engagement [71]. Tissue-derived retinoic acid cooperates with TGF-β in the gut to stabilize this regulatory bias [72].

#### 3.3.7. Cell Type-Specific Signal Decoding

The same MAMP is interpreted differently by intestinal epithelial cells (IEC) versus dendritic cells/macrophages due to distinct PRR repertoires, adaptor usage, and chromatin landscapes. IECs often prioritize barrier-protective programs (e.g., TLR2-dependent tight junction support) and limit TLR4 sensing [73], while DC subsets stratify PRRs to shape T-cell outcomes [74]; stimulus-induced “latent enhancers” in macrophages further encode history-dependent responses [75].

## 4. Lactobacilli Acts on PRR Pathways to Exert Immuno-Regulating Effects

Many studies have reported the ability of lactobacilli to determine immunomodulatory effects, directly or indirectly, by regulating PRR-dependent signaling pathways, sometimes acting even at a transcriptional level. They have been found to regulate, in particular, TLR (for instance, TLR-1/2 [30,76]; TLR4 [77]; TLR7 [78]; TLR-9 [78,79]) and NLR pathways (for instance, NLRP3 [80,81,82]; NLRC4 [82]; NOD1 [83]). However, the complexity of PRR-MAMP recognition and the signaling effectors involved are still yet to be discovered in their full extent. *Ligilactobacillus salivarius* UCC118 can stimulate TLR2 and TLR4-independent, but MyD88-dependent stimulation of cytokines (TNF-α, IL-6, IL-10, IL-12p70, IL-1β) in bone marrow-derived macrophages (BMDMs); in this case, the TLR-independent mechanism was associated with another receptor, Clec4e (Mincle), a CLR which is supposed to have functional associations with TLR2 in both humans and mice [84]. This demonstrates the great variance of PRRs whose function is regulated by probiotics, lactobacilli, and their components in particular.

### 4.1. General Overview of PRRs

The immune recognition of MAMPs was mediated by pattern recognition receptors (PRRs), expressed on the surface or within intracellular compartments of both immune (e.g., macrophages, DCs, NK cells, monocytes, neutrophils, T and B lymphocytes) and non-immune cells (e.g., epithelial cells, endothelial cells, fibroblast, smooth muscle cells) [85,86,87]. Some PRRs were even found in soluble form, circulating in biological fluids [88]. These receptors detected conserved MAMPs, which were critical from microbial surveillance and highly conserved within specific microbial groups. Five main PRR families were recognized based on structure, location, and ligand specificity: Toll-like receptors (TLRs), C-type lectin receptors (CLRs), NOD-like receptors (NLRs), RIG-I-like receptors (RLRs), and AIM2-like receptors (ALRs) [85,86,89,90]. Among these, TLRs and NLRs were the most thoroughly characterized in host–microbe interactions and are therefore discussed in greater detail below.

### 4.2. Toll-like Receptors (TLRs)

#### 4.2.1. Structure and Families

TLRs represented type I transmembrane glycoproteins composed of an extracellular leucine-rich repeat (LRR) domain for ligand recognition, a single transmembrane helix, and a cytoplasmic Toll/IL-1 receptor (TIR) domain responsible for downstream signal transduction [85,87]. The LRR motifs, repeated 19–25 times, formed a horseshoe-shaped structure that facilitated selective MAMP binding through conserved residues on the concave surface and variable residues on the convex surface [86]. TLRs recognized not only MAMPs but also damage-associated molecular patterns (DAMPs) and xenobiotic-derived molecular patterns (XAMPs) [89].

Thirteen TLRs were identified in mammals, of which ten (TLR1-10) were expressed in humans, and TLR11-13 only in mice [91,92,93]. While TLR1, TLR2, TLR4, TLR5, TLR6, and TLR10 were membrane-bound and sensed extracellular microbial structures (e.g., lipoproteins, LPS, flagellin), intracellular TLRs (TLR3, TLR7, TLR8, TLR9, TLR11-13) were localized in endosomal and lysosomal compartments and specialized in nucleic acid sensing [94].

#### 4.2.2. Cellular Distribution and Ligands

**Figure 2:**
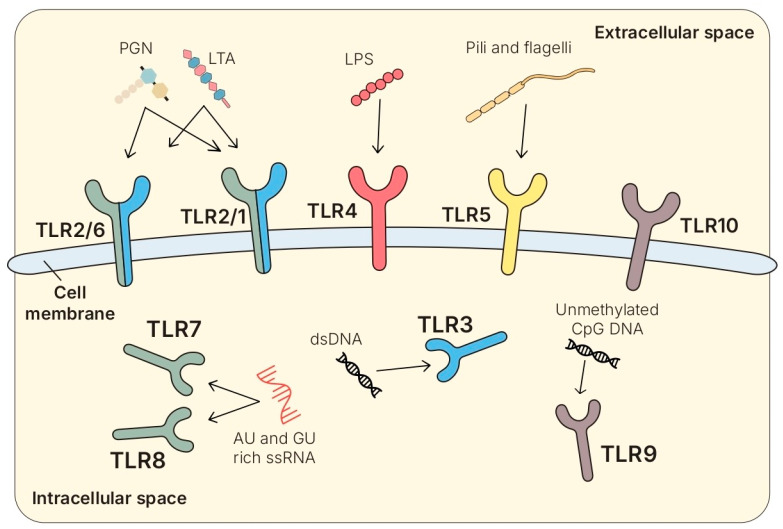
Localization and ligand specificity of human Toll-like receptors (TLRs). A comprehensive overview of TLRs expressed at the cell membrane (TLR1, TLR2, TLR4, TLR5, TLR6, TLR10) and in intracellular compartments (TLR3, TLR7, TLR8, TLR9). The figure illustrates specific microbial ligands for each TLR, such as LPS (TLR4), flagellin (TLR5), lipoproteins, peptidoglycan, and LTA (TLR2/1, 2/6), unmethylated CpG DNA (TLR9), and AU/GU-rich single-stranded RNA (TLR7/8). This spatial segregation enables the coordinated detection of extracellular versus intracellular microbial components and fine-tunes the innate immune response.

TLRs were broadly expressed in antigen-presenting cells (APC), granulocytes, lymphocytes, epithelial and endothelial cells, and tissue-resident stromal cells [88,93]. As shown in Figure 2, TLR2 formed heterodimers with TLR1 or TLR6 to expand its ligand repertoire, which included LTAs, LPPs, and PGNs [61,95,96]. TLR3 recognized dsRNA [97] and TLR4 recognized LPS from Gram-negative bacteria [93], whereas TLR5 detected flagellin [98]. TLR7 and TLR8 recognize GU-rich and AU-rich sequences on ssRNA, typically of viral origin [99], and TLR9 senses unmethylated CpG motifs in bacterial DNA [100]. Complex receptor trafficking, including endocytosis and recycling, contributed to the spatial and functional regulation of TLR activation [87,89]. Some of the TLRs were mainly expressed on the cell surface (TLR-1, -2, -4, -5, -6, -10), where they recognize foreign structural components, while others (TLR -3, -7, -8, -9- 11, -12, -13) were typically found in the endoplasmic reticulum, in endosomes, and in lysosomes [91,93].

#### 4.2.3. Signal Transduction

Upon ligand binding and dimerization, TLRs undergo conformational changes in the TIR domain, facilitating adaptor recruitment. Most TLRs used the MyD88-dependent pathway [87], except TLR3, which exclusively employed the TRIF-dependent pathway [89]. MyD88 contained a death domain (DD) that recruited IL-1 receptor-associated kinases (IRAK-1, -2, -4), leading to myddosome assembly and the downstream activation of TRAF6, TAK1, and the MAPK/NF-κB pathways [92,101]. The IKK complex (IKKα, IKKβ, and NEMO) enabled IκBα degradation and the subsequent nuclear translocation of NF-κB [102].

TRIF-dependent signaling, used by TLR3 and TLR4, activated IRF3 and type I interferon expression through TRAF3 and TBK1/IKK-ε, which regulated the expression of IFN-α and IFN-β to induce an antiviral immune response [97,103]. Some TLRs, such as TLR7-9, also trigger IRF7 activation in plasmacytoid DCs, promoting antiviral IFN-α production [104]. Negative regulators (e.g., TOLLIP, IRAK-M, and SOCS1) controlled TLR signaling by dampening cytokine production [96].

TLR engagement shapes the adaptive immune response by promoting DC maturation, antigen presentation (MHC class II, co-stimulatory molecules), and cytokine-driven T_h_ polarization [88]. TLRs thus act as critical molecular links between microbial detection and immunological memory.

### 4.3. NOD-like Receptors (NLRs)

#### 4.3.1. Structure and Classification

NLRs represent cytosolic PRRs that monitor intracellular compartments for microbial molecules and stress signals. They share a tripartite structure composed of a C-terminal LRR domain for ligand recognition, a central NACHT (NOD) domain for ATP-dependent oligomerization, and a variable N-terminal domain that defines the subfamilies: an acidic transactivation domain (AD domain) or NLRA, an NLRB or baculoviral inhibitory repeat-like domain (BIR domain), an NLRC or caspase activation and recruitment domain (CARD domain), and an NLRP or pyrin domain (PYD domain) [102]. A total of 23 NLRs were identified in humans, expressed by both immune and non-immune cells [105,106,107].

#### 4.3.2. Ligand Recognition and Inflammasome Formation

Certain NLPRs, such as NLRP1, NLRP3, NLRC4, and NAIP, formed multiprotein complexes called inflammasomes upon activation. These complexes consisted of a sensor NLR, the ASC adaptor (PYD-CARD), and pro-caspase-1, which, upon activation, cleaved pro-IL-1β and pro-IL-18 into mature cytokines [108]. Ligands included muramyl dipeptide (MDP, NOD2), iE-DAP (NOD1), flagellin (NLRC4/NAIP), LTA (NLRP6), and bacterial lipopeptides (NLRP7) [109,110]. Beyond canonical inflammasomes, some NLRs functioned as signaling hubs or negative regulators. NLRP10 and NLRP12 modulated DC activity and adaptive immunity initiation [111], while NLRP2/4 and NLRC3 downregulated NF-κB via TRAF6 inhibition [110].

#### 4.3.3. Signal Transduction and Effector Activation

NOD1 and NOD2, upon sensing iE-DAP or MDP, respectively, recruited RIPK2 via CARD-CARD interactions—RIPK2 underwent K63-linked ubiquitination and triggered the TAK1-dependent activation of NF-κB, MAPK (p38, JNK, ERK), and cytokine/chemokine expression [112,113]. These pathways facilitated early immune defense and leukocyte recruitment. Unlike TLRs, NLR activation was independent of membrane trafficking and relied solely on cytoplasmic sensing mechanisms.

TLRs and NLRs represented the principal PRRs through which the host immune system detected microbial signals. While TLRs were specialized in sensing extracellular and endosomal ligands via transmembrane signaling, NLRs monitored cytosolic perturbations and enabled rapid responses to invasive microbes. Both pathways converged on similar effectors (NF-κB, MAPKs, IRFs), yet their compartmentalized activation and ligand specificity conferred complementary roles in immune surveillance. Understanding the differential engagement of TLRs and NLRs by lactobacilli-derived MAMPs offered a deeper comprehension into probiotic immunomodulation.

## 5. MAMPs of Lactobacilli and Their Immunomodulatory Effects

Lactobacilli MAMPs can interact with PRRs, mediating multiple immunological responses. A list of key molecular patterns from different lactobacilli species and their immunomodulatory effects is presented below and also described in more detail in Table S1.

### 5.1. Peptidoglycan (PGN)

#### 5.1.1. Signal Transduction and Effector Activation

**Figure 3:**
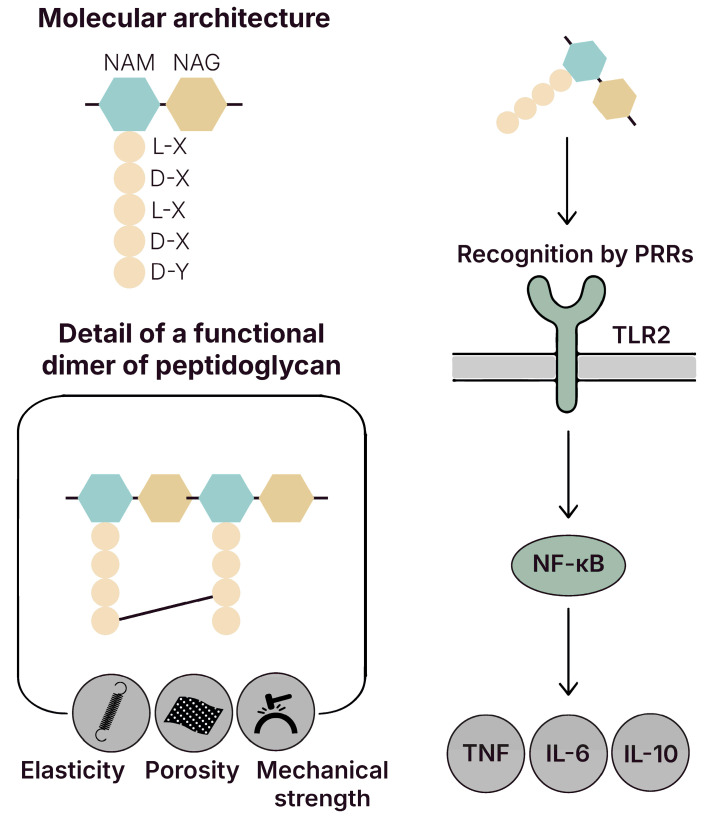
Molecular organization and immunological relevance of bacterial peptidoglycan. The figure illustrates the molecular structure of peptidoglycan (PGN), a major component of Gram-positive bacterial cell walls, composed of repeating disaccharide units of N-acetylmuramic acid (NAM) and N-acetylglucosamine (NAG) cross-linked by peptide chains (L-X, D-X, L-X, D-X, D-Y; this substituent could also be a D-Lactate). These peptide bridges confer mechanical strength, elasticity, and porosity to the bacterial envelope. PGN is sensed by pattern recognition receptors (PRRs), particularly Toll-like receptor 2 (TLR2), triggering activation of the NF-κB signaling pathway and subsequent induction of pro-inflammatory cytokines such as TNF, IL-6, and the regulatory cytokine IL-10. This interaction plays a critical role in the host’s ability to detect and respond to microbial invasion.

PGN represents a key structural element of the lactobacilli cell wall, contributing to mechanical strength, cellular morphology, and adhesion properties [114]. Alongside polyanionic teichoic acids (TAs) and surface proteins, PGN conferred elasticity, porosity, tensile strength, and electrostatic potential to the bacterial envelope, supporting ion homeostasis and the trafficking of nutrients, proteins, and antibiotics [115]. As shown in Figure 3, its structure included repeating disaccharide units of N-acetylglucosamine (GlcNAc) and N-acetylmuramic acid (MurNAc), cross-linked by short peptide chains containing both D- and L-aminoacids [114,116,117]. PGN is recognized by host PRRs [118,119], such as TLR2 and NOD2 [120,121], triggering immune signaling cascades such as NF-κB and MAPK [122]. In vivo murine model studies demonstrated that PGN derived from lactobacilli upregulated TLR2 and MAPKp38 expression in macrophages and modulated gene expression by enhancing stress response and antigen presentation markers including IL-12 and MHC II, potentially facilitating T_h_1-type immune polarization [123].

#### 5.1.2. Species- and Strain-Specific Effects

Among the best characterized strains, *L. rhamnosus* CRL1505-derived PGN suppressed HLA-DR, CD80, and CD83 expression in LPS-induced DCs while upregulating the increase simultaneously of TNF-α and IL-10 in LPS-stimulated DCs, exerting an anti-inflammatory activity. Furthermore, it increased CD86 in DCs under basal conditions [124]. It also downregulated IL-1β, IL-6, and TNF-α in LPS-stimulated RAW 264.7 macrophages [119]. Furthermore, in avian splenocytes, high doses of PGN from *L. rhamnosus* MLGA reduced IL-8 and IL-1β, even in the absence of prior stimulation; lysozyme-digested PGN fragments also upregulated β-defensin-9 in chicken peripheral blood mononuclear cells (PBMCs) and other tissues [125]. In murine models of *Streptococcus pneumoniae* infection, PGN treatment enhanced leucocyte recruitment, increased mucosal and systemic cytokines (TNF-α, IL-10, IL-6, and IL-1β), and upregulated immunoglobulin (IgA, IgG, and IgM) production. It also upregulated TLR2 and TLR-9 expression in alveolar and peritoneal macrophages, thus increasing pathogen recognition capacity [126,127]. In dual infection models with respiratory syncytial virus (RSV) and pneumococcus, PGN from CRL1505 reduced both viral load and bacterial burden [128]. In poly (I:C)-primed mice, it downregulated TLR-3 activation, suppressed IL-6, and upregulated IFN-α, -β, -γ, TNF-α, and IL-10 [129,130], suggesting a role in antiviral and anti-inflammatory defense. In this context, alveolar macrophages (AMs) played a basic role: when isolated from treated mice, they produced in vitro higher levels of IFN-γ, TNF-α, IL-6, IL-27, and chemokines such as CCL2, CXCL2, and CXCL10, enhancing immune cell recruitment [128]. Similarly, *L. paracasei*-derived PGN was also shown to significantly reduce the viral load of influenza virus in the lungs of infected mice. This antiviral effect was associated with the increased recruitment of DCs to the respiratory mucosa, paralleled by a reduction in the proportion of other immune cell subsets, suggesting a reshaping of the local immune milieu to favor antigen presentation and effective viral clearance [131].

PGN from other strains also showed immunomodulatory activity. Indeed, PGN derived from *Lactobacillus acidophilus* KLDS 1.0738 promoted regulatory T-cell responses, reducing IgE and enhancing IL-10, IFN-γ, and TGF-β secretion in β-lactoglobulin-sensitized mice [132]. In LPS-stimulated RAW264.7 cell lines, it reduced iNOS, COX2, and pro-inflammatory cytokines (IL-6, IL-1β, TN-α), highlighting anti-inflammatory properties [119,133]. Interestingly, similar effects were observed for some strains of *L. plantarum*: the PGN derived from ATCC 14917 suppressed IL-8 in poly (I:C)-stimulated HT-29 cells in vitro [47], and PGN from *L. plantarum* CAU1055 reduced levels of iNOS, COX2, and pro-inflammatory cytokines (IL-6, TNF-α) in mice models [134]. Conversely, PGN from *L. plantarum* AR113 increased the production of pro-inflammatory cytokines in RAW264.7 cells [135].

Several studies also examined PGN-derived fragments like muramyl dipeptide (MDP), a NOD2 ligand, which exerts an immunosuppressive effect by attenuating TLR4-mediated signaling [136]. In LPS-stimulated RAW 264.7 cells, MDP from *L. reuteri* inhibited IL-1β, IL-6, and CCL20 by suppressing PI3K/Akt, MAPK, and TLR4 expression [137]. A GlcNAc-MurNAc tripeptide (GMTP) from *L. acidophilus* strongly induced COX2, IL-1β, and TNF-α expression, highlighting the key immunostimulatory role of GlcNAc residues [118].

Moreover, polysaccharide–peptidoglycan complexes (PSPGs) from *L. casei* Shirota inhibited IL-6 production in lamina propria mononuclear cells via NOD2 activation and NF-κB suppression [138]; these effects were also confirmed in vivo [139]. Peptidoglycan hydrolases (PGHs), such as p40 and p75, modulated immune response by releasing PGN fragments. In DCs, Lc-p75-defective *L. casei* exhibited an impaired uptake and reduced production of TNF-α, IL-1β, and IL-6 compared to the WT [140]. A bifunctional PGH named LPH, derived from lactobacilli, attenuated TNBS-induced colitis in mice by digesting PGN and releasing NOD2 ligands, thereby increasing IL-10 and decreasing TNF-α, IFN-γ, and IL-6, exerting an anti-inflammatory activity [24].

Overall, the immunomodulatory activity of lactobacilli-derived PGN appears to be profoundly influenced by dose, structural specificity, and temporal dynamics. Dose-dependent effects have been widely reported, impacting both the amplitude and the cytokine profile of the immune response. Likewise, structural variations, such as the presence of immunogenic muropeptides like M-tri-Lys or differential D-Ala termination, confer species- and strain-specific signatures that modulate immune recognition and downstream signaling. Moreover, the kinetics of PGN-mediated activation are not uniform: early responses typically involve IL-1, IL-6, IL-12, and co-stimulatory molecules (CD80, CD86), while TNF-α expression often requires sustained stimulation [123,126,130,134,139,141]. These findings underscore the need to consider quantitative, structural, and temporal parameters when evaluating PGN-based immunomodulatory interventions.

### 5.2. Lipoteichoic Acids (LTAs)

#### 5.2.1. Structure and Key Features

**Figure 4:**
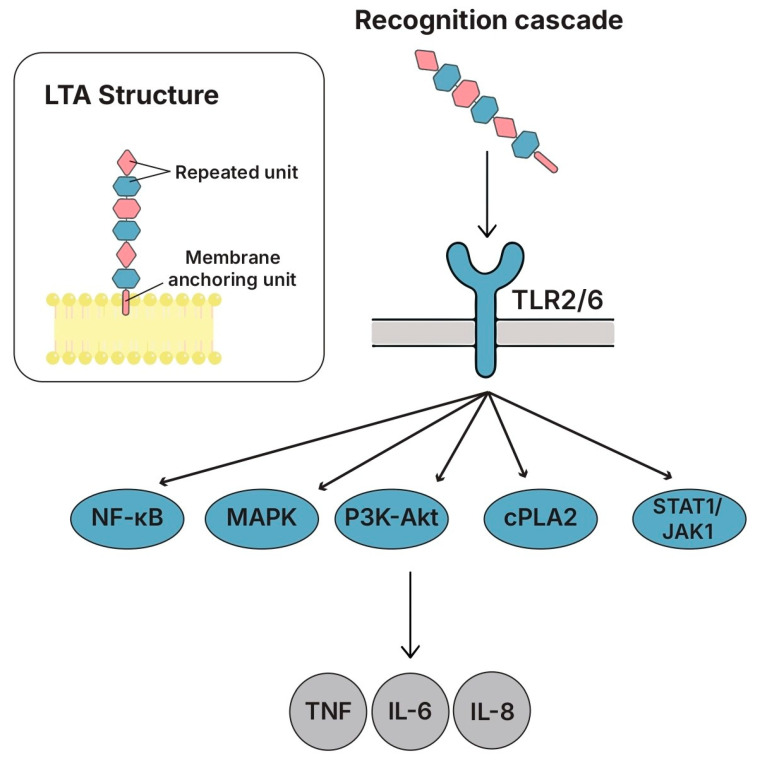
Structural features of lipoteichoic acid (LTA) and downstream immune activation pathways. The figure presents a hypothetical molecular structure of LTA, a glycolipid anchored in the cytoplasmic membrane of Gram-positive bacteria and composed of repeated units. Recognition of LTA by TLR2/6 heterodimers at the host cell surface initiates several signaling cascades, including NF-κB, MAPK, PI3K-Akt, cPLA2, and JAK1/STAT1. These cascades result in transcriptional regulation of inflammatory genes encoding TNF, IL-6, and IL-8, directing an early innate immune response to Gram-positive bacterial infection. LTA thus serves as a MAMP in the modulation of host–pathogen interactions.

LTA, a key component of Gram-positive cell walls, consists of amphiphilic molecules anchored to the cytoplasmic membrane via glycolipids and extended outward with polyglycerophosphate chains rich in D-Ala substitutions [115] (Figure 4). In lactobacilli, LTA functions as one of the major MAMPs, recognized primarily by TLR2/TLR6 heterodimers but also involving scavenger receptors such as SR-A [142]. Studies have demonstrated that LTA recognition triggered different NF-κB pathways [143,144], MAPKs [145,146,147,148], PI3K-Akt, cPLA2, and STAT1/JAK1 in macrophages and intestinal epithelial cells [147,148,149,150,151]. Some host receptors different than TLR2, which are still to be identified, may be involved in some of the LTA effects [152]. Depending on the inflammatory context, LTA exhibited dual immunomodulatory functions. The in vitro pre-treatment of macrophages or intestinal epithelium cells with LTA from various lactobacilli strains attenuated the phosphorylation of pro-inflammatory transducers (STAT-1, JAK-1, NF-κB, ERK, JNK, p38, HSP27), reduced IκBα degradation, and suppressed IL-6, IL-8, and TNF-α [148,149,150,151,153,154]. The immunological effects of LTA were strongly influenced by its structure. The acylation of LTA was necessary to induce NF-κB activation and IL-8 expression in Caco-2 cells and RAW264.7 cells, whereas deactivated LTA lost its immunostimulatory potential [144,155]. D-alanylation also played a crucial regulatory role: mutants lacking D-Ala residues in *L. plantarum* CRL1506 and WCFS1 and *L. casei* BL580 showed a lower immunomodulatory activity and altered TLR expression profiles in models of DSS (dextran sodium sulfate)-induced colitis and poly (I:C) challenge [152,156,157]. In LGG, the deprivation of D-Ala residues on LTA yielded a distinct phenotype: in the DSS-induced colitis mouse model, the modified LTA elicited stronger immunomodulatory effects than the wild-type LTA [144,158].

#### 5.2.2. Species- and Strain-Specific Effects

Several studies confirmed that LTA-mediated effects varied between species: *L. plantarum* LTA, in both diverse in vitro and in vivo models, reduced IL-8, TNF-α, IL-12, IL-4, IL-1β, and IL-6 [150,151,154,159,160,161], while enhancing IL-10 production [162]. These effects are not common in all probiotics; for example, IL-8 inhibition is typical of *L. plantarum*-derived LTA but not of *L. rhamnosus*, *Lactobacillus delbrueckii*, and *L. sakei* LTA, which showed minor effects in the same models [163]. Differences in acyl chains and D-Ala content were implicated as drivers of this specificity [142,153]. Furthermore, LTA from *L. plantarum* and from *L. casei* enhanced IL-10 while suppressing IL-12 in macrophages in vitro [145], but *L. plantarum* strain K8-LTA instead stimulated TNF-α production in PGE/LPS-primed THP-1 cells, highlighting the bidirectionality of LTA signaling [164].

*L. plantarum*-LTA reduced the expression of adhesion molecules (ICAM-1, VCAM-1, and E-selectin) in HUVEC and epithelial cells, blocked LPS-induced NO and COX2 in macrophages, and modulated cytokine responses during ETEC infection in piglets [150,159]. Furthermore, LTA from LGG restored antigen presentation and T-cell priming capacity in UVB-immunosuppressed mice, suggesting a broad immunostimulatory potential [165]. In the intestinal setting, LTA from *Lacticaseibacillus rhamnosus* GG also reprogrammed gut dendritic cells and T-cell responses, further supporting its role as a mucosal immunomodulator [166]

LTA-deficient strains such as *L. acidophilus* NCK2025 offered crucial comprehension into the immunobiology of LTA. These mutants reduced the surface expression of MHC-II, CD40, CD80, and CD86 and decreased the production of TNF-α, IL-6, and IL-12, while increasing IL-10 in a time-dependent manner [147,167,168]. In colitis models, NCK2025 treatment improved disease markers and elevated T_reg_ population, underscoring the pro-inflammatory role of WT-LTA. Similarly, *L. paracasei* LTA reduced TNF-α, IFN-γ, IL-6, and IL-1β expression, thereby improving colonic inflammation [146,169].

### 5.3. Exopolysaccharides (EPSs)

#### 5.3.1. Structure and Key Features

EPSs produced by lactobacilli are high-molecular-weight (10 to 1000 kDa) carbohydrate polymers secreted into the extracellular matrix. Depending on their association with the bacterial surface, they are classified as capsular EPSs, when tightly bound, or as slime EPSs, when loosely associated. Structurally, EPSs vary significantly in molecular weight, charge, degree of branching, and types of glycosidic linkages. This molecular heterogenicity influences both their physiochemical properties and their biological activity [170,171,172].

The immunological and antioxidant effects of EPSs depend not only on their molecular weight, but also on more refined features such as monosaccharide composition, structural conformation, and substitution patterns. While low-molecular-weight EPSs were generally more bioactive [173,174], several reports indicate that higher-molecular-weight EPSs showed enhanced immunoregulatory functions [175,176]. Acidic EPSs, in particular, were found to possess a greater antioxidant and immunostimulatory potential than neutral EPSs, likely due to an increased electron-donating ability and metal ion chelation [177,178].

**Figure 5:**
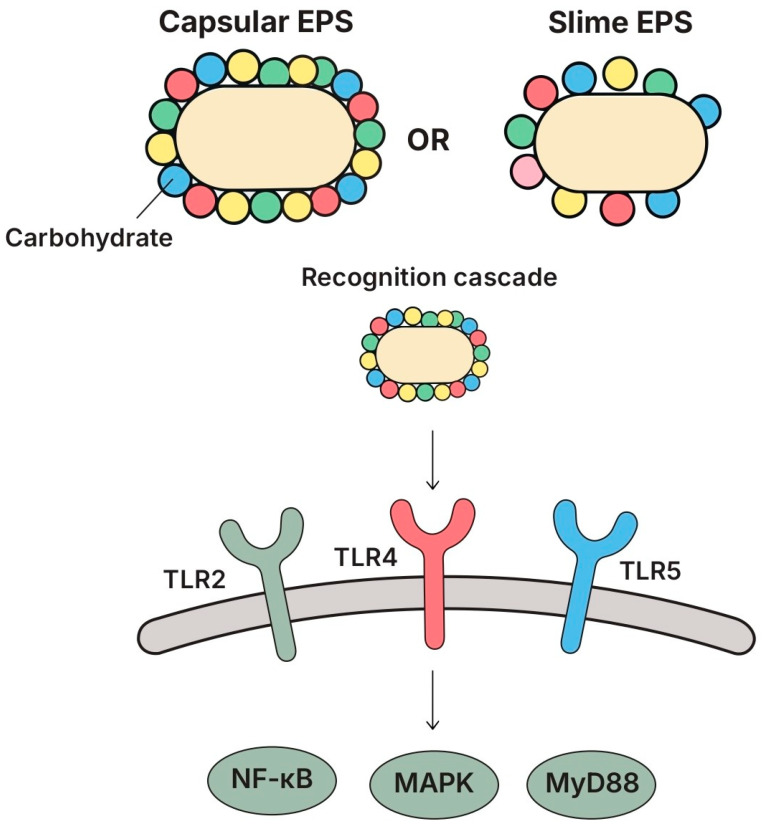
Classification of bacterial exopolysaccharides (EPSs) and their immunostimulatory potential. This scheme distinguishes two main structural types of bacterial EPSs: capsular EPSs, which form rigid external layers, and slime EPSs, which are more loosely associated with the bacterial surface. Both types are primarily composed of complex carbohydrate chains and can be recognized by host PRRs, including TLR2, TLR4, and TLR5. Upon recognition, signaling pathways involving MyD88, MAPK, and NF-κB are activated, culminating in pro-inflammatory cytokine production. EPS molecules not only provide protection to bacteria from host defenses but are also important in immune system modulation and biofilm formation.

The immunomodulatory effects of EPSs were mediated by several host signaling pathways: several studies demonstrated the involvement of NF-κB, MAPKs (ERK, JNK, p38), STAT3, MyD88, and c-Jun in EPS-induced immune responses [179,180,181]. In models using LGG-derived EPS, the phosphorylation of IκB was inhibited and the nuclear translocation of p65 was reduced, leading to decreased inflammatory signaling [182]. TLR2, TLR4, and TLR-5 were among the primary receptors affected, while MyD88 played a central role in downstream signal transduction [183,184]; these data are schematically summarized in Figure 5. In hepatic inflammation models, the IL-17/TLR2/p38/STAT axis was particularly implicated in *L. plantarum* EPS-mediated protective effects [180].

EPSs exhibited robust antioxidant properties by scavenging hydroxyl, superoxide, and DPPH radicals in a concentration-dependent manner [185,186]. In vitro assays on macrophage, enterocyte, and neuronal cells revealed that EPSs enhanced the total antioxidant capacity (T-AOC) and increased the activities of key enzymes such as superoxide dismutase (SOD), catalase (CAT), and glutathione peroxidase (GSH-Px). These effects contributed to a protection against H_2_O_2_-induced oxidative stress [187,188,189]. Furthermore, an EPS pre-treatment of epithelial cell cultures preserved tight junction proteins (claudin-1, occludin, ZO-1), counteracting the deleterious effects of TNF-α and IL-1β on epithelial barrier integrity [175].

EPSs modulated macrophage function by stimulating proliferation, enhancing phagocytosis, and increasing the production of NO, ROS, and cytokines (TNF-α, IL-1β, and IL-6, IL-10), often in a dose-dependent manner [190,191,192,193]. In HT-29 epithelial cells, EPSs induced TNF-α and IL-1β, while promoting moderate levels of IL-4 and IL-10 [194]. In models of immune stimulation (e.g., LPS-treated macrophages), EPSs showed anti-inflammatory properties by downregulating ROS production, inhibiting iNOS and COX2 activity, and reducing the secretion of pro-inflammatory cytokines [179,187]. In dermal fibroblast, EPSs reduced inflammatory mediators, suggesting potential applications in cutaneous inflammation [175].

EPSs alleviated gut inflammation in multiple murine models, including DSS-, AOM/DSS-, and pathogen-induced colitis. The treatment resulted in reduced levels of TNF-α, IL-1β, IL-6, IL-8, IFN-γ, and IL-17, while upregulating anti-inflammatory cytokines IL-10 and IL-4. Moreover, EPSs restored tight junction protein expression, ameliorated crypt damage, and also prevented colon tumor development and improved disease activity index scores [195,196,197,198,199].

#### 5.3.2. Species- and Strain-Specific Effects

In RAW264.7 macrophages, *L. rhamnosus*-derived EPSs significantly increased phagocytic activity and promoted the secretion of NO, TNF-α, IL-1β, IL-6, and IL-10 in a dose-dependent manner [200,201], whereas EPS from the KL37C strain failed to stimulate cytokine production or phagocytosis but reduced ROS levels, suggesting antioxidant effects [202].

EPSs from *L. acidophilus* induced IL-1α, CCL2, TNF-α, and PTX3 in Caco-2 cells, indicating an enhanced mucosal immune priming [203]. EPSs from *L. plantarum* promoted dendritic cell maturation by increasing MHC-II and CD86 expression, elevating IL-12p70, and suppressing IL-10, thereby skewing responses toward a Th1 phenotype [204]. By contrast, EPSs from *L. casei* did not affect NO or IL-6 in unstimulated RAW264.7 macrophages, but increased TNF-α in a dose-dependent manner; in the same model, LPS-induced EPS exposure decreased NO production, indicating a context-dependent immunomodulation [181].

The strain-dependent variability was also evident in animal models: in colitis-induced mice models, *L. plantarum*-derived EPSs exhibited strong protective effects against chemically induced colitis in mice, outperforming EPSs from *L. rhamnosus* [199]. Conversely, *L. plantarum* T10 failed to reduce IL-6 levels in colitis models, while other *L. plantarum* strains showed potent anti-inflammatory effects [198]. Nonetheless, *L. rhamnosus*-derived EPSs improved disease parameters such as body weight loss, epithelial integrity (ZO-1, occluding, claudin-1), and crypt morphology [205,206]. In infectious models, the same EPSs reduced *Salmonella typhimurium*-induced intestinal damage and decreased pro-inflammatory cytokines and IL-4 levels [182], while also exerting neuroprotective effects against oxidative injury [205] and anti-allergic properties by suppressing T_h_2 cytokines (IL-4, IL5, IL-13) and eosinophilic infiltration in sensitized mice [207]. Notably, *L. acidophilus* EPSs reduced hepatic inflammation by modulating IL-17, TGF-β, IL-10, and glutathione levels and downregulating ALT and γ-GT serum markers [208]. Moreover, EPS from *L. delbrueckii* spp. *bulgaricus* similarly induced IL-6, IL-10, IFN-γ, and NK activity both in vitro and in vivo [178]. Furthermore, *L. paracasei* mitigated colitis through MIP-2 suppression and IL-10 upregulation [209].

EPSs from *L. reuteri* L26 and DSM17938 enhanced DC activation and MHC-II/CDC80 expression and modulated IL-1β, IL-6, IL-10, IL-12p35, and TGF-β both in vivo and in vitro [184,210]. Anti-inflammatory and anti-adhesion properties were also confirmed in ETEC-infected enterocytes [211].

Notably, EPSs produced by lactobacilli also exhibit significant antioxidant properties, thereby complementing their immunomodulatory profile; for instance, EPSs derived from *L. plantarum* demonstrated a dose-dependent antioxidant activity, including an enhanced radical scavenging and upregulation of endogenous antioxidant enzymes, such as SOD and CAT [185,212]. A similar antioxidant potential was exerted also by EPSs from *L. rhamnosus* [182,205], *L. casei* [181], *L. paracasei* [213], and *L. delbrueckii* spp. *bulgaricus* [174].

### 5.4. S-Layer Proteins (SLPs)

#### 5.4.1. Structure and Key Features

**Figure 6:**
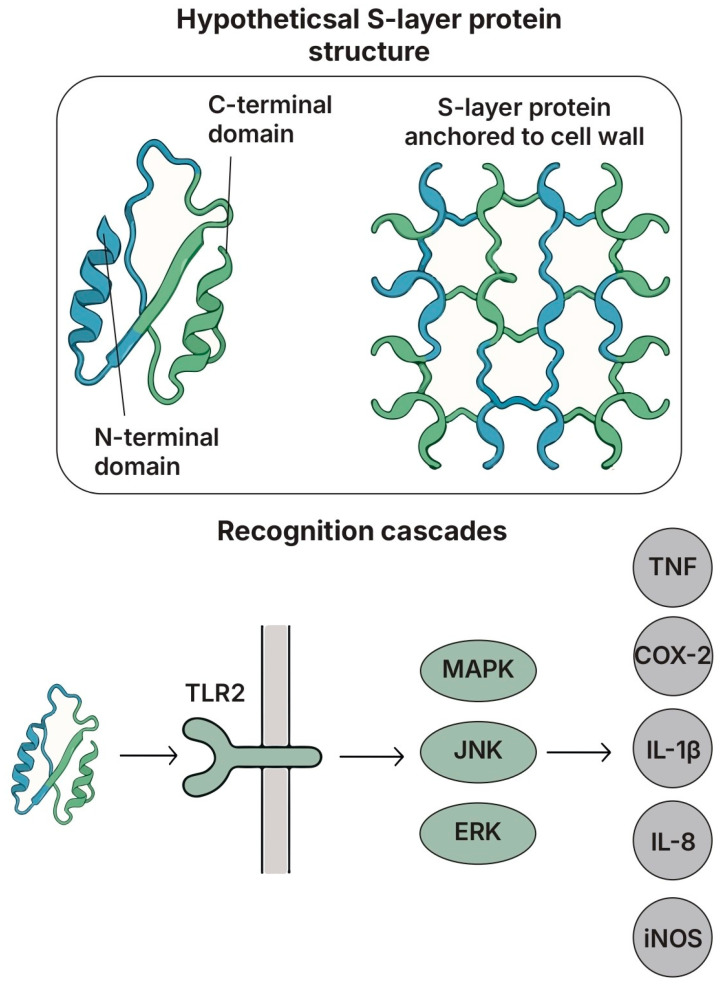
S-layer protein structure and innate immune activation. The figure proposes a model for the structure and immunological function of S-layer proteins, which are para-crystalline arrays found on the surface of many Gram-positive bacteria. These proteins exhibit a modular architecture, with conserved N- and C-terminal domains, and are anchored to the cell wall. Recognition of S-layer proteins by TLR2 on host immune cells leads to activation of Mitogen-Activated Protein Kinase (MAPK) cascades, including ERK and JNK pathways, and promotes transcription of genes involved in the inflammatory response, such as iNOS, COX2, IL-1β, IL-8, and TNF. S-layer proteins contribute to bacterial adhesion, immune evasion, and modulation of host signaling networks.

SLPs constitute the outermost proteinaceous layer of the bacteria cell envelope in many lactobacilli species. These proteins, which varied in molecular weight, structure, and post-translation modifications, formed a para-crystalline monolayer bound covalently to the cell wall polysaccharide (Figure 6) [214]. SLPs contributed to protection against environmental stressors, including acidic gastric conditions, as demonstrated by the reduced survival of *L. acidophilus* upon SLP removal [215]. Additionally, SLPs promoted bacterial adhesion and auto- and co-aggregation, supporting the microbial colonization of mucosal surfaces [216].

SLPs modulated host immunity by interacting with immune cells and influencing cytokine production in a species- and strain-dependent manner [217]. In LPS-stimulated RAW264.7 macrophages, SLPs purified from *L. acidophilus* NCFM downregulated iNOS and COX2 expression, resulting in reduced NO and prostaglandin E2 levels, while also decreasing TNF-α and IL-1β secretion [218]. In Caco-2 cells, NCFM SLPs attenuated TNF-α-induced IL-8 production and prevented the disruption of tight junction proteins ZO-1 and occludin [219].

SLP-deficient mutants of NCFM (e.g., ΔslpB, ΔslpX and ΔslpB) displayed altered immunostimulatory profiles. A co-culture with bone marrow DCs revealed a reduced expression of MHC-II, CD11b, and cytokines such as IL-6, MIP-1α, and IL12p40 compared to WT strains [220]. These findings were consistent with earlier studies using engineered strains such as NCK1377-Cl (SlpA-knockout/SlpB-dominant), which elicited higher IL-12p70, TNF-α, and IL-1β but reduced IL-10 levels compared to WT NCFM. Otherwise, IL-6 was strongly prompted by the WT strain compared to the mutant [221]. Other studies further confirmed that mutants lacking SLPs induced weaker T_h_1 cytokine responses and promoted IL-10 production in DCs [222,223].

#### 5.4.2. Species- and Specific-Strain Effects

Several SLPs engaged PRRs to initiate immune signaling. SlpA from *L. acidophilus* bound to DC-SIGN on DCs, regulating both pro- and anti-inflammatory cytokine production. In the presence of LPS, SlpA promoted a T_h_2-skewed response through IL-4 production, although SlpA alone was insufficient to stimulate naïve CD4^+^ T-cell proliferation [221,224]. SLPs also modulated MAPK pathways: SLPs from *L. acidophilus* ATCC4356 reduced ERK1/2, JNK, and p38 phosphorylation in Caco-2 cells challenged with *Salmonella typhimurium*, while simultaneously downregulating IL-8 secretion and protecting tight junction integrity [225,226].

SLPs from *L. acidophilus* ATCC4356 induced CD80 and CD86 expression in DCs and promoted IL-10 secretion, while decreasing IFN-γ and TNF-α production. They also enhanced interferon-stimulated gene (ISG) expression, suggesting a role in antiviral defense [227]. In macrophages, *L. acidophilus* CICC6074 SLPs increased phagocytic activity and NO production in a dose-dependent manner [215]. Similar immunostimulatory effects were observed for SLPs from *L. helveticus* LH2171, which upregulated hBD expression in Caco-2 cells via TLR2 and JNK activation. Other species, including *Limosilactobacillus amylovorus*, *Lentilactobacillus buchneri*, and *Levilactobacillus brevis* demonstrated a comparable but strain-specific JNK pathway activation [228].

SLPs from LGG decreased TNF-α and IL-6 production in RAW264.7 macrophages and inhibited ERK phosphorylation following LPS challenge [229]. In IPEC-J2 epithelial cells, LGG-derived SLPs and EPSs reduced the transcription of pro-inflammatory cytokines (IL-6, IL-12, and TNF-α) [230]. SLPs from *L. paracasei* spp. *paracasei* M5-L and *L. casei* Q8 attenuated pathogen-induced reductions in ZO-1 and occludin in the gut epithelium, protecting epithelial integrity [231,232]. Similarly, *L. plantarum* SLPs alleviated IL-8 and TNF-α responses in inflamed Caco-2 cells and restored tight junction expression by modulating LPS-induced miRNA dysregulation [233].

A domain of an SLP, termed the micro integral membrane protein (MIMP), isolated from *L. plantarum* CGMCC1258, significantly reduced inflammation in DSS-induced colitis mice by decreasing IFN-γ, IL-17, and IL-23 while increasing IL-4 and IL-10, and was also found to improve the health status of these mice, while at the same time reducing IFN-γ, IL-17, and IL-23 levels and up-expressing IL-4 and IL-10 levels compared to the mice who did not receive MIMP treatment [234]. These anti-inflammatory effects were also confirmed by a PBMCs/Caco-2 cell co-culture model in vitro challenged with LPS, where it has been established that MIMPs interfere with the TLR4 pathway and with NF-κB, MAPK, and JNK. Also, important effects on histone acetylation were discovered, and it was reported that MIMPs can inhibit LPS-induced histone acetylation, modulating cell transcription at an epigenetic level [235].

Despite the strong evidence for strain-specific activity and pathway modulation, the complete spectrum of SLP–host receptor interactions remained incompletely defined. Further research is needed to elucidate the structure–function relationship of SLPs and to explore their therapeutic applications in inflammation and mucosal immunity.

### 5.5. Pili

#### 5.5.1. Structure and Key Features

**Figure 7:**
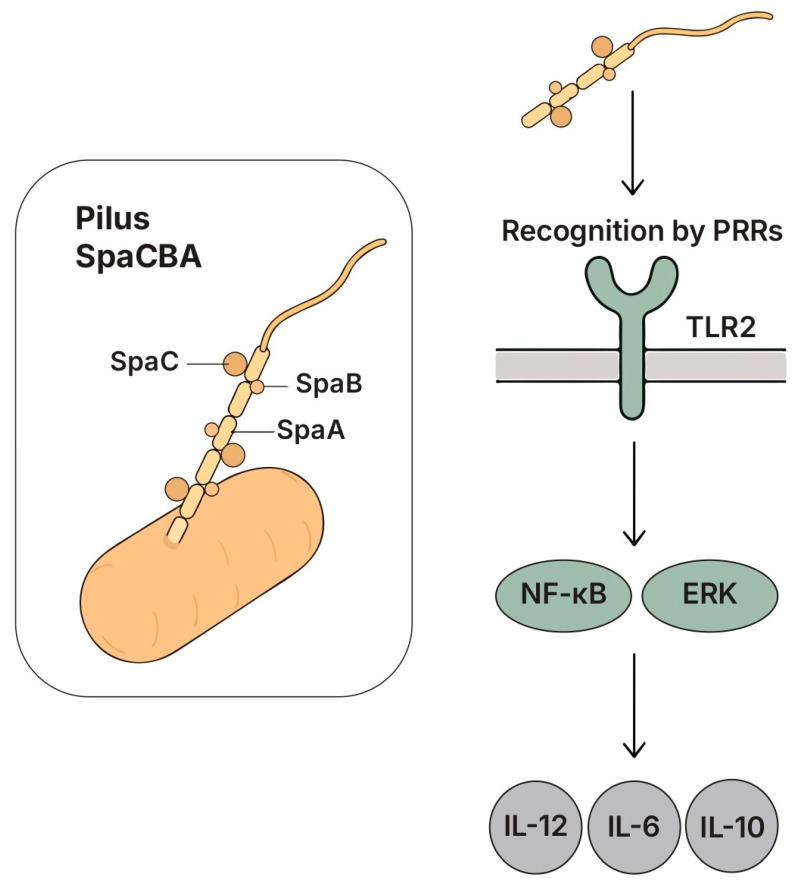
Structure and immunological interaction of the SpaCBA pilus in Gram-positive bacteria. The SpaCBA pilus is a well-characterized sortase-dependent pilus system comprising SpaA (major shaft subunit), SpaB (basal subunit), and SpaC (adhesive tip subunit). This figure highlights the hierarchical organization of pilin subunits and their recognition by TLR2 on host immune cells. SpaCBA engagement triggers the activation of NF-κB and ERK pathways, resulting in the induction of both pro-inflammatory (IL-6, IL-12) and anti-inflammatory (IL-10) cytokines. These pili facilitate adhesion to mucosal surfaces and contribute to bacterial persistence while modulating mucosal immune responses.

Pili or fimbriae represent long filamentous structures protruding from the bacterial cell wall, composed of polymerized pilin subunits arranged via a sortase-dependent mechanism [236]. These structures facilitated adhesion to host tissues, interbacterial aggregation, and environmental sensing. Among lactobacilli, the SpaCBA pilus system of LGG was the most characterized [237,238] (Figure 7).

#### 5.5.2. Species- and Strain-Specific Effect

The SpaCBA pilus, and particularly the SpaC adhesin loaded at its tip, mediated high-affinity binding to intestinal mucins via β-galactoside-containing carbohydrate motifs [239,240,241]. This adhesion promoted the colonization of intestinal epithelial cells and mucus layers and was essential for SpaC-dependent immunological signaling. The deletion of SpaC impaired mucin binding and reduced LGG’s ability to colonize the intestinal surface [240].

SpaCBA pili also functioned as immunomodulatory structures: in human monocyte-derived DCs, purified SpaCBA pili activated TLR2-dependent NF-κB signaling and triggered cytokine production, including IL-6, IL-10, and IL-12 [240,242]. Interaction with the DC-SIGN receptor on immature DCs further supported SpaCBA-mediated immune engagement, inducing IL-6, IL-10, IL-12p40, and IL-12p35. In SpaCBA-deficient LGG mutants, Caco-2 cells exhibited an increased IL-8 expression, and macrophages showed decreased IL-10 and elevated IL-6 production, confirming the role of SpaCBA in balancing pro- and anti-inflammatory responses [239,243].

SpaCBA-expressing LGG enhanced ERK phosphorylation and ROS generation in epithelial cells, promoting cell proliferation and exhibiting radioprotective effects both in vitro and in vivo; these functions were absent in SpaC-deficient mutants [244]. Moreover, SpaC contributed to the competitive exclusion of *Enterococcus faecium* by preventing its adhesion to intestinal mucus, indicating a potential role in microbiota modulation and pathogen control [245].

The SpaCBA pilus system of LGG exemplified a multifunctional bacterial appendage that combined adhesive and immunological functions. Through the direct engagement of epithelial and immune cells, SpaCBA pili modulated host responses, reinforced mucosal integrity, and potentially contributed to host defense against pathogens. Future studies should explore additional pili/fimbriae in lactobacilli to determine whether similar dual-function MAMP systems are widespread across probiotic species.

### 5.6. Lipoproteins (LPPs)

#### 5.6.1. Structure and Key Features

Bacterial LPPs are post-translationally modified proteins covalently anchored to the cytoplasmic membrane through lipid moieties. In Gram-positive bacteria, including lactobacilli, these lipoproteins are surface-exposed and accessible to host immune sensors [246,247]. Their immunological relevance stems from their capacity to act as MAMPs, particularly through the engagement of TLR2. Indeed, TLR2 forms heterodimers with either TLR1 or TLR6, allowing for the recognition of tri- and di-acetylated LPPs, respectively [248,249]. Despite this well-established signaling paradigm, LPPs from probiotic lactobacilli remain relatively underexplored in terms of molecular specificity, structure–activity relationships, and PRR targeting beyond TLR2.

#### 5.6.2. Species- and Strain-Specific Effects

Experimental studies suggest that the immunological role of lactobacilli-derived LPPs may vary considerably in strain and host contexts. In human PBMCs, an LPP-deficient mutant of *L. plantarum* triggered the enhanced production of IL-12, TNF-α, IL-1β, and IL-8, and reduced the levels of IL-10 compared to the WT strain, suggesting that intact LPPs may help dampen host inflammatory responses [250]. In contrast, purified LPPs from *L. plantarum*, *L. casei*, or LGG failed to attenuate flagellin-induced IL-8 secretion in PBMCs, indicating a limited immunosuppressive activity in those settings [251].

Nevertheless, other experimental systems have yielded different outcomes. In human myometrial hTERT-HM cells, LPP-enriched fractions isolated from the CFS of *L. rhamnosus* GR-1 significantly reduced the LPS-induced secretion of IL-6, IL-8, and MCP-1, pointing toward a potential anti-inflammatory role in epithelial or barrier tissue contexts [252]. These apparently divergent findings highlight the species- and tissue-dependent complexity of LPP–immune system interactions. Further studies are needed to clarify whether structural differences among LPPs, such as lipid composition, acylation patterns, or protein domains, are responsible for these distinct immunological outcomes.

### 5.7. DNA and CpG-Rich Oligodeoxynucleotides

#### 5.7.1. Structure and Key Features

**Figure 8:**
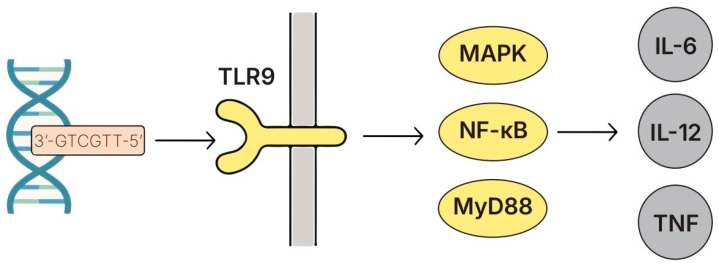
TLR9-mediated recognition of bacterial DNA and resulting immune activation. Bacterial genomic DNA, characterized by unmethylated CpG motifs such as 3′-GTCGTT-5′, is detected by TLR9 in endosomal compartments of immune cells. This figure outlines the molecular events following CpG recognition, which include recruitment of the adaptor protein MyD88, activation of NF-κB and MAPK signaling pathways, and the induction of cytokines such as IL-6, IL-12, and TNF. These responses are essential for initiating adaptive immune responses against intracellular bacterial pathogens and for driving T_h_1 polarization.

Bacterial genomic DNA, particularly from lactobacilli, contained immunologically active unmethylated cytosine–phosphate–guanine (CpG) motifs that were recognized by TLR9 located in the endosomal compartments of immune cells (Figure 8). TLR9 detection initiated MyD88-dependent signaling cascades that led to NF-κB and MAPK activation and influenced both innate and adaptive immunity [94,253]. These CpG-rich sequences were abundant in bacterial DNA and represented key MAMPs capable of modulating immune responses in a context- and sequence-dependent manner.

#### 5.7.2. Species- and Strain-Specific Effects

Probiotic-derived genomic DNA (gDNA) exhibited both immunostimulatory and anti-inflammatory properties. DNA from the VSL#3 probiotic formulation induced IL-6 and IL-12p40 secretion in bone marrow-derived macrophages (BMDMs) by activating NF-κB and JNK pathways and protected mice against DSS-induced colitis through a TLR9-MyD88-dependent mechanism [254]. Similarly, gDNA from LGG reduced inflammatory cytokines in LPS-challenged RAW264.7 macrophages and suppressed p38 and NF-κB activation, especially when co-administrated with SLPs [229].

Defined synthetic oligodeoxynucleotides (ODNs) containing CpG motifs derived from lactobacilli DNA, such as ID35 from LGG, activated DCs and promoted T_h_1-biased immune responses in ovalbumin-challenged mice. These responses were accompanied by reduced T_h_2-type cytokines and a decreased IgE production, indicating anti-allergic activity [255]. CpG-ODNs also synergized with TLR4 ligands to amplify IL-12 and TNF-α expression, while increasing TLR9 mRNA and p65 phosphorylation [230]. Characteristic motifs such as 3′-GTCGTT-5′ and 3′-PuPuCGPyPy-5′ were detected in *L. plantarum*, *L. rhamnosus*, *L. casei*, and *L. delbrueckii* [256].

Not all CpG motifs triggered immune activation; some acted suppressively: an example was ODN 7F, a CpG-rich sequence from *L. casei* DNA, which activated TLR9 but reduced pro-inflammatory mediators including MIP-2, iNOS, and COX2 in THP-1 macrophage-like cells, and was confirmed against DSS-induced colitis in mice [257]. Immunosuppressive ODNs from *L. paracasei* reduced IL-6, TNF-α, and IL-12p40 in DCs and promoted T_reg_ differentiation in vivo, contributing to mucosal tolerance [258]. Similar anti-inflammatory effects were reported for ODNs derived from LGG in DSS-induced colitis in mice models [259].

The immunological effects of CpG-rich DNA were species- and host-dependent. DNA from *L. rhamnosus* and *L. fermentum* decreased TNF-α secretion in endotoxin-stimulated RAW264.7 cells in a dose-dependent way [260]. However, the same CpG-rich sequences could exert divergent outcomes depending on the host immune context: DNA from *L. plantarum* reduced NF-κB and STAT1 signaling in colonic biopsies from ulcerative colitis patients, while in Crohn’s disease biopsies it promoted IL-17 expression, indicating a differential recognition or downstream signaling by inflamed tissue [261].

Lactobacilli-derived DNA and CpG-rich ODNs represented potent immunomodulators capable of inducing either inflammatory or regulatory immune responses through TLR9 signaling [257]. Sequence-specific and species-dependent effects highlighted the need for precision in characterizing functional motifs and host–pathogen interactions. Elucidating the structural determinants of immunostimulatory versus suppressive CpG-ODNs remains essential for their therapeutic exploitation in inflammatory and allergic diseases.

### 5.8. Membrane Vesicles (MVs)

#### 5.8.1. Structure and Key Features

**Figure 9:**
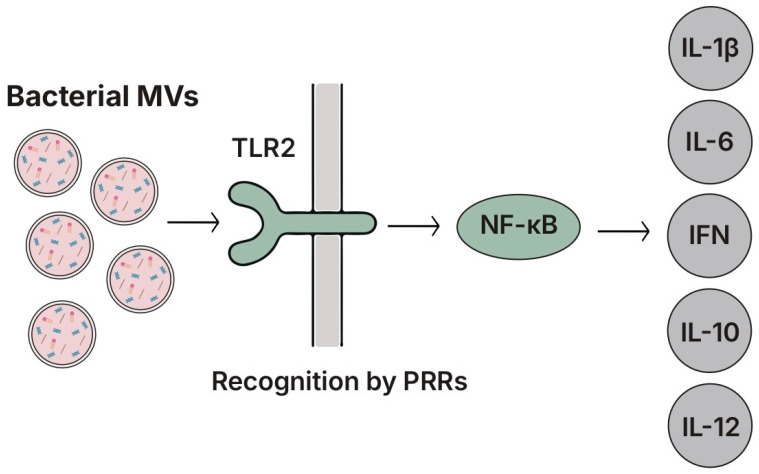
Bacterial membrane vesicles (MVs) as immunomodulatory structures. Depiction of bacterial MVs, which are spherical structures composed of lipid bilayers and enriched in MAMPs. MVs are recognized by host TLR2 and activate NF-κB signaling, leading to the production of interferons and cytokines such as IL-6, IL-1β, IL-10, and IL-12. These vesicles facilitate inter-kingdom communication, contribute to immune modulation, and may act as delivery systems for bioactive compounds during host–microbe interactions.

MVs constitute spherical, bilayered nanostructures (20–400 nm) naturally released by several lactobacilli [262] (Figure 9). These vesicles enclose cytoplasmic and membrane-derived components, including nucleic acids, proteins, LTA, and PGN fragments, which are implicated in host–microbiome immune interactions [40,263,264]. The internalization of MVs by epithelial and immune cells is required to initiate most immunological effects, as demonstrated in both in vitro and in vivo settings [45,265].

#### 5.8.2. Species- and Strain-Specific Effects

MVs derived from *L. sakei* and *L. plantarum* stimulated IL-6 and IgA production in Peyer’s patches cells via the activation of DCs [266,267]. These responses were shown to be dependent on TLR2 engagement [268]. MVs from *L. plantarum* JCM8341 further activated NF-κB signaling and induced IL-1β, IL-6, IL-10, IFN-γ, and IL-12 in RAW264.7 macrophages, promoting the T_h_1-skewing of naïve T-cells [267]. In HT-29 epithelial cells, the same MVs suppressed LPS-induced IL-8 expression in a dose-dependent manner, while in DSS-induced colitis models they reduced in a concentration-dependent manner. These MVs also alleviated DSS-induced colitis models; they reduced weight loss and tissue damage. These effects were partially mediated by MV-associated small RNAs capable of inhibiting TLR4-dependent pathways [45].

MVs from *L. rhamnosus* JB-1 induced IL-10 secretion in BMDCs and suppressed TNF-α-induced IL-8 expression in intestinal epithelial cells, likely through their LTA content [269]. Vesicles from *L. reuteri* enhanced IL-6 and IL-10 production in resting PBMCs and downregulated IFN-γ and IL-17 in *S. aureus*-stimulated PBMCs [40]. In related experiments, only *L. reuteri*-derived but not LGG-derived MVs attenuated TNF-α and IFN-γ expression, possibly due to strain-specific enzymatic cargo, including 5′-nucleotidase [264].

The immunological effects of MVs were highly variable among strains. *L. reuteri*-derived MVs suppressed intestinal inflammation and oxidative stress in LPS-treated broilers, inhibited pro-inflammatory cytokines (TNF-α, IL-6, IL-7), and enhanced regulatory mediators such as IL-10 and TGF-β. However, MVs downregulated IFN-γ and IL-17 expression in co-cultured splenic lymphocytes while promoting CD25^+^ and CTLS-4, facilitating T_reg_ polarization. Vesicle-associated nucleic acids and membrane proteins were considered critical mediators of these effects [263].

Strain specificity also depended on environmental factors. For instance, *L. casei* MVs produced under agitation and *L. plantarum* MVs generated at a low pH induced greater IL-10 and reduced TNF-α in THP-1 macrophages compared to standard conditions, despite lower vesicle yields [270]. This observation suggested that vesicle bioactivity was sensitive to both genetic and environmental inputs.

All these studies point to different factors responsible for the immunomodulatory effects; the structure complexity of the lactobacilli MVs, as a matter of fact, allows us to focus our attention on multiple MAMPs and molecules, exposed on the membrane surface or localized inside the vesicles, which all probably have a role in influencing the host immune system. Also, we can assert that MV properties are strongly species- and strain-dependent, but also are influenced by the culture conditions the strains have been exposed to.

### 5.9. Immunobiotic-like Particles (IBLPs)

IBLPs, also referred to as bacterial-like particles (BLPs), consist of hollow PGN-based scaffolds mimicking MAMP-rich vesicles. These particles demonstrate a robust immunostimulatory activity in vivo [271]. *L. rhamnosus*-derived IBLPs, when used as a vaccine adjuvant, enhanced Rotavirus-specific IgA and IgG responses in mice and increased the production of TNF-α, IFN-γ, and IL-4 in mucosal and systemic compartments [272]. Other formulations, such as BLP23017, significantly boosted IL-1β, IL-6, IL-12, TNF-α, and IFN-γ secretion in macrophages and elevated mucosal sIgA and serum antibody levels in immunized animals against *Clostridium perfringens* [273].

Both membrane vesicles and IBLPs represent complex, multifactorial delivery system for MAMPs. Their immunomodulatory activity depends on molecular cargo, surface-exposed ligands, and host–cell recognition mechanisms. As strain-specific and environmentally responsive platforms, MVs and IBLPs show strong potential for immunotherapeutic and adjuvant applications, although further mechanistic studies are needed to elucidate their receptor-binding profiles and optimize their functional design.

## 6. How Do Lactobacillaceae MAMPs Differ from Those of Other Bacteria?

Many Gram-positive MAMPs converge on TLR2/NOD2, yet lactobacilli display recurring chemo-structural signatures (e.g., LTA D-alanylation status, specific EPS chemotypes, S-layer/pilus architectures) that bias PRR usage, kinetics, and functional outputs distinct from other commensals or pathogens. The table below (Table 1) highlights reproducible differences with functional implications.

Together, these features support a “lactobacilli MAMP signature”: tunable LTA acylation/D-alanylation, EPS chemotypes with barrier-repair functions, S-layer/DC-SIGN engagement, and sortase-piliated adhesion. While TLR2/NOD2 convergence is shared across Gram-positives, these structural/topological traits bias lactobacillus outputs toward epithelial protection, restrained NF-κB, and regulatory T-cell support more consistently than in many other commensals or pathogens.

## 7. Main Challenges in Understanding Immunomodulation by Lactobacilli

Despite substantial advances in the comprehension of how discrete MAMPs such as PGN, LTA, and EPS engage host immune mechanisms, several critical challenges continue to limit our full understanding of lactobacilli-derived immunomodulation.

A first major limitation resides in the discrepancy between in vitro and in vivo models. While in vitro systems enable a high-resolution analysis of cellular pathways, they often fail to recapitulate the complexity of host physiology, including microbial community interactions, mucosal architecture, and immune cell crosstalk. Conversely, animal models, though more physiologically relevant, exhibit an interspecies variability in immune signaling and microbiome composition, complicating extrapolation to human systems [283,284].

Another key issue lies in the strain-specific nature of MAMP bioactivity. Closely related strains can exhibit markedly different immunomodulatory profiles due to the subtle genomic or regulatory variations affecting MAMP abundance, structure, or release; this high degree of functional heterogeneity presents a challenge for reproducibility and clinical applications [285].

Furthermore, most studies have characterized complex molecular landscapes in which multiple MAMPs act simultaneously. These combinations may generate additive, synergistic, or even antagonistic effects on immune signaling, a dimension that remains largely unexplored and difficult to prove experimentally [286].

Host-specific factors further complicate interpretation. Immune responsiveness to bacterial signals is shaped by host genetics, existing microbiota composition, diet, age, and disease state [287]. Interindividual variability may obscure the generalizability of MAMP-based interventions unless studies account for such biological contexts.

Lastly, regulatory and technological barriers limit the translational potential of MAMPs. Issues surrounding the purity, reproducibility, and scalability of MAMP preparations, as well as the definition of pharmacologically active doses, must be rigorously addressed to meet clinical-grade manufacturing and safety standards.

To overcome these limits, future studies should integrate high-throughput -omics approaches, ex vivo immune profiling, and personalized host–microbiome interaction models. Such integrative strategies are essential to fully harness the therapeutic potential of lactobacilli-derived MAMPs.

## 8. Discussion

Lactobacilli are among the most frequently consumed probiotics, thanks to their beneficial effects on human health. For instance, fermented dairy products containing live and active cultures have been associated with a reduced risk of type II diabetes, better glycemic control [288], reduced weight gain [289], and lower circulating triglyceride levels, systolic blood pressure, and insulin resistance [290]. Various lactobacilli species also appear to play important roles in both the treatment and prevention of antibiotic-associated diarrhea [291], as well as in reducing the incidence or severity of gastrointestinal infections by *Salmonella* spp. [292,293], *Clostridium difficile* [291,294], and *H. pylori* infection [295,296]. Moreover, current research is exploring extra-intestinal applications of lactobacilli for neuronal autoimmune and neurodegenerative diseases, including multiple sclerosis [297], Alzheimer’s disease [298,299], Parkinson’s disease [300], and neurodevelopmental disorders such as ADHD [301].

One of the key reasons for this broad therapeutic potential is that lactobacilli possess various MAMPs. These MAMPs can be recognized by PRRs such as TLRs and NLRs, triggering complex signaling pathways that modulate cellular behavior at the transcriptional level and thus influence immune responses. The net effect can be either pro-inflammatory or anti-inflammatory, depending on the lactobacilli species or strain [123,141,163], the specific MAMP in question, the target cell type, and the activation status of that cell.

A clear example is seen in macrophages: pro-inflammatory (M1) macrophages are typically induced by Th1 cytokines (e.g., IFN-γ, TNF-α) or bacterial LPS and secrete high levels of pro-inflammatory cytokines, whereas anti-inflammatory (M2) macrophages, stimulated by Th2 cytokines, produce cytokines that dampen inflammation [302,303]. Many in vitro studies demonstrate that lactobacilli MAMPs can induce pro-inflammatory effects in resting (unstimulated) macrophages [193], while in LPS-primed macrophages they actually suppress excessive pro-inflammatory cytokine production [199]. The capacity of lactobacilli and their MAMPs to shape immune responses makes them appealing for the prevention and treatment of numerous immune-related disorders. For IBDs, which include CD and UC, lactobacilli have displayed protective activities by inducing mucin-2 and occludin, increasing anti-inflammatory mediators, and reducing IL-6, IL-1β, and TNF-α levels, along with diminishing the presence of CD25^+^ T-cells in inflamed tissues [38,304,305]. Polysaccharide–peptidoglycan complexes from *L. casei* Shirota significantly inhibited IL-6 production in LPS-stimulated colonic lamina propria mononuclear cells, RAW264.7 macrophages, and PBMCs from UC patients, leading to milder symptoms in ileitis mouse models [139]. Peptidoglycan from *L. salivarius* Ls33 was shown to protect BALB/c mice against colitis through NOD2 receptor activation, resulting in elevated colonic IL-10 levels and increased T_reg_ cells in the mesenteric lymph nodes, even with oral administration [141]. LTA has also been studied for its anti-inflammatory effects in colitis models; numerous findings suggest its potential use in alternative IBD therapies [153,161,306]. In addition, EVs derived from lactobacilli can ameliorate colonic inflammation in murine models. For example, EVs from *L. plantarum* Q7 reduced pro-inflammatory cytokines in the colon and serum of DSS-induced colitis mice, potentially through modulating the TLR4/MyD88/NF-κB pathway [307]. Beyond the gut, lactobacilli-derived MAMPs have been examined for their effects on other inflammatory conditions. LTA purified from *L. plantarum* effectively suppressed atherosclerosis, reducing monocyte/macrophage infiltration and the pro-inflammatory response in the aortic sinus of mice, thus mitigating plaque degradation [150]. LTA from LGG showed radioprotective effects in the mouse intestine, acting via TLR2- and COX2-dependent mechanisms in mesenchymal stem cells and promoting epithelial cell migration—an effect that may hold promise for mitigating radiotherapy-induced damage [308]. Moreover, purified EPSs from *L. rhamnosus* KL37 demonstrated immunosuppressive properties in a collagen-induced arthritis model, decreasing serum autoantibody levels and ameliorating disease symptoms by modulating T-cell-dependent humoral responses [309]. Allergy treatment is another area where lactobacilli MAMPs display significant potential. *L. rhamnosus* GG-derived immunostimulatory oligodeoxynucleotides and *L. acidophilus* PGN have both been shown to reduce IgE levels in mouse models [255]. Likewise, EPSs from *L. paracasei* were able to reduce IgE and IL-4 levels in serum and ear tissue in a murine model of contact dermatitis, improving dermal health [310]. Furthermore, some MAMPs from lactobacilli enhance antitumor immunity. An EPS from lactobacilli strains has been reported to increase CCR6+ CD8^+^ T-cell populations in Peyer’s patches, supporting their migration to CCL20-expressing tumor sites and thereby boosting the effects of immune checkpoint blockades (e.g., anti-CTLA-4 mAb) [311]. Similarly, EVs from LGG ATCC 53103 synergized with anti-PD-1 immunotherapy, fostering a higher infiltration of MHC II+ dendritic cells, CD8^+^, and CD4^+^ T-cells into the tumor [312]. The oral administration of *L. reuteri* also promoted antitumor immunity, enhancing responsiveness to checkpoint inhibitors via the production of indole-3-aldehyde in tumor tissue, which supported stronger CD8^+^ T-cell activity [313]. Ongoing research covers an even broader spectrum, including diabetes [314,315], protection against ethanol-induced liver damage [316], and neurodegenerative pathologies [317]. Despite these encouraging data, the precise mechanisms underlying lactobacilli-mediated immunomodulation are not fully elucidated, and many studies still rely on whole-cell effects. Nevertheless, the mounting evidence that isolated MAMPs can regulate immunity in a myriad of contexts has spurred interest in developing them as “postbiotic” solutions, i.e., non-viable microbial products with retained bioactivity. Despite the growing body of preclinical evidence, clinical validation of these effects remains limited. To date, only one clinical trial has explored the therapeutic efficacy of a postbiotic MAMP preparation (derived from *Streptococcus thermophilus* rather than lactobacilli), highlighting the early stage of translation in this field [318]. As their individual and synergistic roles become better understood, lactobacilli-derived MAMPs are emerging as versatile tools for the management of inflammatory and immune-related conditions.

## 9. Conclusions

The growing body of evidence on lactobacilli-derived MAMPs underscores their essential immunomodulatory role. Acting through TLR, NLR, and other signaling pathways, these molecules can drive pro- or anti-inflammatory responses and often remain active in non-viable (postbiotic) formats, expanding their therapeutic scope even for immunocompromised populations.

In this review, it has been shown how specific MAMPs—such as PNG, LTA, EPS, SLPs, lipoproteins, and CpG ODNs—can strengthen epithelial barriers, promote T_regs_, mitigate excess inflammation, or boost pathogen defense. Beyond gut-focused benefits, these immunomodulatory activities extend into areas such as allergy management, autoimmune support, and tumor immunotherapy, indicating a wide range of clinical potential.

Looking ahead, several hurdles remain. Bridging in vitro–in vivo data, clarifying strain-dependent MAMP differences, and optimizing postbiotic formulations are all essential. Still, harnessing lactobacilli MAMPs as targeted immunomodulators remains a promising approach, aligning traditional probiotic usage with next-generation therapies for inflammatory, infectious, and neoplastic diseases.

Looking ahead, the following developments can be anticipated: (a) Formulating stable postbiotic products enriched for specific MAMPs: Methods to isolate and concentrate LTA, EPS, or specialized vesicles are crucial for scaling these therapies. Stabilization protocols, freeze-drying technology, and microencapsulation may enhance shelf-life and targeted delivery. (b) Conducting more human clinical trials: While a solid base of animal data exists, well-controlled human studies are needed to confirm the optimal dosing, safety in immunocompromised subsets, and efficacy in conditions from IBD to allergies and even cancer. (c) Employing advanced “omics” technologies: Genomics, transcriptomics, proteomics, and metabolomics can pinpoint host–MAMP interactions at the molecular level. This approach helps unravel how structural variations in MAMPs (e.g., differences in glycosylation or acylation) translate into distinct immune outcomes, and how individual host factors modulate responses.

Overall, harnessing lactobacilli MAMPs for targeted immunomodulation is a promising and rapidly growing field with enormous therapeutic potential. As the mechanistic underpinnings become clearer, more precise and potent postbiotic interventions are likely to emerge, bridging the gap between traditional probiotic usage and next-generation immunotherapies.

## Figures and Tables

**Table 1:** Comparative features of lactobacilli’s MAMPs versus other genera.

MAMP	Lactobacilli: Recurrent Feature	Comparison with Other Genera and Functional Implication	Refs.
Peptidoglycan	Muropeptide patterns and cell-wall modifications (e.g., O-acetylation via OatA, amidation) tune NOD1/NOD2 sensing and lysozyme susceptibility; in several lactobacilli strains, PGN/fragments tend to dampen IL-12 and NF-κB outputs, favoring regulatory/barrier-supportive profiles.	In pathogens, PGN modifications often promote lysozyme evasion and strong NOD/TLR priming, and the outcome is a pro-inflammatory bias.	[24,118,274,275,276]
Lipoteichoic acid	The degree of D-alanine substitution (operon dlt) acts as a “rheostat”: reduced D-alanylation attenuates TLR2 signaling and inflammation. Lactobacilli LTA can increase IL-10 and reduce MAPK/NF-κB in epithelial/monocytic models.	In many Gram-positive pathogens, highly D-alanylated LTA drives robust TLR2-dependent activation and pro-inflammatory cytokines.	[154,277]
Exopolysaccharides	Frequently acidic/branched, high-MW polymers that modulate TLR2/TLR4–MAPK/STAT pathways, lower pro-inflammatory cytokines, and strengthen tight junctions (barrier-protective profile).	Pathogen EPS/capsules favor adhesion/biofilm and immune evasion; outputs are often pro-inflammatory or broadly suppressive without barrier-repair features.	[25,278,279]
S-layer	S-layer of *L. acidophilus* (SlpA) is a DC-SIGN ligand and cooperates with TLR2 to reshape DC functions and T-cell output (regulatory bias). Recent work reinforces lectin-PRR (DC-SIGN) engagement and immunoregulation in Lactobacilli.	S-layers are not ubiquitous among commensals; in pathogens, S-layers are often linked to innate stimulation (via TLR/CLR) and/or evasion. A DC-SIGN-centric, tolerogenic signature is more typical of lactobacilli.	[221,280,281]
Pili	SpaCBA pili of *L. rhamnosus* GG mediate high-affinity mucus adhesion and can modulate epithelial/DC cytokines (e.g., IL-8); mutant/heterologous systems reveal a contribution of pili to non-pathogenic DC/IEC crosstalk.	In pathogens (e.g., pneumococcus, streptococcus), pili act as pro-inflammatory MAMPs (often TLR2-dependent), boosting IL-8/TNF-α and virulence.	[238,239,282]

## Data Availability

Data is contained within the article and Supplementary Materials.

## Supplementary Materials

The following supporting information can be downloaded at https://www.mdpi.com/article/10.3390/biom15111609/s1. Table S1: Summary of immunomodulatory effects induced by lactobacillus-derived MAMPs in in vitro, ex vivo, and in vivo models.

## Abbreviations

The following abbreviations are used in this manuscript:

MAMP	Microorganism-Associated Molecular Pattern
PRR	Pattern Recognition Receptor
TLR	Toll-Like Receptor
IL	Interleukin
TNF-α	Tumor Necrosis Factor α
T_reg_	Regulatory T-Cell
ROS	Reactive Oxygen Species
GRAS	Generally Recognized as Safe
LTA	Lipotheicoic Acid
PGN	Peptydoglycan
EPS	Exopolysaccharide
MAPK	Mitogen-Activated Protein Kinase
NLR	NOD-Like Receptor
T_h_	T Helper Cell
NO	Nitric Oxide
PGE_2_	Prostaglandin E_2_
iNOS	Inducible Nitric Oxide Syntase
IFN-γ	Interferon-γ
LPS	Lipopolysaccharide
NK	Natural Killer
DAI	Disease Activity Index
LGG	*L. rhamnosus* GG
DC	Dendritic Cell
CFS	Cell-Free Supernatant
ERK	Extracellular Signal-Regulated Kinase
JNK	c-jun N-Terminal Kinase-Dependent
CLR	C-Type Lectin Receptor
RLR	RIG-1-Like Receptor
ALR	AIM-2 Like Receptor
LRR	Leucine-Rich Repeat
TIR	Toll-Interleukin 1 Receptor
PAMP	Pathogen-Associated Molecular Pattern
DAMP	Damage-Associated Molecular Pattern
XAMP	Xeno-Associated Molecular Pattern
APC	Antigen-Presenting Cell
MD-2	Myeloid Differentiation Factor 2
LBP	LPS Binding Protein
MyD88	Myeloid Differentiation Primary Response 88
TRIF	TIR Domain Containing Adaptor-Inducing Interferon-β
TIRAP/MAL	TIR Domain Containing Adaptor Protein
DD	Death Domain
IRAK	IL-1 Receptor-Associated Kinase
TRAF	TNF Receptor-Associated Factor
TAK	TGF-β-Activated Kinase
IKK	IκB Kinase
IRF	IFN-Regulatory Factor
RIP	Receptor-Interacting Protein
TRAM	TRIF-Related Adaptor
BIR	Baculoviral Inhibitory Repeat-Like Domain
NAIP	NLR Family Apoptosis Inhibitory Protein
CARD	Caspase Activation and Recruitment Domain
PYR	Pyrin Domain
ASC	Apoptosis Associated Speck-Like Protein-Containing CARD
MDP	Muramyl Dipeptide
TnSS	Type n Secretion System
BMDM	Bone Marrow-Derived Macrophages
TA	Teichoic Acid
WT	Wild-Type
pLTA	LTA from *L. plantarum*
ETEC	Enterotoxigenic *E. coli*
rhLTA	LTA from *L. rhamnosus*
RSV	Respiratory Syncytial Virus
AM	Alveolar Macrophage
PSPG	Polysaccharide–Peptidoglycan Complex
PGH	Peptidoglycan Hydrolase
MoDC	Monocyte-Derived Dendritic Cell
cLTA	LTA from *L. casei*
PUFA	Poly-Unsaturated Fatty Acid
aLTA	LTA from *L. acidophilus*
sLTA	LTA from *L. sakei*
dLTA	LTA from *L. delbrueckii*
pEPS	EPS from *L. plantarum*
T-AOC	Total Antioxidant Capacity
SOD	Superoxide Dismutase
GHS-Px	Gluthatione Peroxidase
CAT	Catalase
rhEPS	EPS from *L. rhamnosus*
cEPS	EPS from *L. casei*
pcEPS	EPS from *L. paracasei*
dbEPS	EPS from *L. delbrueckii* spp. *bulgaricus*
aEPS	EPS from *L. acidophilus*
reEPS	EPS from *L. reuteri*
SLP	S-Layer Protein
ISG	Interferon I-Stimulated Gene
MIMP	Micro Integral Membrane Protein
gDNA	Genomic DNA
ODN	Oligodeoxynucleotide
MV	Membrane Vesicles
IBLP	Immunobiotic-Like Particle
EV	Extracellular Vesicles

## References

[1] Dempsey E., Corr S.C. (2022). *Lactobacillus* spp. for Gastrointestinal Health: Current and Future Perspectives. Front. Immunol..

[2] Zheng J., Wittouck S., Salvetti E., Franz C., Harris H.M.B., Mattarelli P., O’Toole P.W., Pot B., Vandamme P., Walter J. (2020). A taxonomic note on the genus *Lactobacillus*: Description of 23 novel genera, emended description of the genus *Lactobacillus* Beijerinck 1901, and union of *Lactobacillaceae* and *Leuconostocaceae*. Int. J. Syst. Evol. Microbiol..

[3] Fuochi V., Cardile V., Petronio Petronio G., Furneri P.M. (2018). Biological properties and production of bacteriocins-like-inhibitory substances by *Lactobacillus* sp. strains from human vagina. J. Appl. Microbiol..

[4] Fuochi V., Li Volti G., Furneri P.M. (2017). Commentary: Lactobacilli Dominance and Vaginal pH: Why Is the Human Vaginal Microbiome Unique?. Front. Microbiol..

[5] Laudani S., Torrisi S.A., Alboni S., Bastiaanssen T.F.S., Benatti C., Rivi V., Moloney R.D., Fuochi V., Furneri P.M., Drago F. (2023). Gut microbiota alterations promote traumatic stress susceptibility associated with p-cresol-induced dopaminergic dysfunctions. Brain Behav. Immun..

[6] Fuochi V. (2016). Amensalistic Activity of *Lactobacillus* spp. Isolated from Human Samples. Ph.D. Thesis.

[7] Fuochi V., Li Volti G., Furneri P.M. (2017). Probiotic Properties of *Lactobacillus fermentum* Strains Isolated from Human Oral Samples and Description of their Antibacterial Activity. Curr. Pharm. Biotechnol..

[8] Fuochi V. (2018). Antifungal Activity of Extracts Produced by *Lactobacillus fermentum* Strains and Analysis of Candida Albicans Yeast/Mold (Y/M) Switching. J. Clin. Gastroenterol..

[9] Rocha-Ramirez L.M., Hernandez-Chinas U., Moreno-Guerrero S.S., Ramirez-Pacheco A., Eslava C.A. (2021). Probiotic Properties and Immunomodulatory Activity of *Lactobacillus* Strains Isolated from Dairy Products. Microorganisms.

[10] Stivala A., Carota G., Fuochi V., Furneri P.M. (2021). *Lactobacillus rhamnosus* AD3 as a Promising Alternative for Probiotic Products. Biomolecules.

[11] Rastogi S., Singh A. (2022). Gut microbiome and human health: Exploring how the probiotic genus *Lactobacillus* modulate immune responses. Front. Pharmacol..

[12] Fuochi V., Spampinato M., Distefano A., Palmigiano A., Garozzo D., Zagni C., Rescifina A., Li Volti G., Furneri P.M. (2023). Soluble peptidoglycan fragments produced by *Limosilactobacillus fermentum* with antiproliferative activity are suitable for potential therapeutic development: A preliminary report. Front. Mol. Biosci..

[13] Swanson K.S., Gibson G.R., Hutkins R., Reimer R.A., Reid G., Verbeke K., Scott K.P., Holscher H.D., Azad M.B., Delzenne N.M. (2020). The International Scientific Association for Probiotics and Prebiotics (ISAPP) consensus statement on the definition and scope of synbiotics. Nat. Rev. Gastroenterol. Hepatol..

[14] Garbacz K. (2022). Anticancer activity of lactic acid bacteria. Semin. Cancer Biol..

[15] Samanta S. (2022). Potential Impacts of Prebiotics and Probiotics on Cancer Prevention. Anticancer Agents Med. Chem..

[16] Kojima M., Wakai K., Tamakoshi K., Tokudome S., Toyoshima H., Watanabe Y., Hayakawa N., Suzuki K., Hashimoto S., Ito Y. (2004). Diet and colorectal cancer mortality: Results from the Japan Collaborative Cohort Study. Nutr. Cancer.

[17] Pala V., Sieri S., Berrino F., Vineis P., Sacerdote C., Palli D., Masala G., Panico S., Mattiello A., Tumino R. (2011). Yogurt consumption and risk of colorectal cancer in the Italian European prospective investigation into cancer and nutrition cohort. Int. J. Cancer.

[18] Fuochi V., Coniglio M.A., Laghi L., Rescifina A., Caruso M., Stivala A., Furneri P.M. (2019). Metabolic Characterization of Supernatants Produced by *Lactobacillus* spp. With In vitro Anti-Legionella Activity. Front. Microbiol..

[19] Tarique M., Abdalla A., Masad R., Al-Sbiei A., Kizhakkayil J., Osaili T., Olaimat A., Liu S.-Q., Fernandez-Cabezudo M., al-Ramadi B. (2022). Potential probiotics and postbiotic characteristics including immunomodulatory effects of lactic acid bacteria isolated from traditional yogurt-like products. Lwt.

[20] Vinderola G., Sanders M.E., Salminen S. (2022). The Concept of Postbiotics. Foods.

[21] Delgado S., Sanchez B., Margolles A., Ruas-Madiedo P., Ruiz L. (2020). Molecules Produced by Probiotics and Intestinal Microorganisms with Immunomodulatory Activity. Nutrients.

[22] Lebeer S., Vanderleyden J., De Keersmaecker S.C. (2010). Host interactions of probiotic bacterial surface molecules: Comparison with commensals and pathogens. Nat. Rev. Microbiol..

[23] You X., Li Z., Ma K., Zhang C., Chen X., Wang G., Yang L., Dong M., Rui X., Zhang Q. (2020). Structural characterization and immunomodulatory activity of an exopolysaccharide produced by *Lactobacillus helveticus* LZ-R-5. Carbohydr. Polym..

[24] Gao J., Wang L., Jiang J., Xu Q., Zeng N., Lu B., Yuan P., Sun K., Zhou H., He X. (2023). A probiotic bi-functional peptidoglycan hydrolase sheds NOD2 ligands to regulate gut homeostasis in female mice. Nat. Commun..

[25] Teame T., Wang A., Xie M., Zhang Z., Yang Y., Ding Q., Gao C., Olsen R.E., Ran C., Zhou Z. (2020). Paraprobiotics and postbiotics of probiotic Lactobacilli, their positive effects on the host and action mechanisms: A review. Front. Nutr..

[26] Fong F.L.Y., Shah N.P., Kirjavainen P., El-Nezami H. (2016). Mechanism of action of probiotic bacteria on intestinal and systemic immunities and antigen-presenting cells. Int. Rev. Immunol..

[27] Metchnikoff I.I. (2004). The Prolongation of Life: Optimistic Studies.

[28] Ouwehand A.C., Salminen S., Isolauri E. (2002). Probiotics: An overview of beneficial effects. Antonie Van Leeuwenhoek.

[29] Park C., Ji S.Y., Hwangbo H., Shin S.Y., Kim M.Y., Lee K., Kim D.H., Cho B.R., Lee H., Choi Y.H. (2023). Enhancement of Immune Functions by *Limosilactobacillus reuteri* KBL346: In vitro and In Vivo Studies. Int. J. Mol. Sci..

[30] Lee J., Jung I., Choi J.W., Lee C.W., Cho S., Choi T.G., Sohn M., Park Y.I. (2019). Micronized and Heat-Treated *Lactobacillus plantarum* LM1004 Stimulates Host Immune Responses Via the TLR-2/MAPK/NF-kappaB Signalling Pathway In vitro and In Vivo. J. Microbiol. Biotechnol..

[31] Chondrou P., Karapetsas A., Kiousi D.E., Vasileiadis S., Ypsilantis P., Botaitis S., Alexopoulos A., Plessas S., Bezirtzoglou E., Galanis A. (2020). Assessment of the Immunomodulatory Properties of the Probiotic Strain *Lactobacillus paracasei* K5 In vitro and In Vivo. Microorganisms.

[32] Perez-Cano F.J., Dong H., Yaqoob P. (2010). In vitro immunomodulatory activity of *Lactobacillus fermentum* CECT5716 and *Lactobacillus salivarius* CECT5713: Two probiotic strains isolated from human breast milk. Immunobiology.

[33] Kishimoto M., Nomoto R., Mizuno M., Osawa R. (2017). An In vitro investigation of immunomodulatory properties of *Lactobacillus plantarum* and *L. delbrueckii* cells and their extracellular polysaccharides. Biosci. Microbiota Food Health.

[34] Salva S., Villena J., Alvarez S. (2010). Immunomodulatory activity of *Lactobacillus rhamnosus* strains isolated from goat milk: Impact on intestinal and respiratory infections. Int. J. Food Microbiol..

[35] Min F., Hu J., Huang T., Huang Y., Nie S., Xiong T., Xie M. (2023). Effects of *Lactobacillus casei* NCU011054 on immune response and gut microbiota of cyclophosphamide induced immunosuppression mice. Food Chem. Toxicol..

[36] Michels M., Jesus G.F.A., Abatti M.R., Corneo E., Cucker L., de Medeiros Borges H., da Silva Matos N., Rocha L.B., Dias R., Simon C.S. (2022). Effects of different probiotic strains *B. lactis*, *L. rhamnosus* and *L. reuteri* on brain-intestinal axis immunomodulation in an en-dotoxin-induced inflammation. Mol. Neurobiol..

[37] Qiao J., Sun Z., Liang D., Li H. (2020). Lactobacillus salivarius alleviates inflammation via NF-kappaB signaling in ETEC K88-induced IPEC-J2 cells. J. Anim. Sci. Biotechnol..

[38] Wong W.Y., Chan B.D., Sham T.T., Lee M.M., Chan C.O., Chau C.T., Mok D.K., Kwan Y.W., Tai W.C. (2022). *Lactobacillus casei* Strain Shirota Ameliorates Dextran Sulfate Sodium-Induced Colitis in Mice by Increasing Taurine-Conjugated Bile Acids and Inhibiting NF-kappaB Signaling via Stabilization of IkappaBalpha. Front. Nutr..

[39] Sun Z., Huang S., Yan X., Zhang X., Hao Y., Jiang L., Dai Z. (2024). Living, Heat-Killed *Limosilactobacillus mucosae* and Its Cell-Free Supernatant Differentially Regulate Colonic Serotonin Receptors and Immune Response in Experimental Colitis. Nutrients.

[40] Mata Forsberg M., Bjorkander S., Pang Y., Lundqvist L., Ndi M., Ott M., Escriba I.B., Jaeger M.C., Roos S., Sverremark-Ekstrom E. (2019). Extracellular Membrane Vesicles from Lactobacilli Dampen IFN-gamma Responses in a Monocyte-Dependent Manner. Sci. Rep..

[41] Jung J.Y., Shin J.S., Lee S.G., Rhee Y.K., Cho C.W., Hong H.D., Lee K.T. (2015). *Lactobacillus sakei* K040706 evokes immunostimulatory effects on macrophages through TLR 2-mediated activation. Int. Immunopharmacol..

[42] Si W., Liang H., Bugno J., Xu Q., Ding X., Yang K., Fu Y., Weichselbaum R.R., Zhao X., Wang L. (2022). *Lactobacillus rhamnosus* GG induces cGAS/STING- dependent type I interferon and improves response to immune checkpoint blockade. Gut.

[43] Wang X., Tang J., Zhang S., Zhang N. (2022). Effects of *Lactiplantibacillus plantarum* 19-2 on immunomodulatory function and gut microbiota in mice. Front. Microbiol..

[44] Johansson M.A., Bjorkander S., Mata Forsberg M., Qazi K.R., Salvany Celades M., Bittmann J., Eberl M., Sverremark-Ekstrom E. (2016). Probiotic Lactobacilli Modulate *Staphylococcus aureus*-Induced Activation of Conventional and Unconventional T cells and NK Cells. Front. Immunol..

[45] Yamasaki-Yashiki S., Kawashima F., Saika A., Hosomi R., Kunisawa J., Katakura Y. (2024). RNA-Based Anti-Inflammatory Effects of Membrane Vesicles Derived from *Lactiplantibacillus plantarum*. Foods.

[46] Holowacz S., Blondeau C., Guinobert I., Guilbot A., Hidalgo S., Bisson J.F. (2018). *Lactobacillus salivarius* LA307 and *Lactobacillus rhamnosus* LA305 attenuate skin inflammation in mice. Benef. Microbes.

[47] Park S.W., Choi Y.H., Gho J.Y., Kang G.A., Kang S.S. (2024). Synergistic Inhibitory Effect of *Lactobacillus* Cell Lysates and Butyrate on Poly I:C-Induced IL-8 Production in Human Intestinal Epithelial Cells. Probiotics Antimicrob. Proteins.

[48] Kim M.Y., Hyun I.K., An S., Kim D., Kim K.H., Kang S.S. (2022). In vitro anti-inflammatory and antibiofilm activities of bacterial lysates from lactobacilli against oral pathogenic bacteria. Food Funct..

[49] McClure R., Massari P. (2014). TLR-Dependent Human Mucosal Epithelial Cell Responses to Microbial Pathogens. Front. Immunol..

[50] Duan T., Du Y., Xing C., Wang H.Y., Wang R.F. (2022). Toll-Like Receptor Signaling and Its Role in Cell-Mediated Immunity. Front. Immunol..

[51] Wallace T.D., Bradley S., Buckley N.D., Green-Johnson J.M. (2003). Interactions of lactic acid bacteria with human intestinal epithelial cells: Effects on cytokine production. J. Food Prot..

[52] Borruel N., Casellas F., Antolin M., Llopis M., Carol M., Espiin E., Naval J., Guarner F., Malagelada J.R. (2003). Effects of nonpathogenic bacteria on cytokine secretion by human intestinal mucosa. Am. J. Gastroenterol..

[53] Paturi G., Phillips M., Jones M., Kailasapathy K. (2007). Immune enhancing effects of *Lactobacillus acidophilus* LAFTI L10 and *Lactobacillus paracasei* LAFTI L26 in mice. Int. J. Food Microbiol..

[54] Ciesielska A., Matyjek M., Kwiatkowska K. (2021). TLR4 and CD14 trafficking and its influence on LPS-induced pro-inflammatory signaling. Cell. Mol. Life Sci..

[55] Zanoni I., Ostuni R., Marek L.R., Barresi S., Barbalat R., Barton G.M., Granucci F., Kagan J.C. (2011). CD14 controls the LPS-induced endocytosis of Toll-like receptor 4. Cell.

[56] Stack J., Doyle S.L., Connolly D.J., Reinert L.S., O’Keeffe K.M., McLoughlin R.M., Paludan S.R., Bowie A.G. (2014). TRAM is required for TLR2 endosomal signaling to type I IFN induction. J. Immunol..

[57] Fitzgerald K.A., Kagan J.C. (2020). Toll-like Receptors and the Control of Immunity. Cell.

[58] Collins P.E., Carmody R.J. (2015). The Regulation of Endotoxin Tolerance and its Impact on Macrophage Activation. Crit. Rev. Immunol..

[59] Netea M.G., Domínguez-Andrés J., Barreiro L.B., Chavakis T., Divangahi M., Fuchs E., Joosten L.A., van der Meer J.W., Mhlanga M.M., Mulder W.J. (2020). Defining trained immunity and its role in health and disease. Nat. Rev. Immunol..

[60] Gantner B.N., Simmons R.M., Canavera S.J., Akira S., Underhill D.M. (2003). Collaborative induction of inflammatory responses by dectin-1 and Toll-like receptor 2. J. Exp. Med..

[61] Okai N., Masuta Y., Otsuka Y., Hara A., Masaki S., Kamata K., Minaga K., Honjo H., Kudo M., Watanabe T. (2024). Crosstalk between NOD2 and TLR2 suppresses the development of TLR2-mediated experimental colitis. J. Clin. Biochem. Nutr..

[62] Kelley N., Jeltema D., Duan Y., He Y. (2019). The NLRP3 inflammasome: An overview of mechanisms of activation and regulation. Int. J. Mol. Sci..

[63] Kobayashi K., Hernandez L.D., Galán J.E., Janeway C.A., Medzhitov R., Flavell R.A. (2002). IRAK-M is a negative regulator of Toll-like receptor signaling. Cell.

[64] Ma A., Malynn B.A. (2012). A20: Linking a complex regulator of ubiquitylation to immunity and human disease. Nat. Rev. Immunol..

[65] Wald D., Qin J., Zhao Z., Qian Y., Naramura M., Tian L., Towne J., Sims J.E., Stark G.R., Li X. (2003). SIGIRR, a negative regulator of Toll-like receptor–interleukin 1 receptor signaling. Nat. Immunol..

[66] Zhang G., Ghosh S. (2002). Negative regulation of toll-like receptor-mediated signaling by Tollip. J. Biol. Chem..

[67] O’Neill L.A., Kishton R.J., Rathmell J. (2016). A guide to immunometabolism for immunologists. Nat. Rev. Immunol..

[68] Hoque R., Farooq A., Ghani A., Gorelick F., Mehal W.Z. (2014). Lactate reduces liver and pancreatic injury in toll-like receptor–and inflammasome-mediated inflammation via GPR81-mediated suppression of innate immunity. Gastroenterology.

[69] Yang K., Xu J., Fan M., Tu F., Wang X., Ha T., Williams D.L., Li C. (2020). Lactate Suppresses Macrophage Pro-Inflammatory Response to LPS Stimulation by Inhibition of YAP and NF-κB Activation via GPR81-Mediated Signaling. Front. Immunol..

[70] Morgan M.J., Liu Z.-g. (2011). Crosstalk of reactive oxygen species and NF-κB signaling. Cell Res..

[71] Coombes J.L., Siddiqui K.R., Arancibia-Cárcamo C.V., Hall J., Sun C.-M., Belkaid Y., Powrie F. (2007). A functionally specialized population of mucosal CD103+ DCs induces Foxp3+ regulatory T cells via a TGF-β–and retinoic acid–dependent mechanism. J. Exp. Med..

[72] Mucida D., Park Y., Kim G., Turovskaya O., Scott I., Kronenberg M., Cheroutre H. (2007). Reciprocal TH17 and regulatory T cell differentiation mediated by retinoic acid. Science.

[73] Cario E., Gerken G., Podolsky D.K. (2004). Toll-like receptor 2 enhances ZO-1-associated intestinal epithelial barrier integrity via protein kinase C. Gastroenterology.

[74] Eisenbarth S. (2019). Dendritic cell subsets in T cell programming: Location dictates function. Nat. Rev. Immunol..

[75] Ostuni R., Piccolo V., Barozzi I., Polletti S., Termanini A., Bonifacio S., Curina A., Prosperini E., Ghisletti S., Natoli G. (2013). Latent enhancers activated by stimulation in differentiated cells. Cell.

[76] Jia D.J., Wang Q.W., Hu Y.Y., He J.M., Ge Q.W., Qi Y.D., Chen L.Y., Zhang Y., Fan L.N., Lin Y.F. (2022). *Lactobacillus johnsonii* alleviates colitis by TLR1/2-STAT3 mediated CD206(+) macrophages(IL-10) activation. Gut Microbes.

[77] Chen Y., Guan W., Zhang N., Wang Y., Tian Y., Sun H., Li X., Wang Y., Liu J. (2022). *Lactobacillus plantarum* Lp2 improved LPS-induced liver injury through the TLR-4/MAPK/NFkappaB and Nrf2-HO-1/CYP2E1 pathways in mice. Food Nutr. Res..

[78] Onishi K., Mochizuki J., Sato A., Goto A., Sashihara T. (2020). Total RNA and genomic DNA of *Lactobacillus gasseri* OLL2809 induce interleukin-12 production in the mouse macrophage cell line J774.1 via toll-like receptors 7 and 9. BMC Microbiol..

[79] Kingma S.D., Li N., Sun F., Valladares R.B., Neu J., Lorca G.L. (2011). *Lactobacillus johnsonii* N6.2 stimulates the innate immune response through Toll-like receptor 9 in Caco-2 cells and increases intestinal crypt Paneth cell number in biobreeding diabetes-prone rats. J. Nutr..

[80] Zhang Y., Li Y., Wang X., Huang J., Feng X., Shi C., Yang W., Jiang Y., Cao X., Wang J. (2023). *Lactobacillus plantarum* NC8 and its metabolite acetate alleviate type 1 diabetes via inhibiting NLRP3. Microb. Pathog..

[81] Li P., Chen G., Zhang J., Pei C., Chen Y., Gong J., Deng S., Cai K., Li H., Wang D. (2022). Live *Lactobacillus acidophilus* alleviates ulcerative colitis via the SCFAs/mitophagy/NLRP3 inflammasome axis. Food Funct..

[82] Suzuki H., Yamazaki T., Ohshio K., Sugamata M., Yoshikawa M., Kanauchi O., Morita Y. (2020). A Specific Strain of Lactic Acid Bacteria, *Lactobacillus paracasei*, Inhibits Inflammasome Activation In vitro and Prevents Inflammation-Related Disorders. J. Immunol..

[83] Wang S., Li H., Du C., Liu Q., Yang D., Chen L., Zhu Q., Wang Z. (2018). Effects of dietary supplementation with *Lactobacillus acidophilus* on the performance, intestinal physical barrier function, and the expression of NOD-like receptors in weaned piglets. PeerJ.

[84] Udayan S., Butto L.F., Rossini V., Velmurugan J., Martinez-Lopez M., Sancho D., Melgar S., O’Toole P.W., Nally K. (2021). Macrophage cytokine responses to commensal Gram-positive *Lactobacillus salivarius* strains are TLR2-independent and Myd88-dependent. Sci. Rep..

[85] Sameer A.S., Nissar S. (2021). Toll-Like Receptors (TLRs): Structure, Functions, Signaling, and Role of Their Polymorphisms in Colorectal Cancer Susceptibility. Biomed. Res. Int..

[86] Behzadi P., Garcia-Perdomo H.A., Karpinski T.M. (2021). Toll-Like Receptors: General Molecular and Structural Biology. J. Immunol. Res..

[87] Wells J.M. (2011). Immunomodulatory mechanisms of lactobacilli. Microb. Cell Fact..

[88] Medzhitov R. (2001). Toll-like receptors and innate immunity. Nat. Rev. Immunol..

[89] Brubaker S.W., Bonham K.S., Zanoni I., Kagan J.C. (2015). Innate immune pattern recognition: A cell biological perspective. Annu. Rev. Immunol..

[90] Kumar H., Kawai T., Akira S. (2011). Pathogen recognition by the innate immune system. Int. Rev. Immunol..

[91] Caplan I.F., Maguire-Zeiss K.A. (2018). Toll-Like Receptor 2 Signaling and Current Approaches for Therapeutic Modulation in Synucleinopathies. Front. Pharmacol..

[92] De Nardo D. (2015). Toll-like receptors: Activation, signalling and transcriptional modulation. Cytokine.

[93] Jeon D., Hill E., McNeel D.G. (2024). Toll-like receptor agonists as cancer vaccine adjuvants. Hum. Vaccin. Immunother..

[94] Kawai T., Akira S. (2011). Toll-like receptors and their crosstalk with other innate receptors in infection and immunity. Immunity.

[95] Farhat K., Riekenberg S., Heine H., Debarry J., Lang R., Mages J., Buwitt-Beckmann U., Roschmann K., Jung G., Wiesmuller K.H. (2008). Heterodimerization of TLR2 with TLR1 or TLR6 expands the ligand spectrum but does not lead to differential signaling. J. Leukoc. Biol..

[96] Hopkins P.A., Sriskandan S. (2005). Mammalian Toll-like receptors: To immunity and beyond. Clin. Exp. Immunol..

[97] Chen Y., Lin J., Zhao Y., Ma X., Yi H. (2021). Toll-like receptor 3 (TLR3) regulation mechanisms and roles in antiviral innate immune responses. J. Zhejiang Univ. Sci. B.

[98] Gewirtz A.T., Navas T.A., Lyons S., Godowski P.J., Madara J.L. (2001). Cutting edge: Bacterial flagellin activates basolaterally expressed TLR5 to induce epithelial proinflammatory gene expression. J. Immunol..

[99] Patinote C., Karroum N.B., Moarbess G., Cirnat N., Kassab I., Bonnet P.A., Deleuze-Masquefa C. (2020). Agonist and antagonist ligands of toll-like receptors 7 and 8: Ingenious tools for therapeutic purposes. Eur. J. Med. Chem..

[100] Bauer S., Wagner H. (2002). Bacterial CpG-DNA licenses TLR9. Curr. Top. Microbiol. Immunol..

[101] Kawagoe T., Sato S., Matsushita K., Kato H., Matsui K., Kumagai Y., Saitoh T., Kawai T., Takeuchi O., Akira S. (2008). Sequential control of Toll-like receptor-dependent responses by IRAK1 and IRAK2. Nat. Immunol..

[102] Wicherska-Pawlowska K., Wrobel T., Rybka J. (2021). Toll-Like Receptors (TLRs), NOD-Like Receptors (NLRs), and RIG-I-Like Receptors (RLRs) in Innate Immunity. TLRs, NLRs, and RLRs Ligands as Immunotherapeutic Agents for Hematopoietic Diseases. Int. J. Mol. Sci..

[103] Meylan E., Burns K., Hofmann K., Blancheteau V., Martinon F., Kelliher M., Tschopp J. (2004). RIP1 is an essential mediator of Toll-like receptor 3-induced NF-kappa B activation. Nat. Immunol..

[104] Uematsu S., Sato S., Yamamoto M., Hirotani T., Kato H., Takeshita F., Matsuda M., Coban C., Ishii K.J., Kawai T. (2005). Interleukin-1 receptor-associated kinase-1 plays an essential role for Toll-like receptor (TLR)7- and TLR9-mediated interferon-alpha induction. J. Exp. Med..

[105] Almeida-da-Silva C.L.C., Savio L.E.B., Coutinho-Silva R., Ojcius D.M. (2023). The role of NOD-like receptors in innate immunity. Front. Immunol..

[106] Rosenstiel P., Till A., Schreiber S. (2007). NOD-like receptors and human diseases. Microbes Infect..

[107] Chen G., Shaw M.H., Kim Y.G., Nunez G. (2009). NOD-like receptors: Role in innate immunity and inflammatory disease. Annu. Rev. Pathol..

[108] Schroder K., Tschopp J. (2010). The inflammasomes. Cell.

[109] Hara H., Seregin S.S., Yang D., Fukase K., Chamaillard M., Alnemri E.S., Inohara N., Chen G.Y., Nunez G. (2018). The NLRP6 Inflammasome Recognizes Lipoteichoic Acid and Regulates Gram-Positive Pathogen Infection. Cell.

[110] Kim Y.K., Shin J.S., Nahm M.H. (2016). NOD-Like Receptors in Infection, Immunity, and Diseases. Yonsei Med. J..

[111] Damm A., Lautz K., Kufer T.A. (2013). Roles of NLRP10 in innate and adaptive immunity. Microbes Infect..

[112] Chamaillard M., Hashimoto M., Horie Y., Masumoto J., Qiu S., Saab L., Ogura Y., Kawasaki A., Fukase K., Kusumoto S. (2003). An essential role for NOD1 in host recognition of bacterial peptidoglycan containing diaminopimelic acid. Nat. Immunol..

[113] Platnich J.M., Muruve D.A. (2019). NOD-like receptors and inflammasomes: A review of their canonical and non-canonical signaling pathways. Arch. Biochem. Biophys..

[114] Rohde M. (2019). The Gram-Positive Bacterial Cell Wall. Microbiol. Spectr..

[115] Neuhaus F.C., Baddiley J. (2003). A continuum of anionic charge: Structures and functions of D-alanyl-teichoic acids in gram-positive bacteria. Microbiol. Mol. Biol. Rev..

[116] Schleifer K.H., Kandler O. (1972). Peptidoglycan types of bacterial cell walls and their taxonomic implications. Bacteriol. Rev..

[117] Vollmer W., Blanot D., de Pedro M.A. (2008). Peptidoglycan structure and architecture. FEMS Microbiol. Rev..

[118] Bersch K.L., DeMeester K.E., Zagani R., Chen S., Wodzanowski K.A., Liu S., Mashayekh S., Reinecker H.C., Grimes C.L. (2021). Bacterial Peptidoglycan Fragments Differentially Regulate Innate Immune Signaling. ACS Cent. Sci..

[119] Wu Z., Pan D., Guo Y., Sun Y., Zeng X. (2015). Peptidoglycan diversity and anti-inflammatory capacity in *Lactobacillus* strains. Carbohydr. Polym..

[120] Irazoki O., Hernandez S.B., Cava F. (2019). Peptidoglycan Muropeptides: Release, Perception, and Functions as Signaling Molecules. Front. Microbiol..

[121] Zeuthen L.H., Fink L.N., Frokiaer H. (2008). Toll-like receptor 2 and nucleotide-binding oligomerization domain-2 play divergent roles in the recognition of gut-derived lactobacilli and bifidobacteria in dendritic cells. Immunology.

[122] Sun Q., Liu X., Li X. (2022). Peptidoglycan-based immunomodulation. Appl. Microbiol. Biotechnol..

[123] Sun J., Shi Y.H., Le G.W., Ma X.Y. (2005). Distinct immune response induced by peptidoglycan derived from *Lactobacillus* sp.. World J. Gastroenterol..

[124] Salva S., Tiscornia I., Gutierrez F., Alvarez S., Bollati-Fogolin M. (2021). *Lactobacillus rhamnosus* postbiotic-induced immunomodulation as safer alternative to the use of live bacteria. Cytokine.

[125] Huang J., Li J., Li Q., Li L., Zhu N., Xiong X., Li G. (2020). Peptidoglycan derived from *Lactobacillus rhamnosus* MLGA up-regulates the expression of chicken beta-defensin 9 without triggering an inflammatory response. Innate Immun..

[126] Kolling Y., Salva S., Villena J., Alvarez S. (2018). Are the immunomodulatory properties of *Lactobacillus rhamnosus* CRL1505 peptidoglycan common for all Lactobacilli during respiratory infection in malnourished mice?. PLoS ONE.

[127] Kolling Y., Salva S., Villena J., Marranzino G., Alvarez S. (2015). Non-viable immunobiotic *Lactobacillus rhamnosus* CRL1505 and its peptidoglycan improve systemic and respiratory innate immune response during recovery of immunocompromised-malnourished mice. Int. Immunopharmacol..

[128] Clua P., Tomokiyo M., Raya Tonetti F., Islam M.A., Garcia Castillo V., Marcial G., Salva S., Alvarez S., Takahashi H., Kurata S. (2020). The Role of Alveolar Macrophages in the Improved Protection against Respiratory Syncytial Virus and Pneumococcal Superinfection Induced by the Peptidoglycan of *Lactobacillus rhamnosus* CRL1505. Cells.

[129] Zelaya H., Arellano-Arriagada L., Fukuyama K., Matsumoto K., Marranzino G., Namai F., Salva S., Alvarez S., Aguero G., Kitazawa H. (2023). *Lacticaseibacillus rhamnosus* CRL1505 Peptidoglycan Modulates the Inflammation-Coagulation Response Triggered by Poly(I:C) in the Respiratory Tract. Int. J. Mol. Sci..

[130] Raya Tonetti F., Clua P., Fukuyama K., Marcial G., Sacur J., Marranzino G., Tomokiyo M., Vizoso-Pinto G., Garcia-Cancino A., Kurata S. (2022). The Ability of Postimmunobiotics from *L. rhamnosus* CRL1505 to Protect against Respiratory Syncytial Virus and Pneumococcal Super-Infection Is a Strain-Dependent Characteristic. Microorganisms.

[131] Belkacem N., Serafini N., Wheeler R., Derrien M., Boucinha L., Couesnon A., Cerf-Bensussan N., Gomperts Boneca I., Di Santo J.P., Taha M.K. (2017). *Lactobacillus paracasei* feeding improves immune control of influenza infection in mice. PLoS ONE.

[132] Li A.L., Sun Y.Q., Du P., Meng X.C., Guo L., Li S., Zhang C. (2017). The Effect of *Lactobacillus actobacillus* Peptidoglycan on Bovine beta-Lactoglobulin-Sensitized Mice via TLR2/NF-kappaB Pathway. Iran. J. Allergy Asthma Immunol..

[133] Wu Z., Pan D.D., Guo Y., Zeng X. (2013). Structure and anti-inflammatory capacity of peptidoglycan from *Lactobacillus acidophilus* in RAW-264.7 cells. Carbohydr. Polym..

[134] Choi S.H., Lee S.H., Kim M.G., Lee H.J., Kim G.B. (2019). *Lactobacillus plantarum* CAU1055 ameliorates inflammation in lipopolysaccharide-induced RAW264.7 cells and a dextran sulfate sodium-induced colitis animal model. J. Dairy Sci..

[135] Song X., Li F., Zhang M., Xia Y., Ai L., Wang G. (2021). Effect of D-Ala-Ended Peptidoglycan Precursors on the Immune Regulation of *Lactobacillus plantarum* Strains. Front. Immunol..

[136] Canto E., Moga E., Ricart E., Garcia-Bosch O., Garcia-Planella E., Juarez C., Vidal S. (2009). MDP-Induced selective tolerance to TLR4 ligands: Impairment in NOD2 mutant Crohn’s disease patients. Inflamm. Bowel Dis..

[137] Kim D., Choi H., Oh H., Lee J., Hwang Y., Kang S.S. (2023). Mutanolysin-Digested Peptidoglycan of *Lactobacillus reuteri* Promotes the Inhibition of Porphyromonas gingivalis Lipopolysaccharide-Induced Inflammatory Responses through the Regulation of Signaling Cascades via TLR4 Suppression. Int. J. Mol. Sci..

[138] Matsumoto S., Hara T., Hori T., Mitsuyama K., Nagaoka M., Tomiyasu N., Suzuki A., Sata M. (2005). Probiotic *Lactobacillus*-induced improvement in murine chronic inflammatory bowel disease is associated with the down-regulation of pro-inflammatory cytokines in lamina propria mononuclear cells. Clin. Exp. Immunol..

[139] Matsumoto S., Hara T., Nagaoka M., Mike A., Mitsuyama K., Sako T., Yamamoto M., Kado S., Takada T. (2009). A component of polysaccharide peptidoglycan complex on *Lactobacillus* induced an improvement of murine model of inflammatory bowel disease and colitis-associated cancer. Immunology.

[140] Toth M., Muzsai S., Regulski K., Szendi-Szatmari T., Czimmerer Z., Rajnavolgyi E., Chapot-Chartier M.P., Bacsi A. (2022). The Phagocytosis of *Lacticaseibacillus casei* and Its Immunomodulatory Properties on Human Monocyte-Derived Dendritic Cells Depend on the Expression of Lc-p75, a Bacterial Peptidoglycan Hydrolase. Int. J. Mol. Sci..

[141] Macho Fernandez E., Valenti V., Rockel C., Hermann C., Pot B., Boneca I.G., Grangette C. (2011). Anti-inflammatory capacity of selected lactobacilli in experimental colitis is driven by NOD2-mediated recognition of a specific peptidoglycan-derived muropeptide. Gut.

[142] Hatano S., Hirose Y., Yamamoto Y., Murosaki S., Yoshikai Y. (2015). Scavenger receptor for lipoteichoic acid is involved in the potent ability of *Lactobacillus plantarum* strain L-137 to stimulate production of interleukin-12p40. Int. Immunopharmacol..

[143] Matsuguchi T., Takagi A., Matsuzaki T., Nagaoka M., Ishikawa K., Yokokura T., Yoshikai Y. (2003). Lipoteichoic acids from *Lactobacillus* strains elicit strong tumor necrosis factor alpha-inducing activities in macrophages through Toll-like receptor 2. Clin. Diagn. Lab. Immunol..

[144] Claes I.J., Segers M.E., Verhoeven T.L., Dusselier M., Sels B.F., De Keersmaecker S.C., Vanderleyden J., Lebeer S. (2012). Lipoteichoic acid is an important microbe-associated molecular pattern of *Lactobacillus rhamnosus* GG. Microb. Cell Fact..

[145] Kaji R., Kiyoshima-Shibata J., Nagaoka M., Nanno M., Shida K. (2010). Bacterial teichoic acids reverse predominant IL-12 production induced by certain *lactobacillus* strains into predominant IL-10 production via TLR2-dependent ERK activation in macrophages. J. Immunol..

[146] Wang S., Ahmadi S., Nagpal R., Jain S., Mishra S.P., Kavanagh K., Zhu X., Wang Z., McClain D.A., Kritchevsky S.B. (2020). Lipoteichoic acid from the cell wall of a heat killed *Lactobacillus paracasei* D3-5 ameliorates aging-related leaky gut, inflammation and improves physical and cognitive functions: From C. elegans to mice. Geroscience.

[147] Saber R., Zadeh M., Pakanati K.C., Bere P., Klaenhammer T., Mohamadzadeh M. (2011). Lipoteichoic acid-deficient *Lactobacillus acidophilus* regulates downstream signals. Immunotherapy.

[148] Kim Y., Park J.Y., Kim H., Chung D.K. (2020). Differential role of lipoteichoic acids isolated from *Staphylococcus aureus* and *Lactobacillus plantarum* on the aggravation and alleviation of atopic dermatitis. Microb. Pathog..

[149] Jeon B., Kim H.R., Kim H., Chung D.K. (2016). In vitro and in vivo downregulation of C3 by lipoteichoic acid isolated from *Lactobacillus plantarum* K8 suppressed cytokine-mediated complement system activation. FEMS Microbiol. Lett..

[150] Kim J.Y., Kim H., Jung B.J., Kim N.R., Park J.E., Chung D.K. (2013). Lipoteichoic acid isolated from *Lactobacillus plantarum* suppresses LPS-mediated atherosclerotic plaque inflammation. Mol. Cells.

[151] Kim H., Jung B.J., Jung J.H., Kim J.Y., Chung S.K., Chung D.K. (2012). *Lactobacillus plantarum* lipoteichoic acid alleviates TNF-alpha-induced inflammation in the HT-29 intestinal epithelial cell line. Mol. Cells.

[152] Mizuno H., Arce L., Tomotsune K., Albarracin L., Funabashi R., Vera D., Islam M.A., Vizoso-Pinto M.G., Takahashi H., Sasaki Y. (2020). Lipoteichoic Acid Is Involved in the Ability of the Immunobiotic Strain *Lactobacillus plantarum* CRL1506 to Modulate the Intestinal Antiviral Innate Immunity Triggered by TLR3 Activation. Front. Immunol..

[153] Lu Q., Guo Y., Yang G., Cui L., Wu Z., Zeng X., Pan D., Cai Z. (2022). Structure and Anti-Inflammation Potential of Lipoteichoic Acids Isolated from *Lactobacillus* Strains. Foods.

[154] Kim K.W., Kang S.S., Woo S.J., Park O.J., Ahn K.B., Song K.D., Lee H.K., Yun C.H., Han S.H. (2017). Lipoteichoic Acid of Probiotic *Lactobacillus plantarum* Attenuates Poly I:C-Induced IL-8 Production in Porcine Intestinal Epithelial Cells. Front. Microbiol..

[155] Ryu Y.H., Baik J.E., Yang J.S., Kang S.S., Im J., Yun C.H., Kim D.W., Lee K., Chung D.K., Ju H.R. (2009). Differential immunostimulatory effects of Gram-positive bacteria due to their lipoteichoic acids. Int. Immunopharmacol..

[156] Lee B., Yin X., Griffey S.M., Marco M.L. (2015). Attenuation of Colitis by *Lactobacillus casei* BL23 Is Dependent on the Dairy Delivery Matrix. Appl. Environ. Microbiol..

[157] Smelt M.J., de Haan B.J., Bron P.A., van Swam I., Meijerink M., Wells J.M., Kleerebezem M., Faas M.M., de Vos P. (2013). The impact of *Lactobacillus plantarum* WCFS1 teichoic acid D-alanylation on the generation of effector and regulatory T-cells in healthy mice. PLoS ONE.

[158] Claes I., Lebeer S., Shen C., Verhoeven T., Dilissen E., De Hertogh G., Bullens D., Ceuppens J., Van Assche G., Vermeire S. (2010). Impact of lipoteichoic acid modification on the performance of the probiotic *Lactobacillus rhamnosus* GG in experimental colitis. Clin. Exp. Immunol..

[159] Cui L., Zeng H., Hou M., Li Z., Mu C., Zhu W., Hang S. (2023). *Lactiplantibacillus plantarum* L47 and inulin alleviate enterotoxigenic *Escherichia coli* induced ileal inflammation in piglets by upregulating the levels of alpha-linolenic acid and 12,13-epoxyoctadecenoic acid. Anim. Nutr..

[160] Hong Y.F., Kim H., Kim H.R., Gim M.G., Chung D.K. (2014). Different immune regulatory potential of *Lactobacillus plantarum* and Lactobacillus sakei isolated from Kimchi. J. Microbiol. Biotechnol..

[161] Pradhan D., Gulati G., Avadhani R., H M.R., Soumya K., Kumari A., Gupta A., Dwivedi D., Kaushik J.K., Grover S. (2023). Postbiotic Lipoteichoic acid of probiotic *Lactobacillus* origin ameliorates inflammation in HT-29 cells and colitis mice. Int. J. Biol. Macromol..

[162] Jeong J.H., Jang S., Jung B.J., Jang K.S., Kim B.G., Chung D.K., Kim H. (2015). Differential immune-stimulatory effects of LTAs from different lactic acid bacteria via MAPK signaling pathway in RAW 264.7 cells. Immunobiology.

[163] Noh S.Y., Kang S.S., Yun C.H., Han S.H. (2015). Lipoteichoic acid from *Lactobacillus plantarum* inhibits Pam2CSK4-induced IL-8 production in human intestinal epithelial cells. Mol. Immunol..

[164] Ahn J.E., Kim H., Chung D.K. (2019). Lipoteichoic Acid Isolated from *Lactobacillus plantarum* Maintains Inflammatory Homeostasis through Regulation of Th1- and Th2-Induced Cytokines. J. Microbiol. Biotechnol..

[165] Friedrich A.D., Campo V.E., Cela E.M., Morelli A.E., Shufesky W.J., Tckacheva O.A., Leoni J., Paz M.L., Larregina A.T., Gonzalez Maglio D.H. (2019). Oral administration of lipoteichoic acid from *Lactobacillus rhamnosus* GG overcomes UVB-induced immunosuppression and impairs skin tumor growth in mice. Eur. J. Immunol..

[166] Friedrich A.D., Leoni J., Paz M.L., Maglio D.H.G. (2022). Lipoteichoic Acid from *Lacticaseibacillus rhamnosus* GG Modulates Dendritic Cells and T Cells in the Gut. Nutrients.

[167] Mohamadzadeh M., Pfeiler E.A., Brown J.B., Zadeh M., Gramarossa M., Managlia E., Bere P., Sarraj B., Khan M.W., Pakanati K.C. (2011). Regulation of induced colonic inflammation by *Lactobacillus acidophilus* deficient in lipoteichoic acid. Proc. Natl. Acad. Sci. USA.

[168] Khan M.W., Zadeh M., Bere P., Gounaris E., Owen J., Klaenhammer T., Mohamadzadeh M. (2012). Modulating intestinal immune responses by lipoteichoic acid-deficient *Lactobacillus acidophilus*. Immunotherapy.

[169] Chen K., Luo H., Li Y., Han X., Gao C., Wang N., Lu F., Wang H. (2023). *Lactobacillus paracasei* TK1501 fermented soybeans alleviate dextran sulfate sodium-induced colitis by regulating intestinal cell function. J. Sci. Food Agric..

[170] Angelin J., Kavitha M. (2020). Exopolysaccharides from probiotic bacteria and their health potential. Int. J. Biol. Macromol..

[171] Kaur N., Dey P. (2023). Bacterial exopolysaccharides as emerging bioactive macromolecules: From fundamentals to applications. Res. Microbiol..

[172] Chaisuwan W., Jantanasakulwong K., Wangtueai S., Phimolsiripol Y., Chaiyaso T., Techapun C., Phongthai S., You S., Regenstein J.M., Seesuriyachan P. (2020). Microbial exopolysaccharides for immune enhancement: Fermentation, modifications and bioactivities. Food Biosci..

[173] Shao L., Wu Z., Zhang H., Chen W., Ai L., Guo B. (2014). Partial characterization and immunostimulatory activity of exopolysaccharides from *Lactobacillus rhamnosus* KF5. Carbohydr. Polym..

[174] Tang W., Dong M., Wang W., Han S., Rui X., Chen X., Jiang M., Zhang Q., Wu J., Li W. (2017). Structural characterization and antioxidant property of released exopolysaccharides from *Lactobacillus delbrueckii* ssp. *bulgaricus* SRFM-1. Carbohydr. Polym..

[175] Lee K., Kim H.J., Kim S.A., Park S.D., Shim J.J., Lee J.L. (2021). Exopolysaccharide from *Lactobacillus plantarum* HY7714 Protects against Skin Aging through Skin-Gut Axis Communication. Molecules.

[176] Wang X., Tian J., Zhang X., Tang N., Rui X., Zhang Q., Dong M., Li W. (2022). Characterization and Immunological Activity of Exopolysaccharide from *Lacticaseibacillus paracasei* GL1 Isolated from Tibetan Kefir Grains. Foods.

[177] Min W.H., Fang X.B., Wu T., Fang L., Liu C.L., Wang J. (2019). Characterization and antioxidant activity of an acidic exopolysaccharide from *Lactobacillus plantarum* JLAU103. J. Biosci. Bioeng..

[178] Makino S., Sato A., Goto A., Nakamura M., Ogawa M., Chiba Y., Hemmi J., Kano H., Takeda K., Okumura K. (2016). Enhanced natural killer cell activation by exopolysaccharides derived from yogurt fermented with *Lactobacillus delbrueckii* ssp. *bulgaricus OLL*1073R-1. J. Dairy Sci..

[179] Kwon M., Lee J., Park S., Kwon O.H., Seo J., Roh S. (2020). Exopolysaccharide Isolated from *Lactobacillus plantarum* L-14 Has Anti-Inflammatory Effects via the Toll-Like Receptor 4 Pathway in LPS-Induced RAW 264.7 Cells. Int. J. Mol. Sci..

[180] Khedr O.M.S., El-Sonbaty S.M., Moawed F.S.M., Kandil E.I., Abdel-Maksoud B.E. (2022). *Lactobacillus acidophilus* ATCC 4356 Exopolysaccharides Suppresses Mediators of Inflammation through the Inhibition of TLR2/STAT-3/P38-MAPK Pathway in DEN-Induced Hepatocarcinogenesis in Rats. Nutr. Cancer.

[181] Xu X., Qiao Y., Peng Q., Shi B., Dia V.P. (2022). Antioxidant and Immunomodulatory Properties of Partially purified Exopolysaccharide from *Lactobacillus casei* Isolated from Chinese Northeast Sauerkraut. Immunol. Investig..

[182] Li J., Li Q., Wu Q., Gao N., Wang Z., Yang Y., Shan A. (2023). Exopolysaccharides of *Lactobacillus rhamnosus* GG ameliorate Salmonella typhimurium-induced intestinal inflammation via the TLR4/NF-kappaB/MAPK pathway. J. Anim. Sci. Biotechnol..

[183] Kissova Z., Mudronova D., Link R., Tkacikova L. (2024). Immunomodulatory effect of probiotic exopolysaccharides in a porcine In vitro co-culture model mimicking the intestinal environment on ETEC infection. Vet. Res. Commun..

[184] Kissova Z., Tkacikova L., Mudronova D., Bhide M.R. (2022). Immunomodulatory Effect of *Lactobacillus reuteri* (*Limosilactobacillus reuteri*) and Its Exopolysaccharides Investigated on Epithelial Cell Line IPEC-J2 Challenged with *Salmonella typhimurium*. Life.

[185] Wang X., Shao C., Liu L., Guo X., Xu Y., Lu X. (2017). Optimization, partial characterization and antioxidant activity of an exopolysaccharide from *Lactobacillus plantarum* KX041. Int. J. Biol. Macromol..

[186] Wang J., Zhang J., Guo H., Cheng Q., Abbas Z., Tong Y., Yang T., Zhou Y., Zhang H., Wei X. (2023). Optimization of Exopolysaccharide Produced by *Lactobacillus plantarum* R301 and Its Antioxidant and Anti-Inflammatory Activities. Foods.

[187] Huang Y.Y., Wu J.M., Wu W.T., Lin J.W., Liang Y.T., Hong Z.Z., Jia X.Z., Liu D.M. (2022). Structural, antioxidant, and immunomodulatory activities of an acidic exopolysaccharide from *Lactiplantibacillus plantarum* DMDL 9010. Front. Nutr..

[188] Li J.Y., Jin M.M., Meng J., Gao S.M., Lu R.R. (2013). Exopolysaccharide from *Lactobacillus planterum* LP6: Antioxidation and the effect on oxidative stress. Carbohydr. Polym..

[189] Zhang L., Liu C., Li D., Zhao Y., Zhang X., Zeng X., Yang Z., Li S. (2013). Antioxidant activity of an exopolysaccharide isolated from *Lactobacillus plantarum* C88. Int. J. Biol. Macromol..

[190] Liu C.F., Tseng K.C., Chiang S.S., Lee B.H., Hsu W.H., Pan T.M. (2011). Immunomodulatory and antioxidant potential of *Lactobacillus* exopolysaccharides. J. Sci. Food Agric..

[191] Rajoka M.S.R., Mehwish H.M., Kitazawa H., Barba F.J., Berthelot L., Umair M., Zhu Q., He Z., Zhao L. (2022). Techno-functional properties and immunomodulatory potential of exopolysaccharide from *Lactiplantibacillus plantarum* MM89 isolated from human breast milk. Food Chem..

[192] Xu Y., Cui Y., Wang X., Yue F., Shan Y., Liu B., Zhou Y., Yi Y., Lu X. (2019). Purification, characterization and bioactivity of exopolysaccharides produced by *Lactobacillus plantarum* KX041. Int. J. Biol. Macromol..

[193] Zhu Y., Wang X., Pan W., Shen X., He Y., Yin H., Zhou K., Zou L., Chen S., Liu S. (2019). Exopolysaccharides produced by yogurt-texture improving *Lactobacillus plantarum* RS20D and the immunoregulatory activity. Int. J. Biol. Macromol..

[194] Yilmaz T., Simsek O. (2020). Potential Health Benefits of Ropy Exopolysaccharides Produced by *Lactobacillus plantarum*. Molecules.

[195] Ma F., Song Y., Sun M., Wang A., Jiang S., Mu G., Tuo Y. (2021). Exopolysaccharide Produced by *Lactiplantibacillus plantarum*-12 Alleviates Intestinal Inflammation and Colon Cancer Symptoms by Modulating the Gut Microbiome and Metabolites of C57BL/6 Mice Treated by Azoxymethane/Dextran Sulfate Sodium Salt. Foods.

[196] Zhou X., Qi W., Hong T., Xiong T., Gong D., Xie M., Nie S. (2018). Exopolysaccharides from *Lactobacillus plantarum* NCU116 Regulate Intestinal Barrier Function via STAT3 Signaling Pathway. J. Agric. Food Chem..

[197] Song Y., Sun M., Mu G., Tuo Y. (2024). Exopolysaccharide secreted by *Lactiplantibacillus plantarum* Y12 showed inhibitory effect on the pathogenicity of Shigella flexneri in vitro and in vivo. Int. J. Biol. Macromol..

[198] Tao T., Zhang L., Yu T., Ma J., Lu S., Ren J., Li X., Guo X. (2024). Exopolysaccharide production by *Lactobacillus plantarum* T10 is responsible for the probiotic activity in enhancing intestinal barrier function in vitro and in vivo. Food Funct..

[199] Zhang J., Zhang H., Xiao Y., Wang H., Zhang H., Lu W. (2024). Interspecific differences and mechanisms of *Lactobacillus*-derived anti-inflammatory exopolysaccharides. Int. J. Biol. Macromol..

[200] Chen L., Gu Q., Zhou T. (2022). Statistical Optimization of Novel Medium to Maximize the Yield of Exopolysaccharide From *Lacticaseibacillus rhamnosus* ZFM216 and Its Immunomodulatory Activity. Front. Nutr..

[201] Han J., Xia W., Wang D., Wang Y., Liu Z., Wu Z. (2024). Characterization of an exopolysaccharide synthesized by *Lacticaseibacillus rhamnosus* B6 and its immunomodulatory activity in vitro. Int. J. Biol. Macromol..

[202] Ciszek-Lenda M., Nowak B., Srottek M., Walczewska M., Gorska-Fraczek S., Gamian A., Marcinkiewicz J. (2013). Further studies on immunomodulatory effects of exopolysaccharide isolated from *Lactobacillus rhamnosus* KL37C. Cent. Eur. J. Immunol..

[203] Li L., Jiang Y.J., Yang X.Y., Liu Y., Wang J.Y., Man C.X. (2014). Immunoregulatory effects on Caco-2 cells and mice of exopolysaccharides isolated from *Lactobacillus acidophilus* NCFM. Food Funct..

[204] Tang Y., Dong W., Wan K., Zhang L., Li C., Zhang L., Liu N. (2015). Exopolysaccharide Produced by *Lactobacillus plantarum* Induces Maturation of Dendritic Cells in BALB/c Mice. PLoS ONE.

[205] Kumari M., Dasriya V.L., Nataraj B.H., Nagpal R., Behare P.V. (2022). *Lacticaseibacillus rhamnosus*-Derived Exopolysaccharide Attenuates D-Galactose-Induced Oxidative Stress and Inflammatory Brain Injury and Modulates Gut Microbiota in a Mouse Model. Microorganisms.

[206] Wan C., Qian W.W., Liu W., Pi X., Tang M.T., Wang X.L., Gu Q., Li P., Zhou T. (2022). Exopolysaccharide from *Lactobacillus rhamnosus* ZFM231 alleviates DSS-induced colitis in mice by regulating gut microbiota. J. Sci. Food Agric..

[207] Srutkova D., Kozakova H., Novotna T., Gorska S., Hermanova P.P., Hudcovic T., Svabova T., Sinkora M., Schwarzer M. (2023). Exopolysaccharide from *Lacticaseibacillus rhamnosus* induces IgA production in airways and alleviates allergic airway inflammation in mouse model. Eur. J. Immunol..

[208] Patten D.A., Leivers S., Chadha M.J., Maqsood M., Humphreys P.N., Laws A.P., Collett A. (2014). The structure and immunomodulatory activity on intestinal epithelial cells of the EPSs isolated from *Lactobacillus helveticus* sp. Rosyjski and *Lactobacillus acidophilus* sp. 5e2. Carbohydr. Res..

[209] Noda M., Danshiitsoodol N., Kanno K., Uchida T., Sugiyama M. (2021). The Exopolysaccharide Produced by *Lactobacillus paracasei* IJH-SONE68 Prevents and Ameliorates Inflammatory Responses in DSS-Induced Ulcerative Colitis. Microorganisms.

[210] Kissova Z., Schusterova P., Mudronova D., Novotny J., Tkacikova L. (2024). Exopolysaccharides from *Limosilactobacillus reuteri*: Their influence on In vitro activation of porcine monocyte-derived dendritic cells—Brief report. Vet. Res. Commun..

[211] Ksonzekova P., Bystricky P., Vlckova S., Patoprsty V., Pulzova L., Mudronova D., Kubaskova T., Csank T., Tkacikova L. (2016). Exopolysaccharides of Lactobacillus reuteri: Their influence on adherence of *E. coli* to epithelial cells and inflammatory response. Carbohydr. Polym..

[212] Prete R., Dell’Orco F., Sabatini G., Montagano F., Battista N., Corsetti A. (2024). Improving the Antioxidant and Anti-Inflammatory Activity of Fermented Milks with Exopolysaccharides-Producing *Lactiplantibacillus plantarum* Strains. Foods.

[213] Quach N.T., Nguyen T.T.A., Vu T.H.N., Nguyen T.T.N., Tran X.K., Chu N.H., Ta T.T.T., Chu H.H., Phi Q.T. (2024). New insight into protective effect against oxidative stress and biosynthesis of exopolysaccharides produced by *Lacticaseibacillus paracasei* NC4 from fermented eggplant. Curr. Genet..

[214] Fagan R.P., Fairweather N.F. (2014). Biogenesis and functions of bacterial S-layers. Nat. Rev. Microbiol..

[215] Zhang D., Wu M., Guo Y., Xun M., Wang W., Wu Z., Pan D. (2017). Purification of *Lactobacillus acidophilus* surface-layer protein and its immunomodulatory effects on RAW264.7 cells. J. Sci. Food Agric..

[216] Gerbino E., Carasi P., Mobili P., Serradell M.A., Gomez-Zavaglia A. (2015). Role of S-layer proteins in bacteria. World J. Microbiol. Biotechnol..

[217] Suzuki S., Yokota K., Igimi S., Kajikawa A. (2019). Comparative analysis of immunological properties of S-layer proteins isolated from *Lactobacillus* strains. Microbiology.

[218] Wang H., Zhang L., Xu S., Pan J., Zhang Q., Lu R. (2018). Surface-Layer Protein from *Lactobacillus acidophilus* NCFM Inhibits Lipopolysaccharide-Induced Inflammation through MAPK and NF-kappaB Signaling Pathways in RAW264.7 Cells. J. Agric. Food Chem..

[219] Wang H., Zhang Q., Niu Y., Zhang X., Lu R. (2019). Surface-layer protein from *Lactobacillus acidophilus* NCFM attenuates tumor necrosis factor-alpha-induced intestinal barrier dysfunction and inflammation. Int. J. Biol. Macromol..

[220] Bumgardner S.A., Zhang L., LaVoy A.S., Andre B., Frank C.B., Kajikawa A., Klaenhammer T.R., Dean G.A. (2018). Nod2 is required for antigen-specific humoral responses against antigens orally delivered using a recombinant *Lactobacillus* vaccine platform. PLoS ONE.

[221] Konstantinov S.R., Smidt H., de Vos W.M., Bruijns S.C., Singh S.K., Valence F., Molle D., Lortal S., Altermann E., Klaenhammer T.R. (2008). S layer protein A of *Lactobacillus acidophilus* NCFM regulates immature dendritic cell and T cell functions. Proc. Natl. Acad. Sci. USA.

[222] Klotz C., Goh Y.J., O’Flaherty S., Barrangou R. (2020). S-layer associated proteins contribute to the adhesive and immunomodulatory properties of *Lactobacillus acidophilus* NCFM. BMC Microbiol..

[223] Klotz C., Goh Y.J., O’Flaherty S., Johnson B., Barrangou R. (2020). Deletion of S-Layer Associated Ig-Like Domain Protein Disrupts the *Lactobacillus acidophilus* Cell Surface. Front. Microbiol..

[224] Prado Acosta M., Ruzal S.M., Cordo S.M. (2016). S-layer proteins from *Lactobacillus* sp. inhibit bacterial infection by blockage of DC-SIGN cell receptor. Int. J. Biol. Macromol..

[225] Li P., Yu Q., Ye X., Wang Z., Yang Q. (2011). Lactobacillus S-layer protein inhibition of *Salmonella*-induced reorganization of the cytoskeleton and activation of MAPK signalling pathways in Caco-2 cells. Microbiology.

[226] Li P., Yin Y., Yu Q., Yang Q. (2011). *Lactobacillus acidophilus* S-layer protein-mediated inhibition of *Salmonella*-induced apoptosis in Caco-2 cells. Biochem. Biophys. Res. Commun..

[227] Gao X., Huang L., Zhu L., Mou C., Hou Q., Yu Q. (2016). Inhibition of H9N2 Virus Invasion into Dendritic Cells by the S-Layer Protein from L. *acidophilus ATCC* 4356. Front. Cell. Infect. Microbiol..

[228] Kobatake E., Kabuki T. (2019). S-Layer Protein of *Lactobacillus helveticus* SBT2171 Promotes Human beta-Defensin 2 Expression via TLR2-JNK Signaling. Front. Microbiol..

[229] Qi S.R., Cui Y.J., Liu J.X., Luo X., Wang H.F. (2020). *Lactobacillus rhamnosus* GG components, SLP, gDNA and CpG, exert protective effects on mouse macrophages upon lipopolysaccharide challenge. Lett. Appl. Microbiol..

[230] Gao K., Wang C., Liu L., Dou X., Liu J., Yuan L., Zhang W., Wang H. (2017). Immunomodulation and signaling mechanism of *Lactobacillus rhamnosus* GG and its components on porcine intestinal epithelial cells stimulated by lipopolysaccharide. J. Microbiol. Immunol. Infect..

[231] Zhang Y., Shi X., Hao S., Lu Q., Zhang L., Han X., Lu W. (2018). Inhibition of *Shigella sonnei*-induced epithelial barrier disruption by surface-layer associated proteins of lactobacilli from Chinese fermented food. J. Dairy Sci..

[232] Zhang Y.C., Zhang L.W., Tuo Y.F., Guo C.F., Yi H.X., Li J.Y., Han X., Du M. (2010). Inhibition of *Shigella sonnei* adherence to HT-29 cells by lactobacilli from Chinese fermented food and preliminary characterization of S-layer protein involvement. Res. Microbiol..

[233] Zhang X., Li Y., Zhang C., Chi H., Liu C., Li A., Yu W. (2022). Postbiotics derived from *Lactobacillus plantarum* 1.0386 ameliorate lipopolysaccharide-induced tight junction injury via MicroRNA-200c-3p mediated activation of the MLCK-MLC pathway in Caco-2 cells. Food Funct..

[234] Liu Z., Shen T., Moyer M.P., Qin H. (2011). Identification of the *Lactobacillus* SLP domain that binds gastric mucin. Front. Biosci..

[235] Yin M., Yan X., Weng W., Yang Y., Gao R., Liu M., Pan C., Zhu Q., Li H., Wei Q. (2018). Micro Integral Membrane Protein (MIMP), a Newly Discovered Anti-Inflammatory Protein of *Lactobacillus plantarum*, Enhances the Gut Barrier and Modulates Microbiota and Inflammatory Cytokines. Cell Physiol. Biochem..

[236] Proft T., Baker E.N. (2009). Pili in Gram-negative and Gram-positive bacteria—Structure, assembly and their role in disease. Cell Mol. Life Sci..

[237] Leser T., Baker A. (2024). Molecular mechanisms of *Lacticaseibacillus rhamnosus*, LGG^®^ probiotic function. Microorganisms.

[238] von Ossowski I., Reunanen J., Satokari R., Vesterlund S., Kankainen M., Huhtinen H., Tynkkynen S., Salminen S., de Vos W.M., Palva A. (2010). Mucosal adhesion properties of the probiotic *Lactobacillus rhamnosus* GG SpaCBA and SpaFED pilin subunits. Appl. Environ. Microbiol..

[239] Lebeer S., Claes I., Tytgat H.L., Verhoeven T.L., Marien E., von Ossowski I., Reunanen J., Palva A., Vos W.M., Keersmaecker S.C. (2012). Functional analysis of *Lactobacillus rhamnosus* GG pili in relation to adhesion and immunomodulatory interactions with intestinal epithelial cells. Appl. Environ. Microbiol..

[240] von Ossowski I., Pietila T.E., Rintahaka J., Nummenmaa E., Makinen V.M., Reunanen J., Satokari R., de Vos W.M., Palva I., Palva A. (2013). Using recombinant Lactococci as an approach to dissect the immunomodulating capacity of surface piliation in probiotic *Lactobacillus rhamnosus* GG. PLoS ONE.

[241] Ganguli K., Collado M.C., Rautava J., Lu L., Satokari R., von Ossowski I., Reunanen J., de Vos W.M., Palva A., Isolauri E. (2015). *Lactobacillus rhamnosus* GG and its SpaC pilus adhesin modulate inflammatory responsiveness and TLR-related gene expression in the fetal human gut. Pediatr. Res..

[242] Tytgat H.L., Douillard F.P., Reunanen J., Rasinkangas P., Hendrickx A.P., Laine P.K., Paulin L., Satokari R., de Vos W.M. (2016). *Lactobacillus rhamnosus* GG Outcompetes *Enterococcus faecium* via Mucus-Binding Pili: Evidence for a Novel and Heterospecific Probiotic Mechanism. Appl. Environ. Microbiol..

[243] Vargas Garcia C.E., Petrova M., Claes I.J., De Boeck I., Verhoeven T.L., Dilissen E., von Ossowski I., Palva A., Bullens D.M., Vanderleyden J. (2015). Piliation of *Lactobacillus rhamnosus* GG promotes adhesion, phagocytosis, and cytokine modulation in macrophages. Appl. Environ. Microbiol..

[244] Ardita C.S., Mercante J.W., Kwon Y.M., Luo L., Crawford M.E., Powell D.N., Jones R.M., Neish A.S. (2014). Epithelial adhesion mediated by pilin SpaC is required for *Lactobacillus rhamnosus* GG-induced cellular responses. Appl. Environ. Microbiol..

[245] Tytgat H.L., van Teijlingen N.H., Sullan R.M., Douillard F.P., Rasinkangas P., Messing M., Reunanen J., Satokari R., Vanderleyden J., Dufrene Y.F. (2016). Probiotic Gut Microbiota Isolate Interacts with Dendritic Cells via Glycosylated Heterotrimeric Pili. PLoS ONE.

[246] Kurokawa K., Ryu K.H., Ichikawa R., Masuda A., Kim M.S., Lee H., Chae J.H., Shimizu T., Saitoh T., Kuwano K. (2012). Novel bacterial lipoprotein structures conserved in low-GC content gram-positive bacteria are recognized by Toll-like receptor 2. J. Biol. Chem..

[247] Nguyen M.T., Gotz F. (2016). Lipoproteins of Gram-Positive Bacteria: Key Players in the Immune Response and Virulence. Microbiol. Mol. Biol. Rev..

[248] Jin M.S., Kim S.E., Heo J.Y., Lee M.E., Kim H.M., Paik S.G., Lee H., Lee J.O. (2007). Crystal structure of the TLR1-TLR2 heterodimer induced by binding of a tri-acylated lipopeptide. Cell.

[249] Takeuchi O., Kawai T., Muhlradt P.F., Morr M., Radolf J.D., Zychlinsky A., Takeda K., Akira S. (2001). Discrimination of bacterial lipoproteins by Toll-like receptor 6. Int. Immunol..

[250] Lee I.-C., van Swam I.I., Boeren S., Vervoort J., Meijerink M., Taverne N., Starrenburg M., Bron P.A., Kleerebezem M. (2020). Lipoproteins Contribute to the Anti-inflammatory Capacity of *Lactobacillus plantarum* WCFS1. Front. Microbiol..

[251] Kim B.S., Yun C.H., Han S.H., Song K.D., Kang S.S. (2021). Inhibitory Effect of Lipoteichoic Acid Derived from Three Lactobacilli on Flagellin-Induced IL-8 Production in Porcine Peripheral Blood Mononuclear Cells. Probiotics Antimicrob. Proteins.

[252] Kim B., Shynlova O., Lye S. (2019). Probiotic *Lactobacillus rhamnosus* GR-1 is a unique prophylactic agent that suppresses infection-induced myometrial cell responses. Sci. Rep..

[253] Dalpke A., Frank J., Peter M., Heeg K. (2006). Activation of toll-like receptor 9 by DNA from different bacterial species. Infect. Immun..

[254] Rachmilewitz D., Katakura K., Karmeli F., Hayashi T., Reinus C., Rudensky B., Akira S., Takeda K., Lee J., Takabayashi K. (2004). Toll-like receptor 9 signaling mediates the anti-inflammatory effects of probiotics in murine experimental colitis. Gastroenterology.

[255] Iliev I.D., Tohno M., Kurosaki D., Shimosato T., He F., Hosoda M., Saito T., Kitazawa H. (2008). Immunostimulatory oligodeoxynucleotide containing TTTCGTTT motif from *Lactobacillus rhamnosus* GG DNA potentially suppresses OVA-specific IgE production in mice. Scand. J. Immunol..

[256] Kant R., de Vos W.M., Palva A., Satokari R. (2014). Immunostimulatory CpG motifs in the genomes of gut bacteria and their role in human health and disease. J. Med. Microbiol..

[257] Hiramatsu Y., Satho T., Hyakutake M., Irie K., Mishima K., Miake F., Kashige N. (2014). The anti-inflammatory effects of a high-frequency oligodeoxynucleotide from the genomic DNA of *Lactobacillus casei*. Int. Immunopharmacol..

[258] Bouladoux N., Hall J.A., Grainger J.R., dos Santos L.M., Kann M.G., Nagarajan V., Verthelyi D., Belkaid Y. (2012). Regulatory role of suppressive motifs from commensal DNA. Mucosal Immunol..

[259] Shigemori S., Namai F., Ogita T., Sato T., Shimosato T. (2020). Oral priming with oligodeoxynucleotide particles from *Lactobacillus rhamnosus* GG attenuates symptoms of dextran sodium sulfate-induced acute colitis in mice. Anim. Sci. J..

[260] Luyer M.D., Buurman W.A., Hadfoune M., Speelmans G., Knol J., Jacobs J.A., Dejong C.H., Vriesema A.J., Greve J.W. (2005). Strain-specific effects of probiotics on gut barrier integrity following hemorrhagic shock. Infect. Immun..

[261] Hotte N.S., Salim S.Y., Tso R.H., Albert E.J., Bach P., Walker J., Dieleman L.A., Fedorak R.N., Madsen K.L. (2012). Patients with inflammatory bowel disease exhibit dysregulated responses to microbial DNA. PLoS ONE.

[262] Dean S.N., Leary D.H., Sullivan C.J., Oh E., Walper S.A. (2019). Isolation and characterization of *Lactobacillus*-derived membrane vesicles. Sci. Rep..

[263] Hu R., Lin H., Wang M., Zhao Y., Liu H., Min Y., Yang X., Gao Y., Yang M. (2021). *Lactobacillus reuteri*-derived extracellular vesicles maintain intestinal immune homeostasis against lipopolysaccharide-induced inflammatory responses in broilers. J. Anim. Sci. Biotechnol..

[264] Pang Y., Ermann Lundberg L., Mata Forsberg M., Ahl D., Bysell H., Pallin A., Sverremark-Ekstrom E., Karlsson R., Jonsson H., Roos S. (2022). Extracellular membrane vesicles from *Limosilactobacillus reuteri* strengthen the intestinal epithelial integrity, modulate cytokine responses and antagonize activation of TRPV1. Front. Microbiol..

[265] Champagne-Jorgensen K., Mian M.F., McVey Neufeld K.A., Stanisz A.M., Bienenstock J. (2021). Membrane vesicles of *Lacticaseibacillus rhamnosus* JB-1 contain immunomodulatory lipoteichoic acid and are endocytosed by intestinal epithelial cells. Sci. Rep..

[266] Miyoshi Y., Saika A., Nagatake T., Matsunaga A., Kunisawa J., Katakura Y., Yamasaki-Yashiki S. (2021). Mechanisms underlying enhanced IgA production in Peyer’s patch cells by membrane vesicles derived from *Lactobacillus sakei*. Biosci. Biotechnol. Biochem..

[267] Kurata A., Kiyohara S., Imai T., Yamasaki-Yashiki S., Zaima N., Moriyama T., Kishimoto N., Uegaki K. (2022). Characterization of extracellular vesicles from *Lactiplantibacillus plantarum*. Sci. Rep..

[268] Yamasaki-Yashiki S., Miyoshi Y., Nakayama T., Kunisawa J., Katakura Y. (2019). IgA-enhancing effects of membrane vesicles derived from *Lactobacillus sakei* subsp. *sakei* NBRC15893. Biosci. Microbiota Food Health.

[269] Champagne-Jorgensen K., Jose T.A., Stanisz A.M., Mian M.F., Hynes A.P., Bienenstock J. (2021). Bacterial membrane vesicles and phages in blood after consumption of *Lacticaseibacillus rhamnosus* JB-1. Gut Microbes.

[270] Muller L., Kuhn T., Koch M., Fuhrmann G. (2021). Stimulation of Probiotic Bacteria Induces Release of Membrane Vesicles with Augmented Anti-inflammatory Activity. ACS Appl. Bio Mater..

[271] Zhou X., Gao M., De X., Sun T., Bai Z., Luo J., Wang F., Ge J. (2023). Bacterium-like particles derived from probiotics: Progress, challenges and prospects. Front. Immunol..

[272] Raya Tonetti F., Arce L., Salva S., Alvarez S., Takahashi H., Kitazawa H., Vizoso-Pinto M.G., Villena J. (2020). Immunomodulatory Properties of Bacterium-Like Particles Obtained From Immunobiotic Lactobacilli: Prospects for Their Use as Mucosal Adjuvants. Front. Immunol..

[273] Guo Z., Ren H., Chang Q., Liu R., Zhou X., Xue K., Sun T., Luo J., Wang F., Ge J. (2024). Lactobacilli-derived adjuvants combined with immunoinformatics-driven multi-epitope antigens based approach protects against *Clostridium perfringens* in a mouse model. Int. J. Biol. Macromol..

[274] Shida K., Kiyoshima-Shibata J., Kaji R., Nagaoka M., Nanno M. (2009). Peptidoglycan from lactobacilli inhibits interleukin—12 production by macrophages induced by *Lactobacillus casei* through Toll—like receptor 2—dependent and independent mechanisms. Immunology.

[275] Brott A.S., Clarke A.J. (2019). Peptidoglycan O-acetylation as a virulence factor: Its effect on lysozyme in the innate immune system. Antibiotics.

[276] Sychantha D., Brott A.S., Jones C.S., Clarke A.J. (2018). Mechanistic pathways for peptidoglycan O-acetylation and de-O-acetylation. Front. Microbiol..

[277] Kang S.-S., Sim J.-R., Yun C.-H., Han S.H. (2016). Lipoteichoic acids as a major virulence factor causing inflammatory responses via Toll-like receptor 2. Arch. Pharmacal Res..

[278] Hidalgo-Cantabrana C., López P., Gueimonde M., de Los Reyes-Gavilán C.G., Suárez A., Margolles A., Ruas-Madiedo P. (2012). Immune modulation capability of exopolysaccharides synthesised by lactic acid bacteria and bifidobacteria. Probiotics Antimicrob. Proteins.

[279] Caggianiello G., Kleerebezem M., Spano G. (2016). Exopolysaccharides produced by lactic acid bacteria: From health-promoting benefits to stress tolerance mechanisms. Appl. Microbiol. Biotechnol..

[280] Konieczna P., Schiavi E., Ziegler M., Groeger D., Healy S., Grant R., O’Mahony L. (2015). Human dendritic cell DC-SIGN and TLR-2 mediate complementary immune regulatory activities in response to *Lactobacillus rhamnosus* JB-1. PLoS ONE.

[281] Prado Acosta M., Geoghegan E.M., Lepenies B., Ruzal S., Kielian M., Martinez M.G. (2019). Surface (S) layer proteins of *Lactobacillus acidophilus* block virus infection via DC-SIGN interaction. Front. Microbiol..

[282] Basset A., Zhang F., Benes C., Sayeed S., Herd M., Thompson C., Golenbock D.T., Camilli A., Malley R. (2013). Toll-like receptor (TLR) 2 mediates inflammatory responses to oligomerized RrgA pneumococcal pilus type 1 protein. J. Biol. Chem..

[283] Bertorello S., Cei F., Fink D., Niccolai E., Amedei A. (2024). The future exploring of gut microbiome-immunity interactions: From in vivo/vitro models to in silico innovations. Microorganisms.

[284] Metwaly A., Kriaa A., Hassani Z., Carraturo F., Druart C., Arnauts K., Wilmes P., Walter J., Rosshart S. (2025). A Consensus Statement on establishing causality, therapeutic applications and the use of preclinical models in microbiome research. Nat. Rev. Gastroenterol. Hepatol..

[285] Griffin M.E., Hang H.C. (2022). Microbial mechanisms to improve immune checkpoint blockade responsiveness. Neoplasia.

[286] Hajishengallis G., Lambris J.D. (2016). More than complementing Tolls: Complement–Toll—like receptor synergy and crosstalk in innate immunity and inflammation. Immunol. Rev..

[287] Sanz Y., Moya-Pérez A. (2014). Microbiota, inflammation and obesity. Microbial Endocrinology: The Microbiota-Gut-Brain Axis in Health and Disease.

[288] Struijk E.A., Heraclides A., Witte D.R., Soedamah-Muthu S.S., Geleijnse J.M., Toft U., Lau C.J. (2013). Dairy product intake in relation to glucose regulation indices and risk of type 2 diabetes. Nutr. Metab. Cardiovasc. Dis..

[289] Mozaffarian D., Hao T., Rimm E.B., Willett W.C., Hu F.B. (2011). Changes in diet and lifestyle and long-term weight gain in women and men. N. Engl. J. Med..

[290] Wang H., Livingston K.A., Fox C.S., Meigs J.B., Jacques P.F. (2013). Yogurt consumption is associated with better diet quality and metabolic profile in American men and women. Nutr. Res..

[291] Cai J., Zhao C., Du Y., Zhang Y., Zhao M., Zhao Q. (2018). Comparative efficacy and tolerability of probiotics for antibiotic-associated diarrhea: Systematic review with network meta-analysis. United Eur. Gastroenterol. J..

[292] Huang I.F., Lin I.C., Liu P.F., Cheng M.F., Liu Y.C., Hsieh Y.D., Chen J.J., Chen C.L., Chang H.W., Shu C.W. (2015). *Lactobacillus acidophilus* attenuates *Salmonella*-induced intestinal inflammation via TGF-beta signaling. BMC Microbiol..

[293] Rokana N., Singh R., Mallappa R.H., Batish V.K., Grover S. (2016). Modulation of intestinal barrier function to ameliorate *Salmonella* infection in mice by oral administration of fermented milks produced with *Lactobacillus plantarum* MTCC 5690—A probiotic strain of Indian gut origin. J. Med. Microbiol..

[294] Goldenberg J.Z., Yap C., Lytvyn L., Lo C.K., Beardsley J., Mertz D., Johnston B.C. (2017). Probiotics for the prevention of *Clostridium difficile*-associated diarrhea in adults and children. Cochrane Database Syst. Rev..

[295] Francavilla R., Lionetti E., Castellaneta S.P., Magista A.M., Maurogiovanni G., Bucci N., De Canio A., Indrio F., Cavallo L., Ierardi E. (2008). Inhibition of *Helicobacter pylori* infection in humans by *Lactobacillus reuteri* ATCC 55730 and effect on eradication therapy: A pilot study. Helicobacter.

[296] Tongtawee T., Dechsukhum C., Leeanansaksiri W., Kaewpitoon S., Kaewpitoon N., Loyd R.A., Matrakool L., Panpimanmas S. (2015). Improved *Helicobacter pylori* Eradication Rate of Tailored Triple Therapy by Adding *Lactobacillus delbrueckii* and *Streptococcus thermophilus* in Northeast Region of Thailand: A Prospective Randomized Controlled Clinical Trial. Gastroenterol. Res. Pr..

[297] Kwon H.K., Kim G.C., Kim Y., Hwang W., Jash A., Sahoo A., Kim J.E., Nam J.H., Im S.H. (2013). Amelioration of experimental autoimmune encephalomyelitis by probiotic mixture is mediated by a shift in T helper cell immune response. Clin. Immunol..

[298] Ji H.F., Shen L. (2021). Probiotics as potential therapeutic options for Alzheimer’s disease. Appl. Microbiol. Biotechnol..

[299] Thakkar A., Vora A., Kaur G., Akhtar J. (2023). Dysbiosis and Alzheimer’s disease: Role of probiotics, prebiotics and synbiotics. Naunyn Schmiedebergs Arch. Pharmacol..

[300] Gazerani P. (2019). Probiotics for Parkinson’s Disease. Int. J. Mol. Sci..

[301] Partty A., Kalliomaki M., Wacklin P., Salminen S., Isolauri E. (2015). A possible link between early probiotic intervention and the risk of neuropsychiatric disorders later in childhood: A randomized trial. Pediatr. Res..

[302] Vogel D.Y., Glim J.E., Stavenuiter A.W., Breur M., Heijnen P., Amor S., Dijkstra C.D., Beelen R.H. (2014). Human macrophage polarization In vitro: Maturation and activation methods compared. Immunobiology.

[303] Shapouri-Moghaddam A., Mohammadian S., Vazini H., Taghadosi M., Esmaeili S.A., Mardani F., Seifi B., Mohammadi A., Afshari J.T., Sahebkar A. (2018). Macrophage plasticity, polarization, and function in health and disease. J. Cell Physiol..

[304] Carol M., Borruel N., Antolin M., Llopis M., Casellas F., Guarner F., Malagelada J.R. (2006). Modulation of apoptosis in intestinal lymphocytes by a probiotic bacteria in Crohn’s disease. J. Leukoc. Biol..

[305] Li C., Peng K., Xiao S., Long Y., Yu Q. (2023). The role of *Lactobacillus* in inflammatory bowel disease: From actualities to prospects. Cell Death Discov..

[306] Kim H.G., Lee S.Y., Kim N.R., Lee H.Y., Ko M.Y., Jung B.J., Kim C.M., Lee J.M., Park J.H., Han S.H. (2011). *Lactobacillus plantarum* lipoteichoic acid down-regulated *Shigella flexneri* peptidoglycan-induced inflammation. Mol. Immunol..

[307] Hao H., Zhang X., Tong L., Liu Q., Liang X., Bu Y., Gong P., Liu T., Zhang L., Xia Y. (2021). Effect of Extracellular Vesicles Derived From *Lactobacillus plantarum* Q7 on Gut Microbiota and Ulcerative Colitis in Mice. Front. Immunol..

[308] Riehl T.E., Alvarado D., Ee X., Zuckerman A., Foster L., Kapoor V., Thotala D., Ciorba M.A., Stenson W.F. (2019). *Lactobacillus rhamnosus* GG protects the intestinal epithelium from radiation injury through release of lipoteichoic acid, macrophage activation and the migration of mesenchymal stem cells. Gut.

[309] Nowak B., Srottek M., Ciszek-Lenda M., Skalkowska A., Gamian A., Gorska S., Marcinkiewicz J. (2020). Exopolysaccharide from *Lactobacillus rhamnosus* KL37 Inhibits T Cell-dependent Immune Response in Mice. Arch. Immunol. Ther. Exp..

[310] Noda M., Sultana N., Hayashi I., Fukamachi M., Sugiyama M. (2019). Exopolysaccharide Produced by *Lactobacillus paracasei* IJH-SONE68 Prevents and Improves the Picryl Chloride-Induced Contact Dermatitis. Molecules.

[311] Kawanabe-Matsuda H., Takeda K., Nakamura M., Makino S., Karasaki T., Kakimi K., Nishimukai M., Ohno T., Omi J., Kano K. (2022). Dietary *Lactobacillus*-Derived Exopolysaccharide Enhances Immune-Checkpoint Blockade Therapy. Cancer Discov..

[312] Lu S., Xu J., Zhao Z., Guo Y., Zhang H., Jurutka P.W., Huang D., Cao C., Cheng S. (2023). Dietary *Lactobacillus rhamnosus* GG extracellular vesicles enhance antiprogrammed cell death 1 (anti-PD-1) immunotherapy efficacy against colorectal cancer. Food Funct..

[313] Bender M.J., McPherson A.C., Phelps C.M., Pandey S.P., Laughlin C.R., Shapira J.H., Medina Sanchez L., Rana M., Richie T.G., Mims T.S. (2023). Dietary tryptophan metabolite released by intratumoral *Lactobacillus reuteri* facilitates immune checkpoint inhibitor treatment. Cell.

[314] Wang C.H., Yen H.R., Lu W.L., Ho H.H., Lin W.Y., Kuo Y.W., Huang Y.Y., Tsai S.Y., Lin H.C. (2022). Adjuvant Probiotics of *Lactobacillus salivarius* subsp. *salicinius* AP-32, *L. johnsonii* MH-68, and *Bifidobacterium animalis* subsp. *lactis* CP-9 Attenuate Glycemic Levels and Inflammatory Cytokines in Patients With Type 1 Diabetes Mellitus. Front. Endocrinol..

[315] Gu Y., Chen H., Li X., Li D., Sun Y., Yang L., Ma Y., Chan E.C.Y. (2023). *Lactobacillus paracasei* IMC 502 ameliorates type 2 diabetes by mediating gut microbiota-SCFA-hormone/inflammation pathway in mice. J. Sci. Food Agric..

[316] Chiu Y.H., Tsai J.J., Lin S.L., Lin M.Y. (2014). *Lactobacillus casei* MYL01 modulates the proinflammatory state induced by ethanol in an In vitro model. J. Dairy Sci..

[317] Nimgampalle M., Kuna Y. (2017). Anti-Alzheimer Properties of Probiotic, *Lactobacillus plantarum* MTCC 1325 in Alzheimer’s Disease induced Albino Rats. J. Clin. Diagn. Res..

[318] Del Piano M., Balzarini M., Carmagnola S., Pagliarulo M., Tari R., Nicola S., Deidda F., Pane M. (2014). Assessment of the capability of a gelling complex made of tara gum and the exopolysaccharides produced by the microorganism *Streptococcus thermophilus* ST10 to prospectively restore the gut physiological barrier: A pilot study. J. Clin. Gastroenterol..

