# Supplementary material for: Lactobacilli-Derived Microbe-Associated Molecular Patterns (MAMPs) in Host Immune Modulation
Source: Biomolecules. 2025 Nov 17;15(11):1609. doi: 10.3390/biom15111609 (PMC12650587; doi:10.3390/biom15111609)
Supplement: Supplementary file 1 [file biomolecules-15-01609-s001.zip › biomolecules-3960741-supplementary.pdf]

Table S1. Summary of immunomodulatory effects induced by Lactobacillus-derived MAMPs in *in vitro*, *ex vivo*, and *in vivo* models. The table reports a wide range of host immune responses to PGN, LTA, EPS, SLP, and other MAMPs from various Lactobacillus species and strains. Observed effects include modulation of cytokine production, activation or inhibition of signalling pathways (e.g., NF-κB, MAPK), expression of surface markers, and enhancement of innate and adaptive immune responses. Both pro- and anti-inflammatory activities are highlighted depending on the MAMP type, dosage, and host model.

Species/strain	MAMP	Model of study	Effects	Ref
Livestock faces-derived *Lactobacillus* spp.	PGN	*Ex vivo*: BALB/c mice-extracted PMs and splenocytes<br><br>PGN-treated Once or Thrice (acute or chronic treatment)	*Acute treatment*<br>- TIRAP, TOLLIP, Akt1↓<br>- IRAK-1, p38 ↑<br>- IL-1, IL6, CD80, CD86, IL-12↑<br>- iNOS =, NO =<br>- Class II MHC ↑<br>- Vcam1, Icam2, Icam4 ↑<br>- CD3 ↑<br>- T_h1 $CD4^+$ T-cell ↑<br><br>*Chronic treatment*:<br>- TLR-2 ↑<br>- TIRAP ↓<br>- p38, TOLLIP, TRAF-6, NF-κB1/2 ↑<br>- IL-1, IL6, CD80, CD86, TNF-α ↑<br>- TGF ↑<br>- iNOS =, NO =<br>- Vcam1, Icam2 ↑<br>- CD3, T_h1 $CD4^+$ T-cell, IFN-γ ↑<br>- IL-18 receptor ↑<br>- Jak, STAT ↑<br>- IL-10 and IL-10 receptor ↑	[1]
L. acidophilus,<br>*L. rhamnosus*	PGN	*In vitro:* LPS-induced RAW264.7 macrophages	*Anti-inflammatory effects:*<br>- TLR-4↓<br>- IL-6, IL-1β, TNF-α ↓	[2]

L. acidophilus	PGN	*In vitro:* LPS-induced RAW264.7 macrophages	*Anti-inflammatory effects:*<br>- iNOS activity ↓<br>- COX-2 activity ↓	[3]
L. acidophilus	PGN-derived fragment (GMTP)	*In vitro:* unstimulated mouse BMDMs	*Immunostimulatory effects:*<br>- IL-1β, TNF-α, KC, IFN-γ, CXCL10 ↑<br>- COX-2 ↑<br>- Phosphorylation of IRF5, cJUN, p65-NF-κB ↑	[4]
L. acidophilus KLDS 1.0738	PGN	*In vitro*: unstimulated BALB/c mice-derived PMs	*Immmunostimulatory effects:*<br>- TLR-2, NF-κB ↑<br>- IFN-γ, TGF-β, IL-10 ↑,	[5]
		In vivo: β-LG-stimulated BALB/c mice allergy models	*Anti-allergic effects:*<br>- TLR-2, NF-κB ↑<br>- Serum IgE ↓<br>- Foxp3, CD25 ↑<br>- TGF-β ↑<br>- IL-17, IL-6, IL-23 ↓<br>- RORγT ↓<br>- T_{reg}/T_h17 regulation	
L. rhamnosus CRL1505	PGN	*In vitro:* human unstimulated moDCs,	*Immunostimulatory effects*:<br>- CD86 ↑<br>- TNF-α ↑	[6]
		In vitro: human LPS-stimulated moDCs,	*Anti-inflammatory effects:*<br>- HLA-DR, CD80, CD83 ↓<br>- TNF-α, IL-10 ↑	

		In vitro: IECs co-cultured with unstimulated moDCs,	*Immunostimulatory effects*:<br>- CD86 ↑<br>- IL-8 ↑	
		In vitro: IECs co-cultured with LPS-stimulated moDCs,	*Immunostimulatory effects*:<br>- CD86 ↑<br>- TNF-α, IL-1β, IL-10 ↑	
L. rhamnosus CRL1505	PGN	*In vivo:*<br>*S. pneumoniae*-infected Swiss-albino malnourished mice	*Resistance to infection:*<br>- BAL leucocytes, macrophages, neutrophils ↑<br>- IL-6, IL-1β, TNF-α, IL-10 ↑<br>- TLR-2 and TLR-9 ↑<br>- Blood Peroxidase activity ↑	[7]
L. rhamnosus CRL1505	PGN	*In vivo:*<br>*S. pneumoniae*-infected Swiss-albino malnourished mice	*Resistance to infection:*<br>- BAL leucocytes, macrophages, neutrophils ↑<br>- Blood leucocytes ↓<br>- Blood neutrophils =<br>- Peroxidase activity ↑<br>- Spleen macrophages ↑<br>- BAL and blood TNF-α =; IL-10 ↑<br>- BAL and blood<br>- Lung and thymus total lymphocytes ↑; Spleen total lymphocytes =<br>- Lung, thymus and spleen $CD4^+$ T-cells ↑; Lung and thymus $CD8^+$ T-cells ↑<br>- Spleen $CD8^+$ T-cells =<br>- BAL IFN-γ ↓; Serum IFN-γ =<br>- BAL and serum IL-2, IL-4 and IL-10 ↑<br>- Lung, spleen and bone marrow mature B-cells ↑<br>- Lung and bone marrow immature B-cells ↑	[8]

			- Spleen immature B-cells (B220^{low} CD19^{+} CD24^{high} cells) =<br>- Spleen immature B-cells (IgD^{+} IgM^{+} CD24^{low} cells) ↑<br>- BAL and serum IgA, IgG and IgM ↑	
L. rhamnosus CRL1505	PGN	*In vivo*: Poly-(I:C)-stimulated BALB/c mice	*Immunomodulatory effects:*<br>- BAL IFNs (-α, -β, -γ), TNF-α, IL-10 ↑<br>- BAL IL-6 ↓<br>- BAL and blood leukocytes ↑<br>- Myeloperoxidase activity ↑	[9]
		In vitro: Poly(I:C)-stimulated RAW264.7 macrophages	- TNF-α, IL-1β, IL-6, IL-10 ↑	
L. rhamnosus CRL1505<br>*L. rhamnosus* IBL027	PGN	*In vivo: S. pneumoniae*-infected Poly (I:C)-stimulated BALB/c mice models	*Resistance to infection:*<br>- Lung and blood count ↓<br>- Albumin ↓ (only CRL1505)<br>- LDH ↓<br>- BAL IFN-γ, IFN-β ↑<br>- BAL IL-10 ↑ (only CRL1505)	[10]
		In vivo: RSV-infected *S. pneumoniae*-superinfected BALB/c mice models	*Resistance to infection:*<br>- Lung viral titer and pneumococcal count ↓ (only CRL1505)<br>- Negative hemocultures (only CRL1505)	
		Ex vivo: RSV-infected AMs from BALB/c mice	*Immunomodulatory effects:*<br>*CRL1505 PGN-treated mice*<br>- IL-6, IFN-α, IFN-β, IFN-γ, IL-27, OAS1, RNAseL gene expression ↑<br>- TNF-α, IL-1α, IL-1β gene expression ↓<br><br>*IBL027 PGN-treated mice*<br>- IFN-α, IFN-β, IFN-γ, Mx1, OAS1 gene expression ↑<br>- TNF-α, IL-1α, IL-1β, IL-6 gene expression =	

		Ex vivo: *S. pneumoniae*-infected AMs from RSV-challenged BALB/c mice	*Immunomodulatory effects:*<br>*For both strains:*<br>- IL-6, IFN-α, IFN-β, IFN-γ, IL-27, TNF-α, IL-1α, IL-1β CCL2, CXCL2, IL-10 gene expression ↑	
L. rhamnosus MLGA	PGN	*In vitro:* unstimulated broiler chickens PBMCs	*Defensin production and immunomodulatory effects*:<br>- B-defensin 9 ↑<br>- IL-12p40 =	[11]
		In vitro: unstimulated broiler chickens splenocytes	- B-defensin 9 ↑<br>- IL-12p40 =<br>- IL-1β, IL-8 ↓	
	Lysozyme-hydrolysed PGN fragments	*In vitro:* unstimulated broiler chickens PBMCs	- B-defensin 9 ↑<br>- IL-1β, IL-12p40 ↓	
		In vitro: unstimulated broiler chickens splenocytes	- B-defensin 9 =<br>- IL-1β, IL-8 ↓	
L. reuteri	PGN	*In vitro*: LPS-stimulated RAW264.7 macrophages	*Anti-inflammatory effects:*<br>- IL-1β, IL-6, CCL20 ↓<br>- PI3K/Akt, p38, JNK and ERK phosphorylation ↓<br>- IκB restored, NF-κB pathway ↓<br>- TLR-2 and TLR-4 ↓	[12]
	MDP		- IL-1β, IL-6, CCL20 ↓	
L. plantarum CAU1055	PGN	*In vitro:* LPS-stimulated RAW264.7 macrophages	*Anti-inflammatory effects:*<br>- NO, TNF-α, IL-6 ↓<br>- iNOS, COX-2 ↓	[13]

		In vivo: DSS-induced ICR mice models	*Protective effects:*<br>- Mucosal damage and immune cells infiltration ↓<br>- TNF-α, IL-6 ↓<br>- iNOS, COX-2 ↓	
L. plantarum ATCC 14917<br>*L. rhamnosus* GG (ATCC 53103)	PGN	*In vitro:* Poly (I:C)-stimulated HT-29 cell line	*Anti-inflammatory effect (L. plantarum):*<br>- IL-8 ↓<br>*No significative effect (LGG):*<br>- IL-8 =	[14]
L. casei Shirota	PSPGs	*In vitro:*<br>LPMCs from LPS-stimulated BALB/c mice;<br>LPS-stimulated RAW264.7 macrophages;<br>Human PBMCs from UC patients	*Anti-inflammatory effects:*<br>- IL-6 ↓<br>- NF-κB phosphorylation ↓	[15]
L. casei Shirota<br>PSPG-I negative *L. casei*		*In vivo:* Ileitis BALB/c mice	*Protective effects (L. casei Shirota, compared to PSPG-I negative L. casei):*<br>- Ileal mucosa IL-6 ↓<br>- Ileal mucosa SOC3 ↓<br>- Milder ileitis	
L. casei Shirota<br>*L. johnsonii* JCM2012<br>*L. plantarum* ATCC14917	PGN	*In vitro:* PMs from *L. casei*-stimulated BALB/c mice	*L. plantarum and L. johnsonii PGN*:<br>- IL-12 ↓<br>- TNF-α =<br><br>*L. plantarum and L. johnsonii PGN:*<br>- IL-12p40 ↓<br>- IL-12p35 =	[16]

L. salivarius Ls33	PGN	*In vivo:* TNBS-induced colitis BALB/c and C57BL/6J mice models (NOD-2$^+$ and NOD-2$^-$)	*Less anti-inflammatory effects in NOD-2$^-$ mice:*<br>- Body weight loss, macroscopic inflammation ↑<br>*More anti-inflammatory effects in NOD-2$^+$ mice:*<br>- Body weight loss, macroscopic inflammation ↓<br>- Colonic IL-1β, CXCL2, IL-6, TNF-α, *IL-10, gene expression ↓<br>- Colonic, splenic and MLN *IL-10 ↑<br>- IFN-γ and IDO gene expression ↑<br>- Regulatory CD11c$^+$ CD103$^+$ DCs and CD4$^+$ FoxP3$^+$ T_{reg} in MLN ↑<br><br>*IL-10 discrepancy between mRNA and protein probably due to faster kinetics of gene expression in PGN-treated mice.	[17]
L. casei<br>*L. paracasei*<br>*L. reuteri*<br>*L. rhamnosus*	PGH	*In vivo:* TNBS-induced colitis C57BL/6 mice models	*Anti-inflammatory effects:*<br>- NOD-2 ligands from PGN-digestion ↑<br>- Colon tissues TNF-α, IFN-γ, IL-6 ↓<br>- Colon tissues IL-10 ↑	[18]
ΔLc-p75 *L. casei* BL23	p75 PGH	*In vitro*: unstimulated human moDCs	*Decreased immunostimulatory activity*:<br>- Il-8, CD83, CD80, CD86, HLA-DQ ↑ in both WT and ΔLc-p75<br>- IL-6↓, IL-1β↓, TNF-α ↓, IL-10 ↓, IL-12 ↓, IL-23 ↓ in ΔLc-p75 compared to WT<br>- Reduced phagocytosis of mutated *L. casei* compared to WT	[19]
		In vitro: human moDCs – PBLs co-culture	- IFN-γ-secreting T-cells and IL-17A-secreting T-cells ↓ in ΔLc-p75 *L. casei* compared to WT	
L. plantarum K8 (KCTC10887BP)	LTA	*In vitro*: Pam2CSK4-stimulated Caco-2 cell line	*Anti-inflammatory effects*:<br>- IL-8↓ (LTA de-alanylation o de-acylation inhibited this effect)<br>- CD25 ↓	[20]

			- p38 and JNK phosphorylation ↓<br>- ERK phosphorylation =<br>- IκBα degradation ↓	
L. plantarum K8 (KCTC10887BP)	LTA<br>PGN	*In vitro*: flexPGN-stimulated THP-1 cell line	*Anti-inflammatory effects (only LTA, PGN ineffective)*:<br>- IL-1β, TNF-α ↓<br>- NOD2 ↓<br>- ERK1/2, JNK1/2, p38 phosphorylation ↓<br>- IκBα degradation ↓<br>- NOD-2 ↓	[21]
L. plantarum K8 (KCTC10887BP)	LTA	*In vitro*: LPS-stimulated THP-1 cell line	*Anti-inflammatory effects:*<br>- IL-8, TNF-α ↓<br>- COX-2, Bax ↓<br>- HSP27, ERK, p38, JNK phosphorylation ↓<br>- IκBα degradation ↓<br>- TLR-4, CCR7 ↓<br>- Adhesion to HUVEC cell line ↓	[22]
		In vitro: LPS-stimulated RAW264.7 macrophages	- TNF-α ↓<br>- iNOS, COX-2 ↓	
		In vivo: HFHC-treated ApoE negative mice	*Anti-atherosclerotic effects:*<br>- Monocyte/macrophage infiltration ↓<br>- MMP-9 ↓	
L. plantarum K8 (KCTC10887BP)	LTA	*In vitro*: IFN-γ-stimulated RAW264.7 macrophages	*Enhancement of IFN-γ-induced immune-stimulation*:<br>- NO ↑, iNOS ↑<br>- IFN-β ↑ (at high LTA dose)<br>- STAT1 ↑<br>- NF-κB activation ↑	[23]

		In vitro: IFN-γ-stimulated BMDMs from BALB/c mice (WT or TLR-2-deficient)	- NO higher in WT compared to TLR-2-deficient	
L. plantarum K8	LTA	*In vitro:* TNF-α or IFN-γ-stimulated HaCaT human keratinocytes cell line	*Anti-complement effect:*<br>- C3 and C4 complement components ↓<br>- C2, C5 =	[24]
		In vitro: LPS-stimulated THP-1 macrophages and IL-1β-stimulated HepG2 hepatocytes	- C3 complement component ↓	
		In vivo: TNF-α or IFN-γ-stimulated BALB/c mice	- NF-κB p65 and p38 MAPK activation ↓<br>- STAT-1, JAK-1 phosphorylation ↓<br>- Serum C3 ↓<br>- MAC formation/activity ↓	
L. plantarum K8	- LTA<br>- De-alanylated or de-acylated LTA<br>- PGN<br>- Lipoproteins	*In vitro:* Poly-(I:C)-stimulated IPEC-J2	*Anti-inflammatory effects (only LTA):*<br>- IL-8 ↓<br>- ERK and p38 phosphorylation ↓<br>- IκBα degradation ↓<br><br>De-alanylated or de acyated LTA, PGN and lipoproteins didn’t reduce IL-8 levels	[25]
L. delbrueckii<br>*L. sakei*<br>*L. rhamnosus* GG	LTA		- IL-8 ↑ (only *L. delbrueckii* LTA)<br><br>LGG and L. sakei LTA didn’t reduce IL-8 levels	
L. plantarum L-137<br>*L. plantarum JCM1149*	LTA	*In vitro:* unstimulated murine splenocytes	*Immunostimulatory effects:*<br>IL-6, IL-10, TNF-α, IL-12p40 ↑ (both strains)	[26]
L. plantarum CRL1506 WT	LTA	*In vitro:* Poly-(I:C)-stimulated PIE cells	*Wild type:*<br>- IFN-β ↑<br>- IL-6, CCL2 ↓	[27]

L. plantarum<br>CRL1506 DltD<br>negative			*DltD negative:*<br>- IFN-β ↑ same as WT<br>- IL-6, CCL2 ↑ compared to WT	
		In vivo: Poly-(I:C)-stimulated BALB/c mice	*Wild type*:<br>- Weight loss, LDH, AST ↓<br>- Reduced neutrophils in blood<br>- Intestine and serum IFN-β, IFN-γ, IL-10 ↑<br>- Intestinal fluid and serum TNF-α, IL-6, IL15 ↓<br>- $CD3^+$ $NK1.1^+$ and $CD3^+CD8\alpha\alpha^+$ IELs ↓<br>- NKG2D and RAE1 ↓<br><br>*DltD negative:*<br>- Weight loss, AST ↓ (weakly)<br>- No reduction of LDH<br>- No reduction of neutrophils in blood<br>- Intestine and serum IFN-β, IFN-γ ↑<br>- Intestinal fluid IL-15 ↓ (weakly)<br>- No reduction of TNF-α, IL-6, IL-10 and serum IL-15<br>- No reduction of $CD3^+CD8\alpha\alpha^+$ IELs<br>- $CD3^+$ $NK1.1^+$ IELs, NKG2D and RAE1 ↓ (weakly)	
L. rhamnosus KL37C	LTA	*In vitro*:<br>Unstimulated-PM from CBA/J mice<br>Unstimulated BMDC from BALB/c mice<br>Zymosan-stimulated Mice neutrophils	*Immunostimulatory effects*:<br>- ROS ↓ in zymosan-stimulated neutrophils<br>- TNF-α, IL-6, IL-12p40, IL-10 ↑ in PMs<br>- IL-12p40 ↑, IL-6 =, IL-10 = in BMDC<br>- COX-2 ↑, PGE_2 ↑ in PMs	[28]

	EPS	*In vitro*:<br>Unstimulated-PM from CBA/J mice<br>Unstimulated BMDC from BALB/c mice<br>Zymosan-stimulated Mice neutrophils	- ROS ↓ in zymosan-stimulated neutrophils<br>- TNF-α, IL-6, IL-12p40, IL-10 = in PMs<br>- PMs phagocytic activity =<br>- COX-2 =, PGE_2 = in PMs<br>- IL-12p40 =, IL-6 =, IL-10 = in BMDC	
		In vivo: mice models	*Anti-allergic effects*:<br>- Anti-OVA IgG ↓, IgG1 ↓, IgG2 ↓ in mice serum	
L. rhamnosus GG	LTA	*In vitro:* unstimulated BMDCs from C57BL/6	*Immunostimulatory effects:*<br>- MHC type II, CD86, IL-1β, IL-6, TNF-α, IL-10 ↑<br>- IL-12p70, ILp40 =	[29]
		In vitro: Co-culture of BMDC from C57BL/6 and CD-4$^+$ T-cells from OT-II	- CD-4$^+$ T-cells proliferation ↑<br>- IL-17A ↑	
		Ex vivo: SI, PPs and MLNs from BALB/c mice models	*Small intestine:*<br>- IL-1β, IL-6, TNF-α, IL-10 ↑<br>*Peyer Patches:*<br>- IL-12p35, TNF-α, IFN-γ ↑<br>- CD11c$^+$ MHC type II (IA/IE)^{high} DCs ↓ (probably due to migration)<br>- CD103$^+$ DCs ↑<br>*Mesenteric Lymph Nodes:*<br>- IL-12p35, IFN-γ ↑	
L. rhamnosus GG	LTA	*In vivo*: Radiation-treated C57BL/6J	*Radioprotective effects:*<br>- Crypt survival ↑<br>- Epithelial stem cells protection<br>- CXCL12 ↑ by pericryptal macrophages<br>- COX-2 activity ↑ and PGE_2 ↑ in MSCs	[30]

		In vitro: unstimulated BMDCs from C57BL/6 mice and BALB/c mice	*NCK2025*:<br>- MHC type II, CD40, CD80, CD86 ↓<br>- TLR-2 =<br>- TNF-α, IL-12 ↑ (weakly)<br>- IL-10 ↑<br>*NCK56*:<br>- TLR-2 ↑<br>- TNF-α, IL-12 ↑<br>- IL-10 ↑ (weakly)	
L. acidophilus NCK56 (NCFM)<br>*L. acidophilus* NCK2025 (LTA-deficient strain)	LTA	*In vitro:* Co-culture of *L. acidophilus*-treated BMDCs and MLN-derived T-cells from mice models	*NCK2025*:<br>- T-cell proliferation abrogated compared to NCK56<br>- IL-10 ↑<br>- IFN-γ, IL-2, TNF-α ↑ (weakly)	[31]
		In vivo: DSS-induced colitis C57BL/6 mice models	*NCK2025*:<br>- Weight loss, diarrhea, hemoccult positivity, DAI score ↓<br>- IL-10 ↑<br>- T_{reg}s ↑ compared to NCK56 treatment<br>*NCK56*:<br>- Did not prevent colitis onset<br>- IL-6, IL-12, TNF-α, IFN-γ, IL-1β ↑ compared to NCK2025<br>- CCL11, iNOS, CD45, TIMP1, TNFR, VCAM1, ICAM1, FASL, CD40, COX-2 ↑ compared to NCK2025 treatment	
L. acidophilus NCK56 (NCFM)<br>*L. acidophilus* NCK2025 (LTA-deficient strain)	LTA	*Ex vivo:* $CD11c^+$ DCs and $F4/80^+$ macrophages from *L. acidophilus* treated C57BL/6 mice	*NCK2025*:<br>- IL-10 ↑ compared to NCK56, but delayed production in DCs<br>- TNF-α, IL-12 ↓ compared to NCK56 treatment<br>- T_{reg}s, $CD4^+$ $IL\text{-}10^+$ T-cells ↑<br>- IFN-γ ↓ compared to NCK56 treatment	[32]

			NCK56:<br>- TNF-α, IL-12, IFN-γ ↑	
L. casei BL23<br>*L. casei* BL580 (DltD negative, no LTA D-alanylation)<br>*L. casei* BL180 (RecA negative)	LTA	*In vivo:* DSS-induced colitis BALB/c mice models	*Protective effects (BL23)*:<br>- DAI score, weight loss, diarrhoea, intestinal inflammation ↓<br>- Stool consistency ↑<br>- IL-1α, IL-6, IL-17, G-CSF, MCP-1, KC ↓<br><br>*BL580 and BL180:*<br>- Less protective then BL23<br>- IL-6, IL-17 and KC ↓ (BL580)	[33]
L. paracasei TK1501	LTA	*In vitro:* unstimulated RAW264.7 macrophages	*Immunostimulatory effects*:<br>- IL-1β, IL-5 ↑<br>- IL-4, IL-10, TNF-α =<br>- Clearance LPS ↑	[34]
		In vivo: DSS-induced colitis BALB/c mice models	*Protective, anti-inflammatory effects:*<br>- DAI score, weight loss, intestinal inflammation ↓<br>- Serum IL-1β, colonic IFN-γ ↓<br>- Serum IL-4, colonic TGF-β ↑<br>- Colonic macrophages ↓	
	PGN	*In vivo:* DSS-induced colitis BALB/c mice models	*Protective, anti-inflammatory effects:*<br>- Weight loss, intestinal inflammation, DAI score ↓<br>- Serum TNF-α, colonic IFN-γ ↓<br>- TGF-β ↑	
L. plantarum WCFS1 WT	LTA	*In vitro:* unstimulated BMDCs from BALB/c mice	*Mutant strain, anti-inflammatory effects:*<br>- TLR2 heterodimers activation ↓<br>- TNF-α =<br>- IL-10 ↑	[35]

L. plantarum WCFS1 DltX-D negative (no LTA D-alanylation)	*Ex vivo:* Various cells from BALB/c mice	*Peyer's patches:*<br>- DCs frequency ↓ (only mutant strain compared to WT)<br>- $CD103^+$ DCs frequencies = (both strains compared to control)<br>- DC activation = (both strains compared to control)<br>- Early activated $CD4^+$ T cells frequency ↑ (both strains compared to control)<br>- Balance $FoxP3^+$/$CD25^+$ $FoxP3^-$ $CD4^+$ T cells ↓ (only mutant compared to WT)<br><br>*Small intestinal lamina propria:*<br>- $CD103^+$ DCs frequencies ↑ (both strains compared to control)<br>- DC activation = (both strains compared to control)<br>- Early activated $CD4^+$ T cells frequency = (both strains compared to control)<br>- Balance $FoxP3^+$/$CD25^+$ $FoxP3^-$ $CD4^+$ T cells ↑ (only WT compared to control)<br><br>*Large intestinal lamina propria:*<br>- Early activated $CD4^+$ T cells frequency ↑ (only WT compared to control)<br>- Early activated $CD8^+$ T cells frequency ↑ (only WT compared to control)<br>- $FoxP3^+$ $CD4^+$ T cells ↑ (only WT compared to control)<br>- Balance $FoxP3^+$/$CD25^+$ $FoxP3^-$ $CD4^+$ T cells not significative increase trend (only WT compared to control)<br><br>Spleen<br>- $CD103^+$ DCs ↑ (only WT)<br>- $CD80^+$ DCs =

			- CD8$^+$ T cells ↓ (both strains compared to control)<br>- FoxP3$^+$ CD4$^+$ T cells ↑ (only WT compared to control)<br>- CD25$^+$ FoxP3$^-$ CD4$^+$ T cells ↑ (only mutant compared to control)<br>- IFN-γ^+ T_h1 cells ↓ (only mutant compared to control)<br>- IL-10$^+$ T_{reg}s and IL-10$^+$ CD8$^+$ T-cells ↑ (only WT compared to control)<br>- IFN-γ^+ CD8$^+$ T-cells ↑ (only WT compared to mutant and control)<br>- IL-17$^+$ CD8$^+$ T-cells ↑ (both strains compared to control)<br><br>MLNs<br>- CD80$^+$ DCs ↑ (only WT compared to mutant)<br>- CD103$^+$ DCs not significative increase trend (only WT compared to control)<br>- CD25$^+$ FoxP3$^-$ CD4$^+$ T cells ↓ (only WT compared to control)<br>- FoxP3$^+$ CD4$^+$ T cells = (both strains compared to control)<br>- IL-5$^+$ T_H2 cells and IL-5$^+$ CD8$^+$ T-cells ↓ (both strains compared to control)<br>- IFN-γ^+ CD8$^+$ T-cells ↑ (both strains compared to control)	
L. rhamnosus GG	spaC pilin	*In vitro*: IL-1β-stimulated foetal human IECs H4 cell line	*Anti-inflammatory effects:*<br>- IL-6 ↓	[36]
		In vitro: TNF-α-stimulated foetal human IECs H4 cell line	*Pro-inflammatory effects:*<br>- IL-6 ↑	

		In vitro: unstimulated foetal human IECs H4 cell line	*Immunomodulatory effects:*<br>- TLR-3, TLR-4, TIRAP ↓<br>- IL-10, TOLLIP =<br>- IL-1RN ↑	
L. rhamnosus GG	spaCBA pilus	*In vitro*: immature unstimulated moDCs	*Immunostimulatory effects*:<br>- IL-6, IL-10, IL-12p40, IL-12p35 ↑	[37]
L. rhamnosus GG<br>*L. rhamnosus* CMPG5357 (spaCBA negative)<br>*L. rhamnosus* CMPG5351 (welE negative, pili are more exposed)	spaCBA pilus	*In vitro:* unstimulated IECs	*CMPG5357 (compared to LGG):*<br>- IL-8, TNF ↑<br>- IL-10 ↓<br>*CMPG5351 (compared to LGG):*<br>- IL-8 ↓	[38]
L. rhamnosus GG	spaC pilin<br>EPS<br>LTA	*In vitro:* unstimulated Caco-2 cell line	- IL-8 ↑ (only for LTA)	
L. plantarum C88	EPS	*Biochemical assays*	*Antioxidant effects:*<br>- Hydroxyl radical scavenging capacity<br>- DPPH free radical scavenging capacity	[39]
		In vitro: H_2O_2-treated Caco-2 cell line	- MDA levels ↓<br>- T-AOC ↑<br>- SOD activity ↑	
L. plantarum KX041	EPS	*Biochemical assays*	*Antioxidant effects:*<br>- Hydroxyl radical scavenging capacity<br>- Superoxide anion scavenging capacity<br>- ABTS radical scavenging capacity<br>- DPPH free radical scavenging capacity<br>- Reducing power	[40]
L. plantarum DMDL9010	EPS	*In vitro:* H_2O_2-treated RAW264.7 macrophages	*Antioxidant and anti-inflammatory effects:*<br>- ROS, MDA, LDH ↓<br>- SOD, CAT, GSH-Px activities ↑	[41]

			- GSH ↑<br>- NO, IL-6, TNF-α ↓<br>- ERK, JNK, p38, IκBα, NF-κB p65 à phosphorylation ↓	
L. plantarum NCU116	EPS	*In vivo:* DSS-induced colitis C57BL/6 mice models	*Protective and anti-inflammatory effects*:<br>- Weight loss, colon shortening, colon weight, DAI score, histopathological damage ↓<br>- Claudin 1, occludin, ZO-1 ↑<br>- TNF-α, IFN-γ, IL-6 ↓	[42]
		In vitro: unstimulated Caco-2 cell line	- TEER ↑<br>- Occludin, ZO-1 ↑<br>- STAT-3 expression and phosphorylation ↑	
L. plantarum HY7714	EPS	*In vitro:* TNF-α-stimulated *Caco*-2 cell line	*Protective and anti-inflammatory effects:*<br>- ZO-1, OCL-1 ↑<br>- IL-1β, IL-6 ↓<br>- MMP1, MMP2 ↓	[43]
		In vitro: UVB-irradiated HS68 dermic fibroblast cell line	*UVB-protective and antioxidant effects:*<br>- MMP1, MMP3 ↓<br>- HAS1, 2, 3, SPT1 ↑<br>- TNF-α, IL-1β, IL-6, IL-13 ↓<br>- UVB-cytotoxicity ↓<br>- ROS production ↓	
L. plantarum L-14	EPS	*In vitro:* LPS-stimulated RAW264.7 macrophages	*Anti-inflammatory effects:*<br>- IL-6, TNF-α, IL-1β ↓<br>- COX-2, iNOS ↓<br>- NF-κB phosphorylation and translocation ↓<br>- JNK, ERK and p38 phosphorylation ↓<br>- NRF2, HO-1 ↑<br>- TLR-4, MyD88 ↓	[44]
L. plantarum MM89	EPS	*In vitro:* unstimulated RAW264.7 macrophages	*Immunostimulatory effects:*<br>- Phagocytic activity ↑<br>- IL-10, IL-6, IL-1β, TNF-α ↑	[45]
		In vivo: immunosuppressed BALB/c mice	- Splenic lymphocytes proliferation ↑<br>- Intestinal sIgA levels ↑	

			- Serum IL-2, TNF-α ↑	
L. rhamnosus LOCK900	EPS	*In vivo and ex-vivo:* BALB/c mice	*Immunostimulatory effects*:<br>- BAL neutrophils, lymphocytes and monocytes/macrophages levels ↑<br>- Neutrophils, DCs, T-cells, FoxP3$^+$ T_{reg}s ↑ in lung tissues<br>- MHC type II ↑<br>- BAL and lung cell cultures TGF-β1 ↑<br>- B-cells percentage of CD45$^+$ cells in lung tissue↓<br>- IgA$^+$ B-cells in lung tissue ↑<br>- BAL and lung cell cultures IgA ↑	[46]
		In vivo and ex-vivo: OVA/Alum mixture-sensitized BALB/c mice	*Anti-allergic effects*:<br>- OVA-specific IgG in serum =<br>- OVA-specific and total IgE in serum and lungs =<br>- IL-4, IL-5, IL13 ↓ in splenocytes culture, lungs and BAL<br>- BAL and lung cell cultures total IgA, OVA-specific and bacteria-specific IgA ↑<br>- TGF-β1 in lung tissues ↑<br>- B-cells percentage of CD45$^+$ cells in lung tissue ↑	
L. rhamnosus NCDC710	EPS	*In vivo:* D-galactose-treated Swiss albino mice	*Protective and antioxidant effects:*<br>- Positive effects on brain and colon histopathology<br>- SOD, CAT, GSH-Px, T-AOC in brain ↑<br>- MDA, NO in brain ↓<br>- TNF-α, IL-6, IL-1β, MCP-1, lipofuscin in brain ↓<br>- IL-10 in brain ↑<br>- ZO-11, Claudin-1 and occludin ↑<br>- NF-κB p65, COX-2, iNOS ↓	[47]

L. rhamnosus GG	EPS	*In vivo: Salmonella typhimurium*-infected BALB/c mice	*Protective effects:*<br>- Body weight loss, diarrhoea, histological damage, crypt depth ↓<br>- Kidney, spleen and liver indices* ↓<br>*Anti-inflammatory and antioxidant effects:*<br>- IL-1β, IL-2, IL-6, TNF-α ↓<br>- IL-4 ↑<br>- H_2O_2, MDA ↓<br>- SOD, CAT ↑<br>- NF-κB p65 nuclear translocation, IκB phosphorylation and degradation ↓<br>- Phosphorylation of ERK and JNK ↓<br><br>*in this paper, organ indices are calculated as follows: $\frac{\text{fresh weight of organ}}{\text{body live weight}} \times 100\%$	[48]
L. acidophilus ATCC4356	EPS	*Ex vivo:* DEN-treated Swiss albino rats	*Anti-inflammatory, antioxidant effects:*<br>- ALT, MDA, γ-GT ↓<br>- GSH ↑<br>- IL-17, TGF-β1, TLR-2 ↓<br>- IL-10 ↑<br>- STAT-3 and p38 ↓	[49]
L. acidophilus NCFM	EPS	*In vitro:* unstimulated Caco-2 cell line<br>*In vivo*: BALB/c mice	*Immunostimulatory effects:*<br>- IL-1α, CCL2, TNF-α, PTX3 ↑ (both *in vivo* and *in vitro*)<br>- Mononuclear phagocyte system promoted in mice	[50]
L. casei NA-2	EPS	*Biochemical assays*	*Antioxidant effects:*<br>- Weak hydroxyl radical scavenging capacity<br>- Good superoxide anion scavenging capacity<br>- Good DPPH free radical scavenging capacity	[51]

		In vitro: unstimulated RAW 264.7	*Immunostimulatory effects:*<br>- Phagocytic activity ↑<br>- NO, IL-6 =<br>- TNF-α ↑<br>- ROS ↑<br>- NF-κB p65, c-jun ↑	
		In vitro: LPS-stimulated RAW 264.7	*Anti-inflammatory effects:*<br>- NO, iNOS ↓	
L. delbrueckii ssp. *Bulgaricus* OLL1073R-1	EPS	*In vitro:* C3H/HeJ mice-derived spleen cells<br><br>Cells treated with EPS	*Immunostimulatory effects:*<br>- IFN-γ, IL-6, IL-10 ↑	[52]
		Ex vivo: C3H/HeJ mice derived spleen cells<br><br>Mice treated with EPS	*Immunostimulatory effects:*<br>- IFN-γ ↑<br>- NK cell activity ↑	
		Ex vivo: IFN-γ KO and MyD88 KO BALB/c mice	- NK cell activity =	
L. helveticus LZ-R-5	EPS	*In vitro:* unstimulated RAW264.7 macrophages	*Immunostimulatory effects*:<br>- Macrophage proliferation and phagocytosis ↑<br>- Acid phosphatase activity ↑<br>- NO, TNF-α, IL-6, IL-1β, IL-10 ↑	[53]
L. paracasei IJH-SONE68	EPS mixture	*In vitro: Salmonella typhimurium* or *Campylobacter jejuni*-stimulated Caco-2 cell line	*Immunostimulatory effects:*<br>- IL-8 ↓	[54]
	Acidic EPS<br>Neutral EPS	*In vivo:* DSS-induced colitis C57BL/6J mice models	*Anti-inflammatory and protective effects:*<br>- Stool consistency, DAI score, colon shortening ↓ (significative only for acidic EPS)<br>- Crypt depth ↑ (both acidic and neutral EPS)<br>- Colon tissue MIP-2 ↓ (both acidic and neutral EPS)<br>- Colon tissue IL-10 ↑ (significative only for acidic EPS)<br>- COX-2, iNOS = (both acidic and neutral EPS)	

L. reuteri L26	EPS	*In vitro: Salmonella typhimurium*-stimulated IPEC-J2 cell line	*Anti-inflammatory effects:*<br>- IL-8, IL-6 ↓<br>- TNF-α =<br>- TGF-β ↓<br>- TLR-4, TLR-5 ↓	[55]
L. reuteri L26<br>*L. reuteri* DSM17938	EPS	*In vitro:* unstimulated immature porcine moDCs	*Immunostimulatory, DCs' maturation induction effects*:<br>- IL-1β, IL-6 ↑ (only DSM17938 EPS)<br>- IL-12p35 and IL-10 ↑ (both strains' EPS)<br>- TGF-β = (both strains' EPS)<br>- CD80/86, MHC type II ↑ (both strains' EPS)	[56]
L. reuteri L26	EPS	*In vitro:* unstimulated IPEC-J2	*Weak immunostimulatory effects:*<br>- NF-κB ↑<br>- TLR-5 ↓	[57]
		In vitro: unstimulated moDCs	*Weakly immunostimulatory effects:*<br>- IL-6, TLR-4, TLR-5, MyD88 ↑	
		In vitro: ETEC-stimulated IPEC-J2	*Anti-inflammatory effects:*<br>- IL-1β, IL-6, IL-12p35, TNF-α, MyD88, TLR-5, NF-κB ↓	
		In vitro: ETEC-stimulated moDCs	*Anti-inflammatory effects*<br>- IL-1β, MyD88, TLR-4, TLR-5, NF-κB ↓	
		In vitro: ETEC-stimulated IPEC-J2 + unstimulated moDCs co-culture	*Anti-inflammatory effects on moDCs:*<br>- IL-1β, IL-6 ↓<br>*Anti-inflammatory effects on IPEC-J2:*<br>- IL-12p35 ↓	
L. reuteri DSM 17938<br>*L. reuteri* L26	EPS	*In vitro:* unstimulated IPEC-1 cell line	*Weak immunostimulatory effects (DSM17938 EPS):*<br>- IL-1β ↑<br>- NF-κB, TNF-α, IL-6 =<br>*Weak immunostimulatory effects (L26):*<br>- IL-1β =<br>- NF-κB, TNF-α, IL-6 ↑	[58]

		In vitro: ETEC-stimulated IPEC-1 cell line	*Anti-inflammatory (DSM17938 EPS):*<br>- NF-κB, IL-1β, TNF-α, IL-6 ↓<br><br>*Anti-inflammatory (L26):*<br>- IL-1β, IL-6 ↓<br>- NF-κB, TNF-α =<br><br>*Adherence impairing:*<br>- Both strains inhibit ETEC adherence to IPEC-1 monolayer	
L. acidophilus ATCC 4356	SLP	*In vitro:* unstimulated murine DCs	*Immunostimulatory effects, DCs maturation:*<br>- CD80, CD86, IL-10 ↑<br>- CD40, TNF-α =	[59]
		In vitro: H9N2-treated murine DCs	*Anti-viral effects:*<br>- Number of infected DCs ↓<br>- HA, NA, PB1, NP ↓<br>- CD80, CD86, CD40, ISGs ↑<br>- IL-10 ↑<br>- TNF-α, IFN-γ ↓	
L. acidophilus NCFM<br>*L. acidophilus* NCK2439<br>*L. acidophilus* NCK2441<br>*L. acidophilus* NCK2530<br>*L. acidophilus* NCK2608<br><br>(All NCFM SLAP deletion mutant)	SLAP	*In vitro:* unstimulated murine DCs	*Immunosuppressive effects (of deletion mutants compared to NCFM):*<br>- IL-10 ↑ (NCK2439, NCK2441, NCK2608)<br>- IL-10 = (NCK2530)<br>- IL-12 ↓ (all strains)<br>- IL-6 ↑ (NCK2441, NCK2530)<br>- IL-6 = (NCK2439, NCK2608)<br>- TNF-α ↓ (NCK2441, NCK2608)<br>- TNF-α = (NCK2439, NCK2530)	[60]

L. acidophilus NCFM (SlpA dominant)<br>*L. acidophilus* NCK1377-CI (SlpA negative, SlpB dominant)	SlpA	*In vitro:* moDCs<br>Different DC/bacterium ratios tested: 1:10, 1:100, 1:1000.	*Immunostimulatory effects:*<br>- *IL-10 ↑ (both strains, NCFM more potent at 1:10 ratio)*<br>- *IL-12p70, IL-1β, TNF-α ↑ (both strains, but NCK1377-CI more potent at lower ratios)*<br>- *IL-6 ↑ (both strains, NCFM more potent at all ratios)*<br>- *CD86 ↑, DCs maturation (both strains)*	[61]
		In vitro: NCFM or NCK1377-CI-matured moDC + naïve T-cell co-culture	*NCFM-matured moDC:*<br>- IL-4 producing T-cells ↑ (T_h2 differentiation)<br>*NCK1377-CI-matured moDC:*<br>- Both IL-4 and IFN-γ producing T-cells ↑ (mixed T_h1/T_h2 response)	
L. acidophilus NCFM	SLP	*In vitro:* unstimulated Caco-2 cells	- TEER ↑<br>- Paracellular permeability ↓	[62]
		In vitro: TNF-α stimulated Caco-2 cells	*Tight junctions' protective effects:*<br>- ZO-1 and occludin ↑<br>- Claudin 1 ↓<br>*Anti-inflammatory effects:*<br>- IL-8 ↓<br>- NF-κB p65 translocation ↓	
L. acidophilus ATCC4356	SLP	*In vitro: Salmonella typhimurium*-stimulated Caco-2 cell line	*Infection protective effects:*<br>- S. *typhimurium* adhesion ↓<br>- F-actin alteration weakened<br>- TEER ↑<br>- IL-8 ↓<br>- JNK activation, p38 phosphorylation ↓	[63]
L. plantarum 1.0386	SLP	*In vitro:* LPS-stimulated Caco-2 cells	*Protective effects:*<br>- TEER ↑<br>- Paracellular permeability ↓<br>- IL-8, TNF-α ↓<br>- miRNA-200b and c ↑<br>- ZO-1, Occludin, claudin-1 ↑	[64]

			- NF-κB p-p65, MLCK, pMLC ↓	
L. plantarum	MIMP	*In vivo:* DSS-induced colitis C57BL/6 mice models	*Protective and anti-inflammatory effects:*<br>- Body weight loss, colon shortening, DAI score ↓<br>- Colon tissues IFN-γ, IL-17, IL-23 ↓<br>- Colon tissues IL-4, IL-10 ↑<br>- Colon tissues JAM-1, Occludin, ZO-1 ↑	[65]
		In vitro: PBMCs and Caco-2 cells co-culture (both treated with LPS)	*Anti-inflammatory effects:*<br>- IFN-γ, IL-17, IL-23 ↓<br>- IL-4, IL-10 ↑	
		In vitro: LPS-stimulated Caco-2 cells	*Anti-inflammatory effects*<br>- NF-κB, MAPK, JNK pathways ↓<br>- H3 and H4 histone acetylation ↓	
L. plantarum WCFS1, Lgt-deficient	Lipoproteins	*In vitro:* unstimulated human PBMCs	*Immunostimulatory effects:*<br>- IL-12, TNF-α, IL-1β, IL-8 ↑<br>- IL-10 ↓	[66]
L. plantarum KCTC10887BP<br>*L. casei* KCTC3260<br>*L. rhamnosus* GG	LTA<br>Lipoproteins	*In vitro:* flagellin-stimulated porcine PBMCs	*Anti-inflammatory effects:*<br>- IL-8 ↓ (LTA of all three strains)<br>- IL-8 = (Lipoproteins of all three strains)	[67]
L. rhamnosus GG	ODG-containing particles<br><br>(Carbonate apatite-based particles)	*In vivo:* DSS-induced colitis C57BL/6 mice	*Protective and anti-inflammatory effects:*<br>- Body weight loss, colon shortening, DAI score ↓<br>ODG only or particles only couldn't give didn't get any significative improvement	[68]
L. rhamnosus GG	SLP<br>gDNA<br>CpG-ODN<br>EPS	*In vitro:* LPS-stimulated IPEC-J2	*SLP, EPS*:<br>- IL-6, IL-12, TNF-α ↓<br>- p38 phosphorylation, NF-κB p65 phosphorylation ↓<br>- IκBα levels ↑	[69]

			- p-ERK ↑ (only SLP)<br>*Genomic DNA*<br>- IL-6, IL-12, TNF- *α* =<br>- p38 phosphorylation, NF-κB p65 phosphorylation, IκB*α* levels =<br>*CpG-ODN*<br>- IL-12, TNF-*α* ↑,<br>- IL-6 =<br>- p38 phosphorylation, IκB*α* levels =<br>- NF-κB p65 phosphorylation ↑	
L. rhamnosus LMG P-22799<br>*L. fermentum* NumRes2	DNA	*In vitro:* endotoxin-stimulated RAW264.7 macrophages	*Anti-inflammatory effects:*<br>- TNF-*α* ↓ (both strains)	[70]
L. rhamnosus GG	SLP<br>gDNA<br>SLP + gDNA<br>CpG-ODN<br>SLP + CpG-ODN	*In vitro:* LPS-stimulated RAW264.7 macrophages	*TNF-α*<br>- SLP, SLP + gDNA → TNF-*α* ↓<br>- gDNA, SLP + CpG-ODN → TNF-*α* =<br>- CpG-ODN → TNF-*α* ↑<br>*IL-6*<br>- All components and combinations → IL-6 ↓<br>*IL-10*<br>- SLP + gDNA → IL-10 ↓<br>- All the other components and combinations → IL-10 =<br>*TLR-2:*<br>- SLP, gDNA, SLP + gDNA → TLR-2 ↓<br>- CpG-ODN, SLP + CpG-ODN → TLR-2 =<br>*TLR-4:*<br>- SLP, gDNA, SLP + gDNA, SLP + CpG-ODN→ TLR-4 ↓<br>- CpG-ODN → TLR-4 =<br>*TLR-9:*<br>- SLP, SLP + CpG-ODN → TLR-9 ↓	[71]

			- gDNA, SLP + gDNA, CpG-ODN → TLR-9 =<br><br>*ERK phosphorylation:*<br>- All components and combinations à pERK ↓	
L. rhamnosus JB-1	EVs	*In vitro:* TNF-stimulated T84 intestinal epithelial cell line	*Anti-inflammatory effects:*<br>- IL-8 ↓	[72]
		In vitro: unstimulated BMDCs	*Anti-inflammatory effects:*<br>- IL-10 ↓	
L. reuteri DSM17938	MVs	*In vitro:* unstimulated PBMCs	*Immunostimulatory effects:*<br>- IL-6, IL-10 ↑<br>- IFN-γ, IL-17A à not detected	[73]
L. reuteri BBC3	EVs	*In vivo:* LPS-treated broiler chickens	*Protective and anti-inflammatory effects:*<br>- Weight loss, food intake reduction, intestinal injury ↓<br>- Villus height ↑<br>- Crypt depth ↓<br>- TNF-α, IL-1β, IL-6, IL-8, IL-17, MIP-1β ↓<br>- IL-10, TGF-β ↑<br>- MPO activity ↓	[74]

		In vitro: LPS-stimulated HD11 Mφ cell line	*Anti-inflammatory effects:*<br>- NF-κB activity ↓<br>- TNF-α, IL-1β, IL-6 ↓<br>- IL-10, TGF-β ↑	
		In vitro: Splenic lymphocytes from LPS-treated chickens + HD11 Mφ co-culture<br><br>(HD11 Mφ are EVs-treated)	*Splenic lymphocytes effects:*<br>- IL-10, TGF-β, CD25, CTLA-4, LAG-3 ↑<br>- IFN-γ, IL-17 ↓<br>- IL-4 =	
L. reuteri DSM17938<br>*L. reuteri* DSM32846	MVs	*In vitro*: ETEC-infected Caco-2/ HT29-MTX mixed cell culture	*Protective effects:*<br>- TEER ↑<br>- Paracellular permeability ↓	[75]
		In vitro: unstimulated PBMCs	*Immunostimulatory effects:*<br>- IL-6, IL-1β ↑	
		In vitro: S. aureus CFS-stimulated PBMCs	*Anti-inflammatory effects:*<br>- IFN-γ, TNF-α ↓	
L. plantarum<br>NBRC 15891	MVs	*In vitro:* LPS-stimulated HT29 cell line	*Anti-inflammatory effects:*<br>- IL-8 ↓	[76]
		In vivo: DSS-induced colitis mice models	*Protective effects:*<br>- Weight loss, colon shortening, DAI score ↓<br>- Lamina propria neutrophils ↓	

References

1. Sun, J.; Shi, Y.H.; Le, G.W.; Ma, X.Y. Distinct immune response induced by peptidoglycan derived from Lactobacillus sp. *World J Gastroenterol* **2005**, *11*, 6330-6337, doi:10.3748/wjg.v11.i40.6330.

2. Wu, Z.; Pan, D.; Guo, Y.; Sun, Y.; Zeng, X. Peptidoglycan diversity and anti-inflammatory capacity in Lactobacillus strains. *Carbohydr Polym* **2015**, *128*, 130-137, doi:10.1016/j.carbpol.2015.04.026.
3. Wu, Z.; Pan, D.D.; Guo, Y.; Zeng, X. Structure and anti-inflammatory capacity of peptidoglycan from Lactobacillus acidophilus in RAW-264.7 cells. *Carbohydr Polym* **2013**, *96*, 466-473, doi:10.1016/j.carbpol.2013.04.028.
4. Bersch, K.L.; DeMeester, K.E.; Zagani, R.; Chen, S.; Wodzanowski, K.A.; Liu, S.; Mashayekh, S.; Reinecker, H.C.; Grimes, C.L. Bacterial Peptidoglycan Fragments Differentially Regulate Innate Immune Signaling. *ACS Cent Sci* **2021**, *7*, 688-696, doi:10.1021/acscentsci.1c00200.
5. Li, A.L.; Sun, Y.Q.; Du, P.; Meng, X.C.; Guo, L.; Li, S.; Zhang, C. The Effect of Lactobacillus actobacillus Peptidoglycan on Bovine beta-Lactoglobulin-Sensitized Mice via TLR2/NF-kappaB Pathway. *Iran J Allergy Asthma Immunol* **2017**, *16*, 147-158.
6. Salva, S.; Tiscornia, I.; Gutierrez, F.; Alvarez, S.; Bollati-Fogolin, M. Lactobacillus rhamnosus postbiotic-induced immunomodulation as safer alternative to the use of live bacteria. *Cytokine* **2021**, *146*, 155631, doi:10.1016/j.cyto.2021.155631.
7. Kolling, Y.; Salva, S.; Villena, J.; Marranzino, G.; Alvarez, S. Non-viable immunobiotic Lactobacillus rhamnosus CRL1505 and its peptidoglycan improve systemic and respiratory innate immune response during recovery of immunocompromised-malnourished mice. *Int Immunopharmacol* **2015**, *25*, 474-484, doi:10.1016/j.intimp.2015.02.006.
8. Kolling, Y.; Salva, S.; Villena, J.; Alvarez, S. Are the immunomodulatory properties of Lactobacillus rhamnosus CRL1505 peptidoglycan common for all Lactobacilli during respiratory infection in malnourished mice? *PLoS One* **2018**, *13*, e0194034, doi:10.1371/journal.pone.0194034.
9. Zelaya, H.; Arellano-Arriagada, L.; Fukuyama, K.; Matsumoto, K.; Marranzino, G.; Namai, F.; Salva, S.; Alvarez, S.; Aguero, G.; Kitazawa, H.; et al. Lacticaseibacillus rhamnosus CRL1505 Peptidoglycan Modulates the Inflammation-Coagulation Response Triggered by Poly(I:C) in the Respiratory Tract. *Int J Mol Sci* **2023**, *24*, doi:10.3390/ijms242316907.
10. Raya Tonetti, F.; Clua, P.; Fukuyama, K.; Marcial, G.; Sacur, J.; Marranzino, G.; Tomokiyo, M.; Vizoso-Pinto, G.; Garcia-Cancino, A.; Kurata, S.; et al. The Ability of Postimmunobiotics from L. rhamnosus CRL1505 to Protect against Respiratory Syncytial Virus and Pneumococcal Super-Infection Is a Strain-Dependent Characteristic. *Microorganisms* **2022**, *10*, doi:10.3390/microorganisms10112185.
11. Huang, J.; Li, J.; Li, Q.; Li, L.; Zhu, N.; Xiong, X.; Li, G. Peptidoglycan derived from Lactobacillus rhamnosus MLGA up-regulates the expression of chicken beta-defensin 9 without triggering an inflammatory response. *Innate Immun* **2020**, *26*, 733-745, doi:10.1177/1753425920949917.
12. Kim, D.; Choi, H.; Oh, H.; Lee, J.; Hwang, Y.; Kang, S.S. Mutanolysin-Digested Peptidoglycan of Lactobacillus reuteri Promotes the Inhibition of Porphyromonas gingivalis Lipopolysaccharide-Induced Inflammatory Responses through the Regulation of Signaling Cascades via TLR4 Suppression. *Int J Mol Sci* **2023**, *25*, doi:10.3390/ijms25010042.
13. Choi, S.H.; Lee, S.H.; Kim, M.G.; Lee, H.J.; Kim, G.B. Lactobacillus plantarum CAU1055 ameliorates inflammation in lipopolysaccharide-induced RAW264.7 cells and a dextran sulfate sodium-induced colitis animal model. *J Dairy Sci* **2019**, *102*, 6718-6725, doi:10.3168/jds.2018-16197.

14. Park, S.W.; Choi, Y.H.; Gho, J.Y.; Kang, G.A.; Kang, S.S. Synergistic Inhibitory Effect of Lactobacillus Cell Lysates and Butyrate on Poly I:C-Induced IL-8 Production in Human Intestinal Epithelial Cells. *Probiotics Antimicrob Proteins* **2024**, *16*, 1-12, doi:10.1007/s12602-023-10042-0.
15. Matsumoto, S.; Hara, T.; Nagaoka, M.; Mike, A.; Mitsuyama, K.; Sako, T.; Yamamoto, M.; Kado, S.; Takada, T. A component of polysaccharide peptidoglycan complex on Lactobacillus induced an improvement of murine model of inflammatory bowel disease and colitis-associated cancer. *Immunology* **2009**, *128*, e170-180, doi:10.1111/j.1365-2567.2008.02942.x.
16. Shida, K.; Kiyoshima-Shibata, J.; Kaji, R.; Nagaoka, M.; Nanno, M. Peptidoglycan from lactobacilli inhibits interleukin-12 production by macrophages induced by Lactobacillus casei through Toll-like receptor 2-dependent and independent mechanisms. *Immunology* **2009**, *128*, e858-869, doi:10.1111/j.1365-2567.2009.03095.x.
17. Macho Fernandez, E.; Valenti, V.; Rockel, C.; Hermann, C.; Pot, B.; Boneca, I.G.; Grangette, C. Anti-inflammatory capacity of selected lactobacilli in experimental colitis is driven by NOD2-mediated recognition of a specific peptidoglycan-derived muropeptide. *Gut* **2011**, *60*, 1050-1059, doi:10.1136/gut.2010.232918.
18. Gao, J.; Wang, L.; Jiang, J.; Xu, Q.; Zeng, N.; Lu, B.; Yuan, P.; Sun, K.; Zhou, H.; He, X. A probiotic bi-functional peptidoglycan hydrolase sheds NOD2 ligands to regulate gut homeostasis in female mice. *Nat Commun* **2023**, *14*, 3338, doi:10.1038/s41467-023-38950-3.
19. Toth, M.; Muzsai, S.; Regulski, K.; Szendi-Szatmari, T.; Czimmerer, Z.; Rajnavolgyi, E.; Chapot-Chartier, M.P.; Bacsi, A. The Phagocytosis of Lacticaseibacillus casei and Its Immunomodulatory Properties on Human Monocyte-Derived Dendritic Cells Depend on the Expression of Lc-p75, a Bacterial Peptidoglycan Hydrolase. *Int J Mol Sci* **2022**, *23*, doi:10.3390/ijms23147620.
20. Noh, S.Y.; Kang, S.S.; Yun, C.H.; Han, S.H. Lipoteichoic acid from Lactobacillus plantarum inhibits Pam2CSK4-induced IL-8 production in human intestinal epithelial cells. *Mol Immunol* **2015**, *64*, 183-189, doi:10.1016/j.molimm.2014.11.014.
21. Kim, H.G.; Lee, S.Y.; Kim, N.R.; Lee, H.Y.; Ko, M.Y.; Jung, B.J.; Kim, C.M.; Lee, J.M.; Park, J.H.; Han, S.H.; et al. Lactobacillus plantarum lipoteichoic acid down-regulated Shigella flexneri peptidoglycan-induced inflammation. *Mol Immunol* **2011**, *48*, 382-391, doi:10.1016/j.molimm.2010.07.011.
22. Kim, J.Y.; Kim, H.; Jung, B.J.; Kim, N.R.; Park, J.E.; Chung, D.K. Lipoteichoic acid isolated from Lactobacillus plantarum suppresses LPS-mediated atherosclerotic plaque inflammation. *Mol Cells* **2013**, *35*, 115-124, doi:10.1007/s10059-013-2190-3.
23. Kang, S.S.; Ryu, Y.H.; Baik, J.E.; Yun, C.H.; Lee, K.; Chung, D.K.; Han, S.H. Lipoteichoic acid from Lactobacillus plantarum induces nitric oxide production in the presence of interferon-gamma in murine macrophages. *Mol Immunol* **2011**, *48*, 2170-2177, doi:10.1016/j.molimm.2011.07.009.
24. Jeon, B.; Kim, H.R.; Kim, H.; Chung, D.K. In vitro and in vivo downregulation of C3 by lipoteichoic acid isolated from Lactobacillus plantarum K8 suppressed cytokine-mediated complement system activation. *FEMS Microbiol Lett* **2016**, *363*, doi:10.1093/femsle/fnw140.
25. Kim, K.W.; Kang, S.S.; Woo, S.J.; Park, O.J.; Ahn, K.B.; Song, K.D.; Lee, H.K.; Yun, C.H.; Han, S.H. Lipoteichoic Acid of Probiotic Lactobacillus plantarum Attenuates Poly I:C-Induced IL-8 Production in Porcine Intestinal Epithelial Cells. *Front Microbiol* **2017**, *8*, 1827, doi:10.3389/fmicb.2017.01827.

26. Hatano, S.; Hirose, Y.; Yamamoto, Y.; Murosaki, S.; Yoshikai, Y. Scavenger receptor for lipoteichoic acid is involved in the potent ability of Lactobacillus plantarum strain L-137 to stimulate production of interleukin-12p40. *Int Immunopharmacol* **2015**, *25*, 321-331, doi:10.1016/j.intimp.2015.02.011.
27. Mizuno, H.; Arce, L.; Tomotsune, K.; Albarracin, L.; Funabashi, R.; Vera, D.; Islam, M.A.; Vizoso-Pinto, M.G.; Takahashi, H.; Sasaki, Y.; et al. Lipoteichoic Acid Is Involved in the Ability of the Immunobiotic Strain Lactobacillus plantarum CRL1506 to Modulate the Intestinal Antiviral Innate Immunity Triggered by TLR3 Activation. *Front Immunol* **2020**, *11*, 571, doi:10.3389/fimmu.2020.00571.
28. Ciszek-Lenda, M.; Nowak, B.; Srottek, M.; Walczewska, M.; Gorska-Fraczek, S.; Gamian, A.; Marcinkiewicz, J. Further studies on immunomodulatory effects of exopolysaccharide isolated from Lactobacillus rhamnosus KL37C. *Central European Journal of Immunology* **2013**, *38*, 289-298, doi:10.5114/ceji.2013.37743.
29. Friedrich, A.D.; Leoni, J.; Paz, M.L.; Gonzalez Maglio, D.H. Lipoteichoic Acid from Lacticaseibacillus rhamnosus GG Modulates Dendritic Cells and T Cells in the Gut. *Nutrients* **2022**, *14*, doi:10.3390/nu14030723.
30. Riehl, T.E.; Alvarado, D.; Ee, X.; Zuckerman, A.; Foster, L.; Kapoor, V.; Thotala, D.; Ciorba, M.A.; Stenson, W.F. Lactobacillus rhamnosus GG protects the intestinal epithelium from radiation injury through release of lipoteichoic acid, macrophage activation and the migration of mesenchymal stem cells. *Gut* **2019**, *68*, 1003-1013, doi:10.1136/gutjnl-2018-316226.
31. Mohamadzadeh, M.; Pfeiler, E.A.; Brown, J.B.; Zadeh, M.; Gramarossa, M.; Managlia, E.; Bere, P.; Sarraj, B.; Khan, M.W.; Pakanati, K.C.; et al. Regulation of induced colonic inflammation by Lactobacillus acidophilus deficient in lipoteichoic acid. *Proc Natl Acad Sci U S A* **2011**, *108 Suppl 1*, 4623-4630, doi:10.1073/pnas.1005066107.
32. Khan, M.W.; Zadeh, M.; Bere, P.; Gounaris, E.; Owen, J.; Klaenhammer, T.; Mohamadzadeh, M. Modulating intestinal immune responses by lipoteichoic acid-deficient Lactobacillus acidophilus. *Immunotherapy* **2012**, *4*, 151-161, doi:10.2217/imt.11.163.
33. Lee, B.; Yin, X.; Griffey, S.M.; Marco, M.L. Attenuation of Colitis by Lactobacillus casei BL23 Is Dependent on the Dairy Delivery Matrix. *Appl Environ Microbiol* **2015**, *81*, 6425-6435, doi:10.1128/AEM.01360-15.
34. Chen, K.; Luo, H.; Li, Y.; Han, X.; Gao, C.; Wang, N.; Lu, F.; Wang, H. Lactobacillus paracasei TK1501 fermented soybeans alleviate dextran sulfate sodium-induced colitis by regulating intestinal cell function. *J Sci Food Agric* **2023**, *103*, 5422-5431, doi:10.1002/jsfa.12615.
35. Smelt, M.J.; de Haan, B.J.; Bron, P.A.; van Swam, I.; Meijerink, M.; Wells, J.M.; Kleerebezem, M.; Faas, M.M.; de Vos, P. The impact of Lactobacillus plantarum WCFS1 teichoic acid D-alanylation on the generation of effector and regulatory T-cells in healthy mice. *PLoS One* **2013**, *8*, e63099, doi:10.1371/journal.pone.0063099.
36. Ganguli, K.; Collado, M.C.; Rautava, J.; Lu, L.; Satokari, R.; von Ossowski, I.; Reunanen, J.; de Vos, W.M.; Palva, A.; Isolauri, E.; et al. Lactobacillus rhamnosus GG and its SpaC pilus adhesin modulate inflammatory responsiveness and TLR-related gene expression in the fetal human gut. *Pediatr Res* **2015**, *77*, 528-535, doi:10.1038/pr.2015.5.

37. Tytgat, H.L.; van Teijlingen, N.H.; Sullan, R.M.; Douillard, F.P.; Rasinkangas, P.; Messing, M.; Reunanen, J.; Satokari, R.; Vanderleyden, J.; Dufrene, Y.F.; et al. Probiotic Gut Microbiota Isolate Interacts with Dendritic Cells via Glycosylated Heterotrimeric Pili. *PLoS One* **2016**, *11*, e0151824, doi:10.1371/journal.pone.0151824.
38. Lebeer, S.; Claes, I.; Tytgat, H.L.; Verhoeven, T.L.; Marien, E.; von Ossowski, I.; Reunanen, J.; Palva, A.; Vos, W.M.; Keersmaecker, S.C.; et al. Functional analysis of Lactobacillus rhamnosus GG pili in relation to adhesion and immunomodulatory interactions with intestinal epithelial cells. *Appl Environ Microbiol* **2012**, *78*, 185-193, doi:10.1128/AEM.06192-11.
39. Zhang, L.; Liu, C.; Li, D.; Zhao, Y.; Zhang, X.; Zeng, X.; Yang, Z.; Li, S. Antioxidant activity of an exopolysaccharide isolated from Lactobacillus plantarum C88. *Int J Biol Macromol* **2013**, *54*, 270-275, doi:10.1016/j.ijbiomac.2012.12.037.
40. Wang, X.; Shao, C.; Liu, L.; Guo, X.; Xu, Y.; Lu, X. Optimization, partial characterization and antioxidant activity of an exopolysaccharide from Lactobacillus plantarum KX041. *Int J Biol Macromol* **2017**, *103*, 1173-1184, doi:10.1016/j.ijbiomac.2017.05.118.
41. Huang, Y.Y.; Wu, J.M.; Wu, W.T.; Lin, J.W.; Liang, Y.T.; Hong, Z.Z.; Jia, X.Z.; Liu, D.M. Structural, antioxidant, and immunomodulatory activities of an acidic exopolysaccharide from Lactiplantibacillus plantarum DMDL 9010. *Front Nutr* **2022**, *9*, 1073071, doi:10.3389/fnut.2022.1073071.
42. Zhou, X.; Qi, W.; Hong, T.; Xiong, T.; Gong, D.; Xie, M.; Nie, S. Exopolysaccharides from Lactobacillus plantarum NCU116 Regulate Intestinal Barrier Function via STAT3 Signaling Pathway. *J Agric Food Chem* **2018**, *66*, 9719-9727, doi:10.1021/acs.jafc.8b03340.
43. Lee, K.; Kim, H.J.; Kim, S.A.; Park, S.D.; Shim, J.J.; Lee, J.L. Exopolysaccharide from Lactobacillus plantarum HY7714 Protects against Skin Aging through Skin-Gut Axis Communication. *Molecules* **2021**, *26*, doi:10.3390/molecules26061651.
44. Kwon, M.; Lee, J.; Park, S.; Kwon, O.H.; Seo, J.; Roh, S. Exopolysaccharide Isolated from Lactobacillus plantarum L-14 Has Anti-Inflammatory Effects via the Toll-Like Receptor 4 Pathway in LPS-Induced RAW 264.7 Cells. *Int J Mol Sci* **2020**, *21*, doi:10.3390/ijms21239283.
45. Rajoka, M.S.R.; Mehwish, H.M.; Kitazawa, H.; Barba, F.J.; Berthelot, L.; Umair, M.; Zhu, Q.; He, Z.; Zhao, L. Techno-functional properties and immunomodulatory potential of exopolysaccharide from Lactiplantibacillus plantarum MM89 isolated from human breast milk. *Food Chem* **2022**, *377*, 131954, doi:10.1016/j.foodchem.2021.131954.
46. Srutkova, D.; Kozakova, H.; Novotna, T.; Gorska, S.; Hermanova, P.P.; Hudcovic, T.; Svabova, T.; Sinkora, M.; Schwarzer, M. Exopolysaccharide from Lacticaseibacillus rhamnosus induces IgA production in airways and alleviates allergic airway inflammation in mouse model. *Eur J Immunol* **2023**, *53*, e2250135, doi:10.1002/eji.202250135.
47. Kumari, M.; Dasriya, V.L.; Nataraj, B.H.; Nagpal, R.; Behare, P.V. Lacticaseibacillus rhamnosus-Derived Exopolysaccharide Attenuates D-Galactose-Induced Oxidative Stress and Inflammatory Brain Injury and Modulates Gut Microbiota in a Mouse Model. *Microorganisms* **2022**, *10*, doi:10.3390/microorganisms10102046.
48. Li, J.; Li, Q.; Wu, Q.; Gao, N.; Wang, Z.; Yang, Y.; Shan, A. Exopolysaccharides of Lactobacillus rhamnosus GG ameliorate Salmonella typhimurium-induced intestinal inflammation via the TLR4/NF-kappaB/MAPK pathway. *J Anim Sci Biotechnol* **2023**, *14*, 23, doi:10.1186/s40104-023-00830-7.

49. Khedr, O.M.S.; El-Sonbaty, S.M.; Moawed, F.S.M.; Kandil, E.I.; Abdel-Maksoud, B.E. Lactobacillus acidophilus ATCC 4356 Exopolysaccharides Suppresses Mediators of Inflammation through the Inhibition of TLR2/STAT-3/P38-MAPK Pathway in DEN-Induced Hepatocarcinogenesis in Rats. *Nutr Cancer* **2022**, *74*, 1037-1047, doi:10.1080/01635581.2021.1934490.
50. Li, L.; Jiang, Y.J.; Yang, X.Y.; Liu, Y.; Wang, J.Y.; Man, C.X. Immunoregulatory effects on Caco-2 cells and mice of exopolysaccharides isolated from Lactobacillus acidophilus NCFM. *Food Funct* **2014**, *5*, 3261-3268, doi:10.1039/c4fo00565a.
51. Xu, X.; Qiao, Y.; Peng, Q.; Shi, B.; Dia, V.P. Antioxidant and Immunomodulatory Properties of Partially purified Exopolysaccharide from Lactobacillus Casei Isolated from Chinese Northeast Sauerkraut. *Immunol Invest* **2022**, *51*, 748-765, doi:10.1080/08820139.2020.1869777.
52. Makino, S.; Sato, A.; Goto, A.; Nakamura, M.; Ogawa, M.; Chiba, Y.; Hemmi, J.; Kano, H.; Takeda, K.; Okumura, K.; et al. Enhanced natural killer cell activation by exopolysaccharides derived from yogurt fermented with Lactobacillus delbrueckii ssp. bulgaricus OLL1073R-1. *J Dairy Sci* **2016**, *99*, 915-923, doi:10.3168/jds.2015-10376.
53. You, X.; Li, Z.; Ma, K.; Zhang, C.; Chen, X.; Wang, G.; Yang, L.; Dong, M.; Rui, X.; Zhang, Q.; et al. Structural characterization and immunomodulatory activity of an exopolysaccharide produced by Lactobacillus helveticus LZ-R-5. *Carbohydr Polym* **2020**, *235*, 115977, doi:10.1016/j.carbpol.2020.115977.
54. Noda, M.; Danshiitsoodol, N.; Kanno, K.; Uchida, T.; Sugiyama, M. The Exopolysaccharide Produced by Lactobacillus paracasei IJH-SONE68 Prevents and Ameliorates Inflammatory Responses in DSS-Induced Ulcerative Colitis. *Microorganisms* **2021**, *9*, doi:10.3390/microorganisms9112243.
55. Kissova, Z.; Tkacikova, L.; Mudronova, D.; Bhide, M.R. Immunomodulatory Effect of Lactobacillus reuteri (Limosilactobacillus reuteri) and Its Exopolysaccharides Investigated on Epithelial Cell Line IPEC-J2 Challenged with Salmonella Typhimurium. *Life (Basel)* **2022**, *12*, doi:10.3390/life12121955.
56. Kissova, Z.; Schusterova, P.; Mudronova, D.; Novotny, J.; Tkacikova, L. Exopolysaccharides from Limosilactobacillus reuteri: their influence on in vitro activation of porcine monocyte-derived dendritic cells - brief report. *Vet Res Commun* **2024**, doi:10.1007/s11259-024-10445-6.
57. Kissova, Z.; Mudronova, D.; Link, R.; Tkacikova, L. Immunomodulatory effect of probiotic exopolysaccharides in a porcine in vitro co-culture model mimicking the intestinal environment on ETEC infection. *Vet Res Commun* **2024**, *48*, 705-724, doi:10.1007/s11259-023-10237-4.
58. Ksonzekova, P.; Bystricky, P.; Vlckova, S.; Patoprsty, V.; Pulzova, L.; Mudronova, D.; Kubaskova, T.; Csank, T.; Tkacikova, L. Exopolysaccharides of Lactobacillus reuteri: Their influence on adherence of E. coli to epithelial cells and inflammatory response. *Carbohydr Polym* **2016**, *141*, 10-19, doi:10.1016/j.carbpol.2015.12.037.
59. Gao, X.; Huang, L.; Zhu, L.; Mou, C.; Hou, Q.; Yu, Q. Inhibition of H9N2 Virus Invasion into Dendritic Cells by the S-Layer Protein from L. acidophilus ATCC 4356. *Front Cell Infect Microbiol* **2016**, *6*, 137, doi:10.3389/fcimb.2016.00137.
60. Klotz, C.; Goh, Y.J.; O'Flaherty, S.; Barrangou, R. S-layer associated proteins contribute to the adhesive and immunomodulatory properties of Lactobacillus acidophilus NCFM. *BMC Microbiol* **2020**, *20*, 248, doi:10.1186/s12866-020-01908-2.

61. Konstantinov, S.R.; Smidt, H.; de Vos, W.M.; Bruijns, S.C.; Singh, S.K.; Valence, F.; Molle, D.; Lortal, S.; Altermann, E.; Klaenhammer, T.R.; et al. S layer protein A of Lactobacillus acidophilus NCFM regulates immature dendritic cell and T cell functions. *Proc Natl Acad Sci U S A* **2008**, *105*, 19474-19479, doi:10.1073/pnas.0810305105.
62. Wang, H.; Zhang, Q.; Niu, Y.; Zhang, X.; Lu, R. Surface-layer protein from Lactobacillus acidophilus NCFM attenuates tumor necrosis factor-alpha-induced intestinal barrier dysfunction and inflammation. *Int J Biol Macromol* **2019**, *136*, 27-34, doi:10.1016/j.ijbiomac.2019.06.041.
63. Li, P.; Yu, Q.; Ye, X.; Wang, Z.; Yang, Q. Lactobacillus S-layer protein inhibition of Salmonella-induced reorganization of the cytoskeleton and activation of MAPK signalling pathways in Caco-2 cells. *Microbiology (Reading)* **2011**, *157*, 2639-2646, doi:10.1099/mic.0.049148-0.
64. Zhang, X.; Li, Y.; Zhang, C.; Chi, H.; Liu, C.; Li, A.; Yu, W. Postbiotics derived from Lactobacillus plantarum 1.0386 ameliorate lipopolysaccharide-induced tight junction injury via MicroRNA-200c-3p mediated activation of the MLCK-MLC pathway in Caco-2 cells. *Food Funct* **2022**, *13*, 11008-11020, doi:10.1039/d2fo00001f.
65. Yin, M.; Yan, X.; Weng, W.; Yang, Y.; Gao, R.; Liu, M.; Pan, C.; Zhu, Q.; Li, H.; Wei, Q.; et al. Micro Integral Membrane Protein (MIMP), a Newly Discovered Anti-Inflammatory Protein of Lactobacillus Plantarum, Enhances the Gut Barrier and Modulates Microbiota and Inflammatory Cytokines. *Cell Physiol Biochem* **2018**, *45*, 474-490, doi:10.1159/000487027.
66. Lee, I.C.; van, S., II; Boeren, S.; Vervoort, J.; Meijerink, M.; Taverne, N.; Starrenburg, M.; Bron, P.A.; Kleerebezem, M. Lipoproteins Contribute to the Anti-inflammatory Capacity of Lactobacillus plantarum WCFS1. *Front Microbiol* **2020**, *11*, 1822, doi:10.3389/fmicb.2020.01822.
67. Kim, B.S.; Yun, C.H.; Han, S.H.; Song, K.D.; Kang, S.S. Inhibitory Effect of Lipoteichoic Acid Derived from Three Lactobacilli on Flagellin-Induced IL-8 Production in Porcine Peripheral Blood Mononuclear Cells. *Probiotics Antimicrob Proteins* **2021**, *13*, 72-79, doi:10.1007/s12602-020-09682-3.
68. Shigemori, S.; Namai, F.; Ogita, T.; Sato, T.; Shimosato, T. Oral priming with oligodeoxynucleotide particles from Lactobacillus rhamnosus GG attenuates symptoms of dextran sodium sulfate-induced acute colitis in mice. *Anim Sci J* **2020**, *91*, e13468, doi:10.1111/asj.13468.
69. Gao, K.; Wang, C.; Liu, L.; Dou, X.; Liu, J.; Yuan, L.; Zhang, W.; Wang, H. Immunomodulation and signaling mechanism of Lactobacillus rhamnosus GG and its components on porcine intestinal epithelial cells stimulated by lipopolysaccharide. *J Microbiol Immunol Infect* **2017**, *50*, 700-713, doi:10.1016/j.jmii.2015.05.002.
70. Luyer, M.D.; Buurman, W.A.; Hadfoune, M.; Speelmans, G.; Knol, J.; Jacobs, J.A.; Dejong, C.H.; Vriesema, A.J.; Greve, J.W. Strain-specific effects of probiotics on gut barrier integrity following hemorrhagic shock. *Infect Immun* **2005**, *73*, 3686-3692, doi:10.1128/IAI.73.6.3686-3692.2005.
71. Qi, S.R.; Cui, Y.J.; Liu, J.X.; Luo, X.; Wang, H.F. Lactobacillus rhamnosus GG components, SLP, gDNA and CpG, exert protective effects on mouse macrophages upon lipopolysaccharide challenge. *Lett Appl Microbiol* **2020**, *70*, 118-127, doi:10.1111/lam.13255.

72. Champagne-Jorgensen, K.; Jose, T.A.; Stanisz, A.M.; Mian, M.F.; Hynes, A.P.; Bienenstock, J. Bacterial membrane vesicles and phages in blood after consumption of lacticaseibacillus rhamnosus JB-1. *Gut Microbes* **2021**, *13*, 1993583, doi:10.1080/19490976.2021.1993583.
73. Mata Forsberg, M.; Bjorkander, S.; Pang, Y.; Lundqvist, L.; Ndi, M.; Ott, M.; Escriba, I.B.; Jaeger, M.C.; Roos, S.; Sverremark-Ekstrom, E. Extracellular Membrane Vesicles from Lactobacilli Dampen IFN-gamma Responses in a Monocyte-Dependent Manner. *Sci Rep* **2019**, *9*, 17109, doi:10.1038/s41598-019-53576-6.
74. Hu, R.; Lin, H.; Wang, M.; Zhao, Y.; Liu, H.; Min, Y.; Yang, X.; Gao, Y.; Yang, M. Lactobacillus reuteri-derived extracellular vesicles maintain intestinal immune homeostasis against lipopolysaccharide-induced inflammatory responses in broilers. *J Anim Sci Biotechnol* **2021**, *12*, 25, doi:10.1186/s40104-020-00532-4.
75. Pang, Y.; Ermann Lundberg, L.; Mata Forsberg, M.; Ahl, D.; Bysell, H.; Pallin, A.; Sverremark-Ekstrom, E.; Karlsson, R.; Jonsson, H.; Roos, S. Extracellular membrane vesicles from Limosilactobacillus reuteri strengthen the intestinal epithelial integrity, modulate cytokine responses and antagonize activation of TRPV1. *Front Microbiol* **2022**, *13*, 1032202, doi:10.3389/fmicb.2022.1032202.
76. Yamasaki-Yashiki, S.; Kawashima, F.; Saika, A.; Hosomi, R.; Kunisawa, J.; Katakura, Y. RNA-Based Anti-Inflammatory Effects of Membrane Vesicles Derived from Lactiplantibacillus plantarum. *Foods* **2024**, *13*, doi:10.3390/foods13060967.
